# Matrix Drug Screen Identifies Synergistic Drug Combinations to Augment SMAC Mimetic Activity in Ovarian Cancer

**DOI:** 10.3390/cancers12123784

**Published:** 2020-12-15

**Authors:** Anne M. Noonan, Amanda Cousins, David Anderson, Kristen P. Zeligs, Kristen Bunch, Lidia Hernandez, Yusuke Shibuya, Ian S. Goldlust, Rajarshi Guha, Marc Ferrer, Craig J. Thomas, Christina M. Annunziata

**Affiliations:** 1 Women’s Malignancies Branch, National Cancer Institute, National Institutes of Health, Bethesda, MD 20892, USA; Anne.Noonan@osumc.edu (A.M.N.); amanda.cousins@nih.gov (A.C.); dmotocycle@gmail.com (D.A.); kristenzeligs@gmail.com (K.P.Z.); kristen.p.bunch.mil@mail.mil (K.B.); hernandli@mail.nih.gov (L.H.); yusuke.shibuya@nih.gov (Y.S.); 2 Department of Internal Medicine, Division of Medical Oncology, The Ohio State University, James Cancer Hospital and Solve Research Institute, Columbus, OH 43210, USA; 3 Gynecologic Cancer Center of Excellence, Department of Obstetrics and Gynecology, Uniformed Services University and Walter Reed National Military Medical Center, Bethesda, MD 20814, USA; 4 Department of Obstetrics, Gynecology, and Reproductive Science, Division of Gynecologic Oncology, Icahn School of Medicine at Mount Sinai, New York City, NY 10029, USA; 5 Department of Obstetrics and Gynecology, Division of Gynecology Oncology, Tripler Army Medical Center, Honolulu, HI 96859, USA; 6 The National Center for Advancing Translational Sciences, National Institutes of Health, Bethesda, MD 20892, USA; igoldlust@gmail.com (I.S.G.); rajarshi_guha@vrtx.com (R.G.); marc.ferrer@nih.gov (M.F.); craigt@mail.nih.gov (C.J.T.)

**Keywords:** ovarian cancer, SMAC mimetics, birinapant, docetaxel, synergy, TNF-α, NF-kB, microtubule stability

## Abstract

**Simple Summary:**

Recurrent ovarian cancer is difficult to treat due to the development of chemotherapy resistance. This resistance develops through multiple mechanisms to include the avoidance of cell death by cancer cells. Prior studies have shown birinapant, a second mitochondrial activator of caspases (SMAC) mimetic drug, to be promising in overcoming this acquired resistance. Despite good tolerability, however, therapy with single-agent birinapant exhibited minimal anti-cancer activity in women with recurrent ovarian cancer. By using a high-throughput drug screen we were able to identify potential therapeutic agents that augment birinapant activity, with docetaxel emerging favorably due to its marked synergy and known utility in the recurrent ovarian cancer setting. We showed that this synergy is the result of several complementary molecular pathways and hope to highlight the promising potential of this therapeutic drug combination for clinical testing where treatment options are often limited.

**Abstract:**

Inhibitor of apoptosis (IAP) proteins are frequently upregulated in ovarian cancer, resulting in the evasion of apoptosis and enhanced cellular survival. Birinapant, a synthetic second mitochondrial activator of caspases (SMAC) mimetic, suppresses the functions of IAP proteins in order to enhance apoptotic pathways and facilitate tumor death. Despite on-target activity, however, pre-clinical trials of single-agent birinapant have exhibited minimal activity in the recurrent ovarian cancer setting. To augment the therapeutic potential of birinapant, we utilized a high-throughput screening matrix to identify synergistic drug combinations. Of those combinations identified, birinapant plus docetaxel was selected for further evaluation, given its remarkable synergy both in vitro and in vivo. We showed that this synergy results from multiple convergent pathways to include increased caspase activation, docetaxel-mediated TNF-α upregulation, alternative NF-kB signaling, and birinapant-induced microtubule stabilization. These findings provide a rationale for the integration of birinapant and docetaxel in a phase 2 clinical trial for recurrent ovarian cancer where treatment options are often limited and minimally effective.

## 1. Introduction

Ovarian cancer is the most lethal gynecologic malignancy, with a five-year survival of just under 50% [1]. Nearly 75% of women with ovarian cancer will be diagnosed with advanced stage disease at the time of presentation, portending an overall poor prognosis. The current standard of care treatment includes cytoreductive surgery in conjunction with platinum plus taxane-based chemotherapy [2]. Despite high initial response rates, tumor recurrence is common due to the development of drug resistance and the ability of tumor cells to evade apoptosis [3].

Apoptosis is one type of regulated cell death that occurs via an extrinsic (death receptor-ligand) or intrinsic (mitochondrial) pathway, resulting in the initiation of a proteolytic cascade and ultimately the activation of caspases [4]. To prevent inappropriate activation of these pathways, cell death is strictly regulated by both pro-apoptotic and anti-apoptotic proteins. Inhibitor of apoptosis (IAP) proteins are one such regulator that block executioner caspase function [5]. Second mitochondrial activator of caspases (SMAC) proteins, on the other hand, antagonize IAP proteins, thereby promoting cell death. 

Many cancers express high levels of IAP proteins, resulting in defective apoptosis, enhanced cell survival, and chemotherapy resistance [6,7]. For this reason, IAP proteins have become an important target for therapeutic intervention. Synthetic molecules that mimic endogenous SMAC (SMAC mimetics) have emerged as a promising targeted cancer therapy to engage cell death signaling pathways [8]. Birinapant, a synthetic divalent peptide, is one such SMAC mimetic. In both pre-clinical and clinical models, birinapant depleted cIAP1 and cIAP2, activated caspase 3, and inhibited tumor cell growth [9,10]. 

The NF-kB signaling pathway is another important target of SMAC mimetic therapy. NF-kB is a pro-inflammatory and pro-survival pathway that is constitutively active in ovarian cancer [11]. Activation of NF-kB can occur via one of two pathways, the classical or alternative [12]. In the presence of cIAP1 and cIAP2, TNF-α signaling leads to activation of the classical NF-kB signaling pathway, resulting in cellular proliferation, cytokine production, and anti-apoptosis gene expression. In the absence of cIAP1 and cIAP2, TNF-α signaling leads to caspase 8 activation and cellular apoptosis [13]. Overexpression of TNF-α is common in ovarian cancer, and in the presence of SMAC mimetic therapy, may drive cancer cells towards apoptosis [14,15]. 

Despite on-target activity and good tolerability, birinapant demonstrated minimal single-agent activity in recurrent ovarian cancer [11]. When combined with other cytotoxic therapies, however, synergistic cooperation occurred with greater sensitization of tumor cells to cytotoxic treatment [16,17]. 

With this study, we aimed to identify therapeutic agents that, when combined with birinapant, synergized to produce the greatest therapeutic potential. A combination drug screening platform was utilized to narrow our focus of the study by rapidly identifying those combinations with potential synergistic activity [18]. This synergism was confirmed by further in vitro testing. Of the synergistic combinations studied, birinapant plus docetaxel stood out, given its remarkable synergy across multiple ovarian cancer cell lines both in vitro and in 2 xenograft models. This combination was particularly appealing for clinical application given its proven safety in the phase 1 b study [19] and potential for augmenting the therapeutic use of docetaxel in the recurrent ovarian cancer setting. We proposed a possible mechanism of action to explain this synergy with a focus on apoptosis, alternative NF-kB signaling, and microtubule stabilization, with plans to integrate these findings into a phase 2 clinical trial. By identifying synergistic drug combinations, we hope to shed light on treatment options that may be more efficacious for women with chemorefractory ovarian cancer. 

## 2. Results

### 2.1. Birinapant Is Synergistic with Specific Classes of Drugs

**Figure 1:**
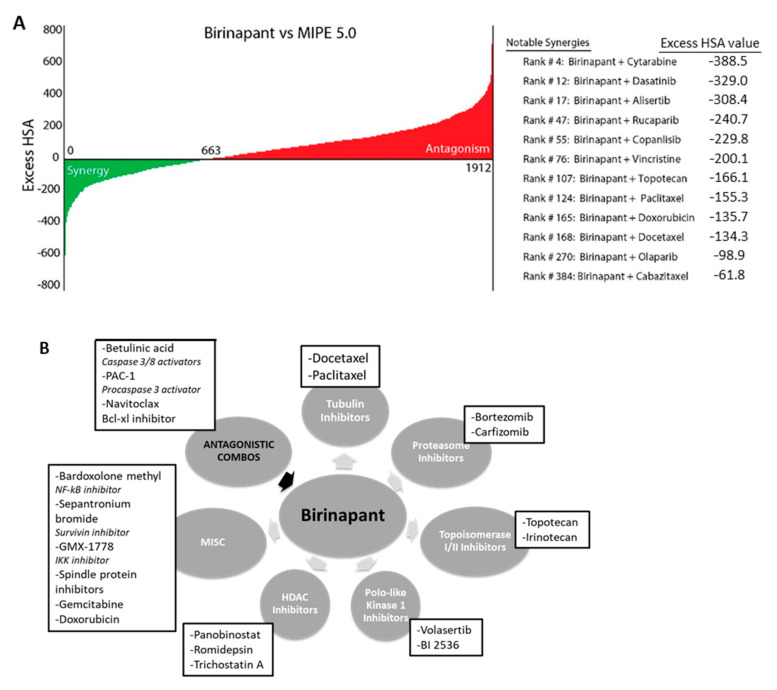

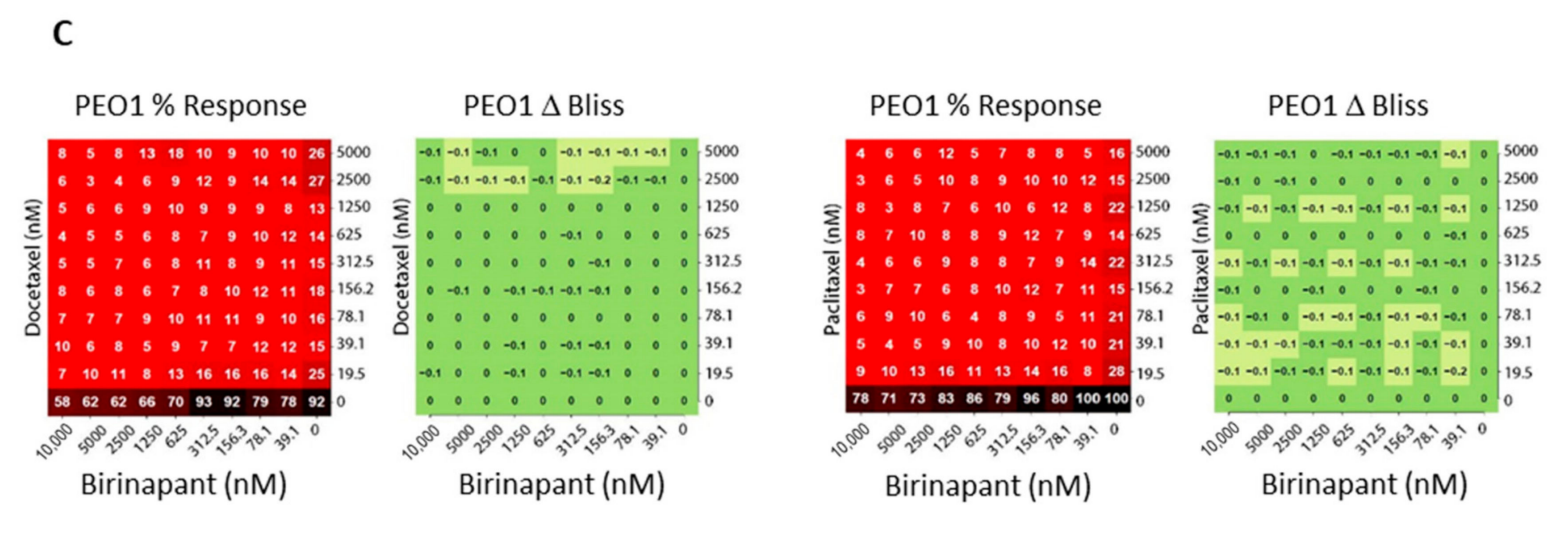
Birinapant is synergistic with specific classes of drugs. (**A**) Matrix screen was performed with PEO1 cell line. Excess HSA values are shown for each of the 1912 compounds in combination with birinapant. Clinically interesting combinations are listed at the right. (**B**) Drugs were categorized by mechanism of action and grouped based on matrix screen results. Shown are classes of drugs identified as having synergistic activity with birinapant. Classes fell into distinct categories such as taxanes, HDAC inhibitors, and PLK-1 inhibitors. (**C**) Matrix plots for docetaxel and paclitaxel showed a strong combination effect even at low concentrations of both drugs. Red/black plot shows % viability based on Caspase-Glo assay, and green plot shows delta Bliss where negative numbers indicate synergy.

A library of 1912 compounds [20] was selected to evaluate for synergy with the SMAC-mimetic birinapant. Compounds selected included FDA (Food and Drug Administration)-approved drugs, as well as clinical compounds in phase 1–3 human clinical trials [21]. The range for stepwise serial dilution of each compound was determined based on single-agent activity in cancer cell lines. PEO1, a high-grade serous ovarian cancer cell line, was chosen as it was technically reproducible in the robotic technology used for the high throughput assay and is representative of the most common ovarian cancer histology. The selected 6-step range was applied in triplicate in a matrix with birinapant, using the ovarian cancer cell line PEO-1, and synergy or antagonism was determined by Excess HAS (Highest Single Agent) (Figure 1A, Table S1) [22]. After 72 h, cell viability and apoptosis were measured separately. From the initial screen, 74 drugs were selected for further study based on a combination index <0.5 and beta parameter <1, calculated according to the Chou–Talalay method [23]. The 74 compounds were plated in a similar manner, in 10 × 10 matrix dilutions repeated in triplicate. The synergistic combinations identified fell into several reproducible categories of agents to include: Taxanes, proteasome inhibitors, topoisomerase inhibitors, PLK1 inhibitors, and HDAC inhibitors (Figure 1B, Table S2). The taxanes docetaxel and paclitaxel showed synergy even at the lowest concentrations tested (Figure 1C). Docetaxel was chosen from these categories for further study, based on its known activity in treating women with recurrent ovarian cancer [24].

### 2.2. Birinapant Is Synergistic in Combination with Docetaxel in Killing Ovarian Cancer Cells

**Figure 2:**
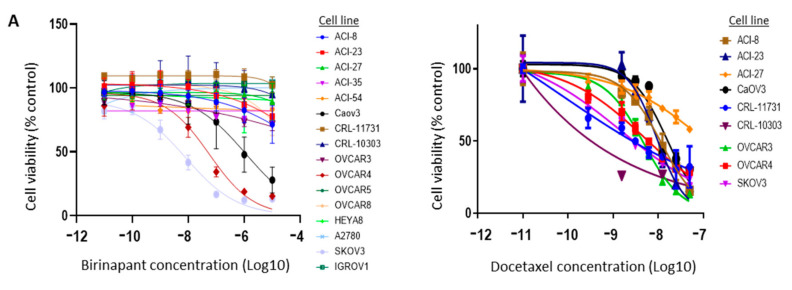

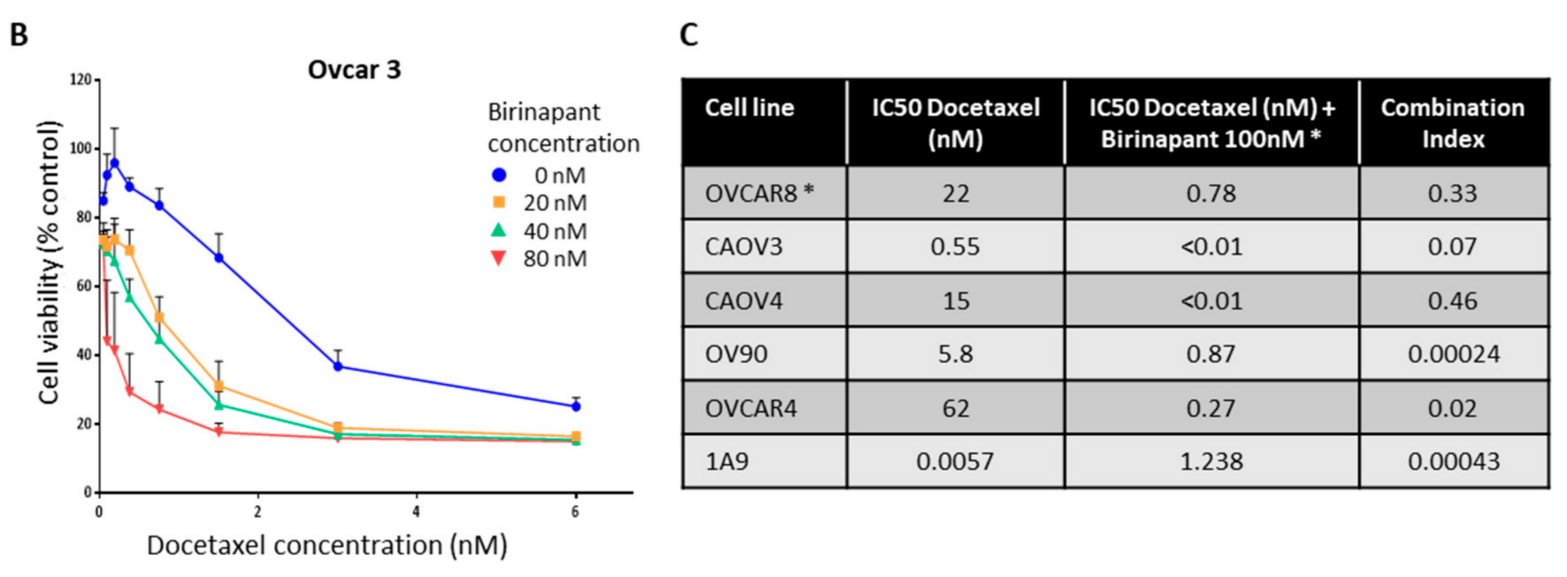
Birinapant is synergistic with docetaxel in vitro. (**A**) A panel of ovarian cancer cell lines was treated with increasing concentrations of single-agent birinapant (**left**) or docetaxel (**right**). (**B**) Cell viability of OVCAR3 cells was determined after treating with matrix titrations of birinapant and docetaxel. Shown are final values normalized as a percent of control (vehicle treatment). (**C**) IC50 values were calculated for docetaxel alone and for docetaxel when combined with birinapant (* 100 nM of docetaxel used for calculation of combination index in all cell lines except OVCAR 8 where 80 nM was used). The IC50 for birinapant alone was not included as all cell lines had minimal sensitivity to single-agent therapy. Error bars in (**A**) and (**B**) indicate standard error of the mean.

Dose titration of birinapant was carried out in 16 cell lines representing different histologies of ovarian cancer and found to have single-agent activity in three (CAOV3, OVCAR4, SKOV3) based on XTT cell viability assay (Figure 2A, left). Docetaxel was tested in 9 of the cell lines and showed a range of killing across several concentrations (Figure 2A, right). Doses above 10 micromolar were not considered meaningful due to potential off-target effects at high concentrations and a lack of clinical translatability. Synergy between birinapant and docetaxel was confirmed in vitro using matrix titrations of both drugs in OVCAR3 cells (Figure 2B). Six additional ovarian cancer cell lines were also found to have varying sensitivity to birinapant and docetaxel, as single agents, but marked synergy when birinapant was combined in matrix format with docetaxel (Figure 2C). This occurred across most dilutions and across all ovarian cancer cell lines tested, confirming the findings in the matrix drug screen.

**Figure 3:**
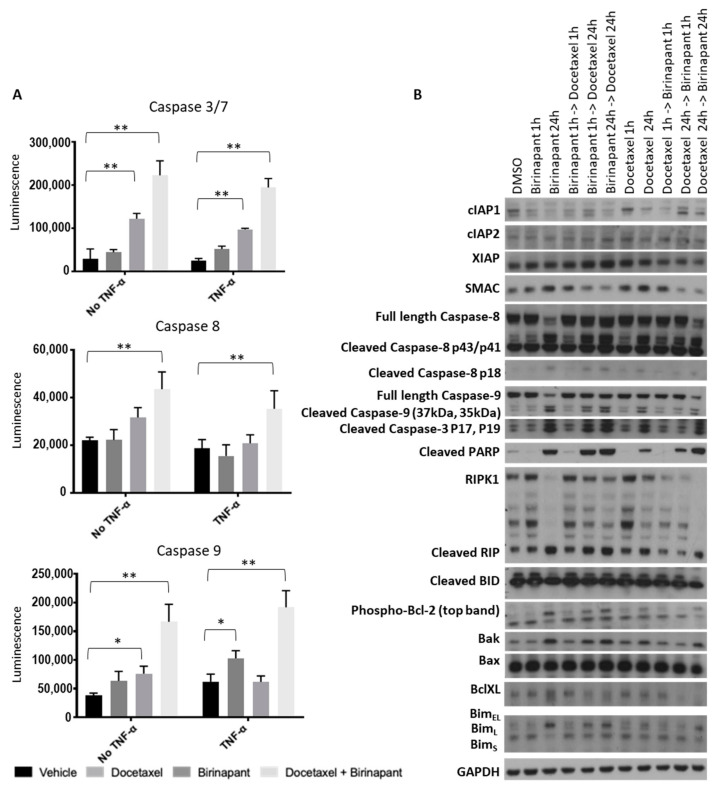
Docetaxel and birinapant synergistically increase cell death. (**A**) Caspase activation was quantified by luminescence assay as a surrogate for apoptotic activity. All luminescence data were normalized to cell viability as determined by XTT assay performed simultaneously. ANOVA multiple comparisons testing determined statistically significant results. * *p* < 0.005; ** *p* < 0.0001. Error bars indicate standard error of the mean. (**B**) Changes in apoptosis proteins were measured by western blot in CaOV3 after treatment with either single-agent or combination birinapant 200 and docetaxel 2.5 nM added sequentially or concurrently and with varying time points (1 vs. 24 h) as indicated.

The next goal was to quantify caspase activation as a surrogate for apoptotic activity. Caspase activation was quantified in OVCAR3 and OVCAR8 cells by relative luminescence (Figure 3A). All activity data was normalized to cell viability by XTT assay. Experiments were performed both with TNF-α and without, given the known interaction between SMAC mimetics and TNF-α signaling [25]. Caspase 3/7 was measured as representing the final common pathway of apoptosis, Caspase 8 as extrinsic apoptosis, and Caspase 9 as intrinsic apoptosis. Interestingly, both intrinsic and extrinsic apoptosis appeared to be triggered by the drug combination. As expected, the combination of birinapant and docetaxel increased activation of all caspases to a greater extent than each single agent.

Changes in proteins of the apoptosis pathways were measured qualitatively by western blot after cells were exposed to single agents, and combinations of birinapant and docetaxel were added either simultaneously or sequentially (Figure 3B and Figure S2). Depletion of cIAP1 confirmed the on-target effect of birinapant. We tested an abbreviated time course of each drug, and we also sought to determine whether the order of drug administration would change the effect on triggering apoptotic pathways. The combination demonstrated the strongest activation pattern in these proteins, regardless of the order of administration. In either order, the longer exposure to birinapant was required for maximal caspase and PARP cleavage. These data confirm the pro-apoptotic activity of this drug combination. Similar results were obtained with SKOV3 and OVCAR4 (Figure S1A,B respectively and Figure S4).

### 2.3. Docetaxel-Induced TNF-α Production in Tumor Cells Is Important for Synergy with Birinapant

**Figure 4:**
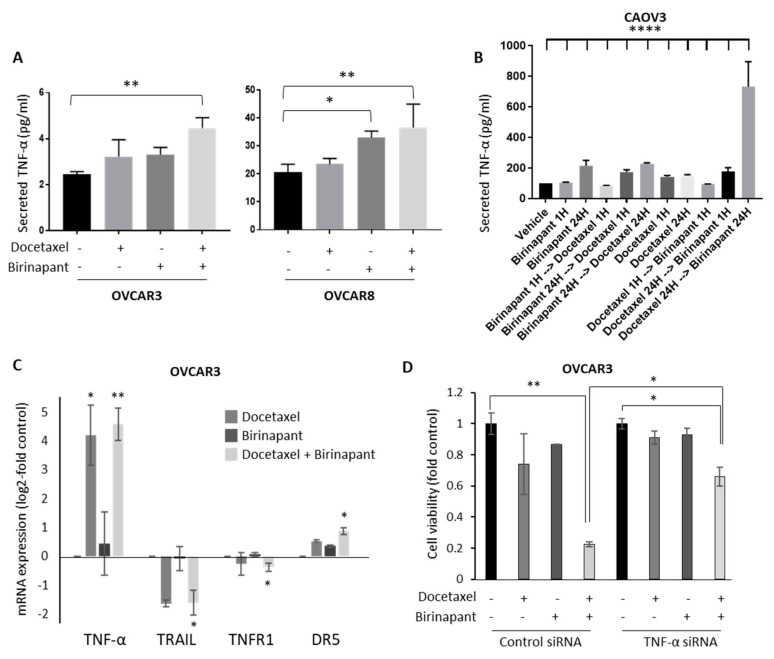
Docetaxel-induced TNF-α production in tumor cells is important for synergy with birinapant. (**A**) Secreted levels of TNF-α were measured by ELISA in culture supernatants after treating OVCAR3 and OVCAR8 cells overnight with single-agent docetaxel 4 nM or in combination with birinapant 400 nM. ANOVA multiple comparisons testing determined statistically significant results. * *p* < 0.05; ** *p* < 0.01. (**B**) TNF-α secretion was measured in medium from CaOV3 cells after sequential administration of birinapant 200 and docetaxel 2.5 nM. Short exposure (1 h) to combination therapy and longer exposure (24 h) to sequential administration of docetaxel followed by birinapant were performed. Levels were normalized to untreated cells. ANOVA multiple comparisons testing determined statistically significant results between docetaxel 24 ≥ birinapant 24 h and all other groups. **** *p* < 0.0001 (**C**) Changes in TNFa mRNA expression were measured by quantitative PCR in cells treated with the combination of birinapant 400 nM and docetaxel 2 nM (24-H treatment). Results are expressed as log2-fold vehicle treated control. * *p* < 0.05; ** *p* < 0.01 (**D**) To demonstrate the importance of TNF-α in synergistic killing, TNF mRNA was knocked down using siRNA. Cells transduced with siTNF-α or control were exposed for 72 h to docetaxel 4 nM with or without birinapant 400 nM. * *p* < 0.05; ** *p* < 0.01. Error bars in all panels indicate standard error of the mean.

Cytokine production was quantified as a measure of NF-kB-induced gene expression. Secreted cytokine levels of IL-1ß, IL-6, IL-8, and TNF-α were measured in culture supernatants after treating cells with docetaxel alone, birinapant alone, or both drugs in combination. The production of IL-1ß, IL-6, and IL-8 was minimally varied across treatment groups and did not reach statistical significance (not shown). There was, however, a notable and statistically significant increase in TNF-α production after treatment of cells with a single-agent drug that further increased with the combination (Figure 4A). TNF-α is regulated by NF-kB, among other transcription factors. Sequential administration of birinapant and docetaxel did not affect TNF-α secretion in short (1-h) exposure. In longer (24-h) exposure, docetaxel followed by birinapant induced more TNF-α secretion than the reverse order (Figure 4B). The combination effect was confirmed at the mRNA level, showing a dramatic increase in TNF-α mRNA induced predominantly by docetaxel, and a smaller increase in DR5 production with the combination (Figure 4C). TRAIL and TNFR1 messages were not affected by single agents but were significantly downregulated by the combination. In order to demonstrate the importance of TNF-α in the synergistic effect on killing, TNF-α mRNA was knocked down with siRNA. Cells transduced with siTNF-α or control were exposed to docetaxel with or without birinapant. Importantly, knockdown of TNF-α with RNA interference prevented the synergistic killing of birinapant combined with docetaxel (Figure 4D).

### 2.4. Birinapant Suppresses the Classical NF-kB Pathway and Enhances the Alternative NF-kB Pathway

**Figure 5:**
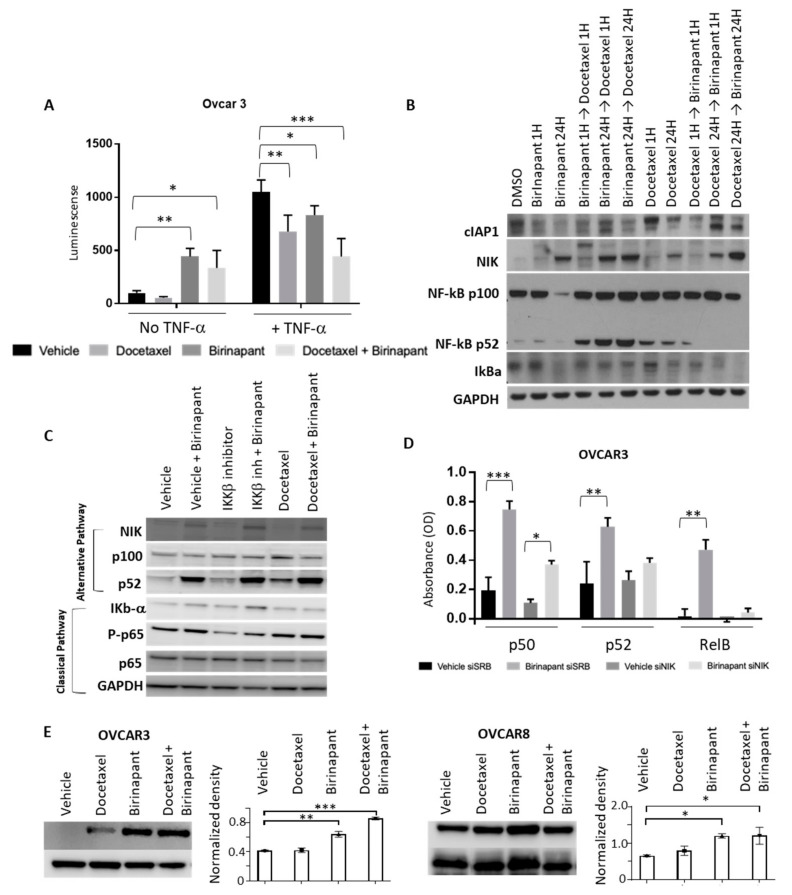
Birinapant suppresses the classical NF-kB pathway and enhances the alternative NF-kB pathway. (**A**) NF-kB signaling was assessed in OVCAR3 cells after establishing a reporter cell line stably transduced with a lentiviral vector containing luciferase under control of NF-kB consensus response element. Reporter cells were treated with single-agent (docetaxel 4, birinapant 400 nM) or combination therapy for 24 h, both with and without exogenous TNF-α (10 ng/mL). * *p* < 0.05; ** *p* < 0.001; *** *p* < 0.0001. (**B**,**C**) NF-kB alternative and classical pathway proteins were assessed by western blot to determine the effect of birinapant on NF-kB signaling. Cells were treated for 3 h with either single-agent (400 birinapant or 4 nM docetaxel) or concurrent combination therapy prior to western blot analysis. (**D**) Nuclear protein binding of p50, p52, and RelB to NF-kB consensus oligonucleotide was quantified using TransAm NF-kB assay, both with and without NIK knockdown to assess the input of NF-kB alternative pathway activation. Cells were treated with birinapant 400 nM for 1 h prior to analysis. Absorbance reflects density of colorimetric secondary antibody. * *p* < 0.01; ** *p* < 0.001; *** *p* < 0.0001. (**E**) Glu-tubulin, a marker of stable microtubules, was measured by western blot after exposure of OVCAR3 and OVCAR8 cells for 30 min to vehicle, docetaxel 4, birinapant 400, or docetaxel 4, plus birinapant 400 nM. Shown are representative images (left panels). Quantification of duplicate blots (right panels) was performed by densitometry with all values normalized to GAPDH using LI-COR software. * *p* = 0.05; ** *p* < 0.01; *** *p* < 0.001. Error bars in A, D, and E indicate standard error of the mean.

Because of the known role of cIAP1 in promoting classical NF-kB and suppressing alternative NF-kB signaling [26], we sought to determine how NF-kB signaling was affected by birinapant alone, docetaxel alone, or the combination. NF-kB luciferase reporter lines were established in OVCAR3 cell lines. Reporter cell lines were then treated with docetaxel or birinapant alone or in combination. Birinapant appeared to activate NF-kB signaling when added by itself (Figure 5A, left). Based on the NF-kB consensus sequence in the reporter construct, we were unable to distinguish between classical or alternative NF-kB pathways from these results. Exogenous TNF-α was added in order to specifically activate the classical pathway of NF-kB signaling since it would not affect the alternative pathway. In this way, we could assess the effect of each drug or the combination specifically on classical NF-kB signaling. As expected, in the presence of TNF-α, the NF-kB reporter was activated as demonstrated by an increase in relative luminescence. When docetaxel was added, NF-kB activation was reduced both with and without TNF-α. Birinapant activated the NF-kB reporter in the absence of TNF-α but attenuated the TNF-α induced rise in activity either alone or in combination with docetaxel (Figure 5A). These results suggest that birinapant may be activating alternative NF-kB signaling, but blocking classical NF-kB activity, consistent with its effect in depleting cIAP1.

NF-kB can signal through classical or alternative pathways. The classical pathway typically cascades through IkBa, IKKb, and RelA, while the alternative pathway activates through p100, IKKa, and RelB. Levels of NF-kB alternative or classical pathway proteins were assessed by western blot with simultaneous and sequential birinapant and docetaxel treatment (Figure 5B,C). Birinapant alone or in combination with docetaxel resulted in the stabilization of NF-kB inducing kinase (NIK) protein, consistent with activation of alternative NF-kB signaling in the absence of cIAP1. This effect on the alternative NF-kB pathway was related to birinapant, likely due to its mechanism of degrading cIAP1. The cIAP1 is known to destabilize NIK, and in the absence of cIAP1, we observed the NIK protein to be stabilized [27]. Docetaxel did not have an effect on NIK stability. Interestingly, the alternative NF-kB factor p52 accumulated when birinapant was dosed prior to or at the same time as docetaxel, suggesting alternative pathway activation, but pre-treatment with docetaxel prior to birinapant did not show increased p100 processing to p52, possibly suggesting a difference in protein trafficking caused by changes in tubulin dynamics. Classical pathway inhibition with IKKβ inhibitor blocked IkBα degradation, leading to its accumulation, and docetaxel had a similar effect with short (1-h) treatment.

We next evaluated the effect of birinapant on NF-kB DNA binding. Nuclear protein was harvested to comparatively quantify p50, p52, and RelB expression both in the presence and absence of birinapant. Knockdown of NIK was performed to further assess the effects on the alternative NF-kB pathway. Birinapant increased transcription factor binding to an oligonucleotide of the NF-kB consensus sequence, as detected by each factor-specific antibody (Figure 5D). NIK knockdown decreased the birinapant-induced DNA binding as noted by the attenuated increase in each transcription factor binding to the consensus oligonucleotide. Taken together, these results suggest that birinapant stimulates alternative NF-kB signaling which may contribute to the increase in TNF-α transcription. TNF-α can activate apoptosis and enhance cell death in the presence of docetaxel. In the absence of pro-survival classical NF-kB signaling, which is blocked by birinapant, the pro-apoptotic effect of both TNF-α and docetaxel are increased.

### 2.5. Birinapant Stabilizes Microtubules to Enhance Docetaxel Activity

Docetaxel is a member of the taxane family of chemotherapeutic agents and exerts its primary effects through microtubule stabilization, resulting in cell cycle arrest, inhibition of cellular protein transport, and ultimately apoptosis. Taxanes bind to preformed microtubules and therefore the magnitude of their cytotoxic effect depends on the pretreatment state of microtubules. Classical NF-kB activity is known to destabilize microtubules, and inhibition of IKKβ stabilizes microtubules [28]. We hypothesized that the ability of birinapant to block classical NF-kB signaling may be another mechanism of synergy with docetaxel. We sought to determine if pretreatment with birinapant affected the state of microtubules by measuring the level of detyrosinated α- tubulin (glu-tubulin) in OVCAR8 and OVCAR3 cells. Glu-tubulin is a marker of stable microtubules, with increasing levels indicative of a higher amount of preformed microtubules [29]. Glu tubulin levels were dramatically increased as measured by Western blot after exposure to birinapant alone and even further with combination birinapant and docetaxel (Figure 5E and Figure S3), indicating that birinapant induces microtubule stabilization, thereby improving docetaxel binding and providing another mechanism of synergy for the two agents.

### 2.6. Docetaxel Achieves Better Cancer Control in Combination with Birinapant In Vivo

**Figure 6:**
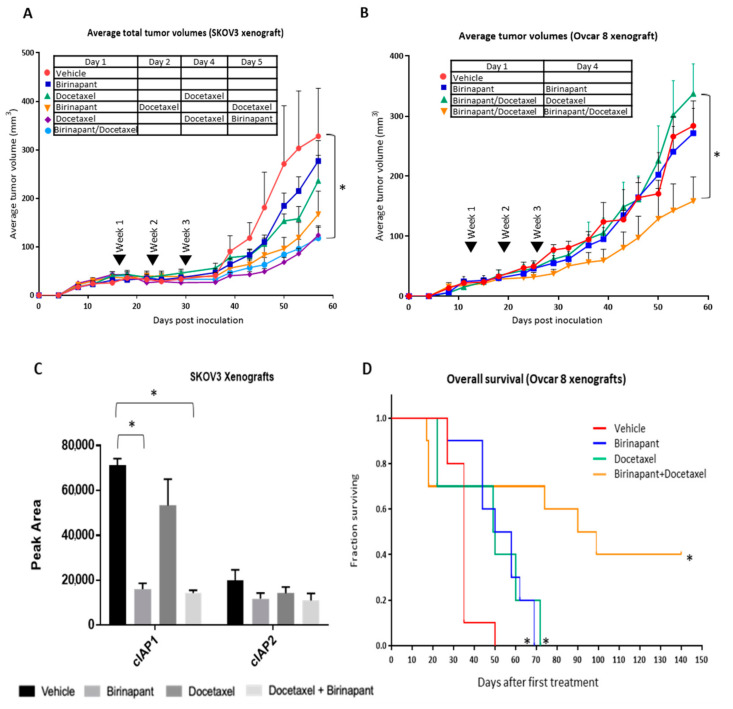
Docetaxel is synergistic with birinapant in vivo. (**A**) Athymic nude mice were inoculated subcutaneously with SKOV3 ovarian cancer cells. Mice were randomized into treatment groups after tumors achieved an average volume of 50–100 mm^3^. Average total tumor volumes over time were measured by calipers following 3 weeks of vehicle, single-agent, or combined intraperitoneal (IP) treatments of birinapant 15 and docetaxel 6 mg/kg. Treatments occurred simultaneously or sequentially, as shown in the inset table. SKOV3 xenografts were established subcutaneously. (**B**) In another subcutaneous model using OVCAR8 cells, the effect of dosing mice once weekly was compared to split dosing twice weekly, as indicated in the inset table. Measurements were carried out as in (**A**). (**C**) In a separate experiment, subcutaneous SKOV3 xenografts were harvested after one week of treatment. Protein was extracted and capillary western blot was used to quantify cIAP1 and cIAP2. * *p* < 0.0001. (**D**) OVCAR8 cells were inoculated intraperitoneal into athymic nude mice. Survival was monitored after receiving 3 weekly IP treatments of single or combined birinapant 15 and docetaxel 6 mg/kg. Log-rank test was performed to determine statistically significant differences in survival. * *p* < 0.001. Error bars in all panels indicate standard error of the mean.

We next sought to establish the effects of combining birinapant and docetaxel in mouse models of ovarian cancer. Both subcutaneous and intraperitoneal models were investigated. SKOV3 xenografts were established by subcutaneous injection into 6–8-week-old athymic female mice. Tumors were grown for 2 weeks, until they reached an average volume of 50–100 mm^3^, at which time mice were randomized in groups of 5 to treatment. Mice received 3 weekly intraperitoneal (IP) treatments of either single-agent or combined therapy with birinapant (15 mg/kg) and docetaxel (6 mg/kg) (Figure 6A). Body weights and tumor measurements were taken twice weekly for 8–10 weeks and subcutaneous tumor volume was calculated. The average tumor volume was lower for mice treated with single-agent birinapant or docetaxel compared to vehicle-treated mice, and lowest for mice receiving combined treatment. The order of administration did not affect tumor growth in this model. In another subcutaneous model using OVCAR8 cells, we tested the effect of dosing mice once weekly compared to split dosing twice weekly. The best tumor control was achieved with split twice weekly dosing of both birinapant and docetaxel (Figure 6B).

To confirm the target birinapant activity in vivo, cIAP1 and cIAP2 activity were quantified in protein extracts from tumors harvested after one week of treatment. cIAP1 and cIAP2 are direct targets of the SMAC mimetic birinapant. SMAC-mimetics have been shown to target these proteins in in vitro models. To quantify cIAP1 and cIAP2 activity in vivo, a simple western analysis was performed. Single-agent docetaxel had minimal activity against cIAP1 and cIAP2. However, cIAP1 was significantly decreased in the presence of birinapant, either alone or in combination with docetaxel, confirming on-target activity in vivo (Figure 6C).

Finally, overall survival was assessed using an orthotopic intraperitoneal model of OVCAR8. After 2 weeks of xenografts growth, mice were treated with the split dosing regimen found to be most effective in the subcutaneous model. Mice received 3 weekly IP treatments of single or combined birinapant (15 mg/kg) and docetaxel (6 mg/kg) (*n* = 10 mice per group). Statistically significant differences in overall survival were noted between the treatment groups (* *p* < 0.001). Overall survival was dramatically increased in mice treated with combination therapy, with a median survival of approximately 91 days. Mice treated with single-agent therapy had a median survival of approximately 51 days. As expected, mice treated with the vehicle had the worst overall survival, with a median survival of just 31 days (Figure 6D).

## 3. Discussion

The ability of ovarian cancer cells to evade apoptosis is thought to contribute to chemotherapeutic resistance, disease progression, and recurrence. Taxanes are initially an effective class of drugs for women with ovarian cancer, but resistance eventually develops with each successive line of chemotherapy. Median progression-free survival with docetaxel in third line is only approximately 3.6 months [30]. Birinapant was previously tested as a single agent in women with a median of 5 prior lines of therapy [10]. No responses were achieved, and no patient reached progression-free survival greater than 6 months. In the current study, we performed an unbiased high throughput screen to identify drugs that synergize to kill ovarian cancer cells. From this, we identified the combination of docetaxel with birinapant as highly effective and proceeded to investigate this combination in vitro and in vivo.

We uncovered two potential mechanisms underlying the synergy between docetaxel and birinapant. First, we measured cytokine secretion and found that the combination dramatically increased TNF-α production. Both TNF-α and TRAIL have been shown to augment apoptosis with SMAC mimetics [28,31,32,33,34]. SMAC mimetics themselves can increase TNF-α secretion in some cells [18]. Here, we found that docetaxel further increased TNF-α secretion in combination with birinapant. Interestingly, the largest increase was seen when docetaxel was administered before birinapant. It is possible that the pretreatment with docetaxel allowed the birinapant to sustain the rise in TNFa via persistent activation of alternative NF-kB signaling. The reverse order may counteract the rise in TNF due to the differential effect on p100 processing and p52 localization that occurred. We initially hypothesized that the order of administration would be important in vivo, but this was not what we observed in the animal experiments. The order of administration did not affect results in vivo, likely due to the different kinetics of TNFa in a live animal receiving multiple doses of the drugs, compared to the single dosing in a culture dish with no circulatory system or tumor microenvironment.

A recently published paper by Lalaoui et. al found a similar mechanism of action between docetaxel and birinapant in triple-negative breast cancer (TNBC). In that study, birinapant-induced cell-death in TNBC was augmented by the addition of docetaxel via the induction of TNF-α [35]. In addition to augmenting SMAC mimetic-induced apoptosis, TNF-α could have broader effects on the immune system by stimulating cytotoxic T cells to act against cancer cells [36]. Previous work supported testing birinapant in combination with immune checkpoint inhibitors [5,37,38]. Our new findings suggested that the addition of docetaxel could further sensitize cancer cells to treatment with SMAC mimetics and immune checkpoint inhibitors, which is currently under active investigation.

Tubulin stabilization was a second mechanism of cooperation between birinapant and docetaxel. Inhibition of IKKβ in the classical NF-kB pathway can stabilize tubulin, measured by the increase in glu-tubulin protein, to give greater access to taxane binding [29,39]. We showed that birinapant and docetaxel blocked the TNF-α induced increase in NF-kB luciferase reporter activity. TNF-α is known to signal solely through classical NF-kB pathway [40]. The signaling complex at the cytoplasmic tail of TNFR1 involves TRAF2 which recruits cIAP1, leading to activation of IKKβ to phosphorylate IkBa. Phosphorylated IkBa is subsequently degraded in the proteasome which releases classical NF-kB transcription factors RelA and p50 [41]. As a SMAC mimetic, birinapant causes degradation of cIAP1, thus blocking this signaling node upstream of IKKβ. Inhibition of signaling in this axis of NF-kB stabilizes tubulin, making it more accessible for binding by docetaxel, and facilitating the mechanism of action of this chemotherapy agent. In addition, the SMAC mimetic activity turns off NF-kB signals for pro-survival and proliferation, further enabling apoptosis and thus enhancing the synergy between docetaxel and birinapant.

Both birinapant and docetaxel have limited efficacy when used as single agents for women with recurrent ovarian cancer. The global unbiased matrix drug screen that we used in this investigation is a powerful discovery mechanism to identify synergistic combinations that may improve outcomes for women with this incurable disease. An understanding of the cellular mechanisms impacted by chemotherapeutic agents and potential synergistic combinations that may exist will allow for the optimization of chemotherapeutic regimens and tumor response.

## 4. Materials and Methods

### 4.1. Cell Culture

Ovarian cancer cell lines IGROV1, OVCAR3, OVCAR4, OVCAR5, OVCAR8, and SKOV3 were obtained from the NCI-Frederick Developmental Therapeutics Program tumor/cell line repository (Frederick, MD, USA). HEY-A8 cell line was a gift from Dr. Elise Kohn, National Cancer Institute. Cell line 1A9 is a clone from A2780, and PTX10 is a paclitaxel-resistant clone from A2780, all of which were gifts from Dr. Tito Fojo, National Cancer Institute. PEO-1 cells were provided by Dr. Craig J. Thomas at the National Center for Advancing Translational Sciences (Bethesda, MD, USA). Cell cultures were grown in RPMI (Roswell Park Memorial Institute) medium supplemented with 10% FBS (Fetal Bovine Serum), 1% penicillin/streptomycin, and maintained at 37 °C in an incubator under a 5% CO_2_ atmosphere. Serous ovarian cell lines ACI-8, ACI-23, ACI-27, ACI-35, and ACI-54 were gifted from Dr. John Risinger at Georgia Memorial Hospital. They were cultured in DMEM/F12 medium supplemented with 10% FBS and 1% penicillin/streptomycin. Serous ovarian line CaOV3 was purchased from ATCC (American Type Culture Collection) and was cultured in RPMI supplemented with 10% FBS and 1% penicillin/streptomycin. Endometrioid ovarian line CRL-11731 was purchased from ATCC was cultured in ATCC Complete medium supplemented with 15% FBS and 1% penicillin/streptomycin. Endometrioid ovarian cancer CRL-10303 was purchased from ATCC and grown in DMEM/F12 medium supplemented with 10% FBS and 1% penicillin/streptomycin.

### 4.2. Drug Screen

A high-throughput screening matrix identified distinct drug classes as synergistic with birinapant. Drugs were applied to 1584-well plates using an automated, acoustic dispensation system as previously described [18,20]. PEO-1 ovarian cancer cell lines were then added to the wells and incubated for 72 h. Cell viability was determined by Cell-titer Glo reagent, and apoptosis was measured by Caspase3-Glo assay. The initial screen was a 6 × 6 evaluation of birinapant (Chemitek, Indianapolis, IN, USA) vs. a collection of 1912 approved and investigational drugs. The 6 × 6 matrix included 5-fold drug dilutions with a DMSO control. A total of 72 agents were advanced into confirmatory 10 × 10 experiments based on combination effects and mechanistic interest and translational potential [23]. All 10 × 10 experiments were plated in a similar manner, with 9-fold drug dilutions and a DMSO control. All screening data is publicly available at https://tripod.nih.gov/matrix-client/.

### 4.3. XTT Viability Assay

Further in vitro testing verified synergistic activity between birinapant and docetaxel. Cancer cell lines ACI-8, ACI-23, ACI-27, ACI-35, ACI-54, CaOV3, CRL-11731, CRL-10303, OVCAR3, OVCAR5, OVCAR8, HEYA8, A2780, SKOV3, IGROV1, and PTX10 were seeded in 96-well plates at a density of 2–3 × 10^3^ cells/well and incubated for 24 h prior to drug application. In the case of dual drug treatment, both drugs were added simultaneously. Seventy-two hours after treatment, XTT-PMS (2,3-bis-(2-methoxy-4-nitro-5-sulfophenyl)-2H-tetrazolium-5-carboxanilide, N-methyl dibenzopyrazine methyl sulfate) dye [42] was added to each well, incubated for 1.5 h, and then OD 450 nm was measured to determine cell viability (SpectraMax ID3 microplate reader, Molecular Devices, San Jose, CA, USA).

### 4.4. Caspase Activity Assay

Caspase 3/7, Caspase 8, and Caspase 9 activity was measured in OVCAR3 cells lines using Caspase-Glo luminescence assays (G8091, G8201, G8211, Promega, Madison, WI, USA) in accordance with the manufacturer’s specifications after exposing cells to docetaxel 4 nM (#S1148, Selleckchem, Houston, TX, USA) alone and in combination with birinapant 400 nM for 24 h. All drug exposures occurred with 10 ng/mL of TNF-α and without TNF-α. Activity data were normalized to viable cell number, measured in an identical plate by the XTT assay as described.

### 4.5. NF-kB Reporter Assay

OVCAR3 cells lines were selected given their high canonical NF-kB activity. Cells were transduced with Cignal Lenti NF-kB reporter (Luc) to measure NF-kB activation (catalog #336851, Qiagen, Germantown, MD, USA) [11]. Reporter cells were plated at a density of 2000 cells/50 uL per well in a 96-well plate and incubated overnight before changing to starvation medium (0.5% FBS RPMI). The following day, cells were exposed to docetaxel 4 nM alone or in combination with birinapant 400 nM for 24 h. All drug exposures occurred with 10 ng/mL of TNF-α and without TNF-α. IKK-2 Inhibitor IV (catalog #01484, Calbiochem, Burlington, MA, USA) at a dose of 2 μM, a known antagonist of the classical NF-kB pathway, was used as a control. Quantitative luminescence was measured by Luciferase Assay (catalog #E1501, Promega, Madison, WI, USA) using a SpectraMax ID3 plate reader and normalized to a cellular viability assay using XTT-PMS dye.

### 4.6. TransAM Assay

OVCAR3 cells were seeded in 96-well plates at a density of 2–3 × 10^3^ cells/well and incubated for 24 h. Cells were transfected with either non-targeting or NIK-targeting siRNA at a final concentration of 5 nM using DharmaFECT I Transfection Reagent (GE Dharmacon, Horizon Discovery, Boyertown, PA, USA) as previously described [43]. RPMI medium was exchanged and cells were treated with birinapant 400 nM for 1 h. Nuclear extracts were made from treated cells using Lysis Buffer AM2 (catalog #37512, Active Motif, Carlsbad, CA, USA). Concentrations were estimated with the BCA Protein Assay Kit (Thermo Fisher Scientific, Waltham, MA, USA). A TransAM NF-kB Activation Assay was performed per the manufacturer’s protocol (catalog #43296, Active Motif, Carlsbad, CA, USA). Raji nuclear extract was used as a positive control. The following primary antibodies from the TransAM NF-kB Activation Assay Kit were used: p50, p52, and RelB. The developing solution was added to the wells and absorbance was read on a SpectraMax ID3 plate reader at 450 nm.

### 4.7. Western Blot

OVCAR3 cells were plated in 6-well plates at a density of 1 × 10^6^ cells/well and left overnight. Cells were then treated with single or combination therapy (birinapant 400 nM, docetaxel 4 nM) for 3 h and then total protein was extracted using RIPA (Radio-Immuno-Precipitation Assay) buffer (catalog #24948, Santa Cruz Biotechnololgy, Santa Cruz, CA, USA) in accordance with the manufacturer’s protocol. Western blot analysis was then performed using the NuPAGE SystemTM (Thermo Fisher Scientific, Waltham, MA, USA). Concentrations were estimated with the BCA Protein Assay Kit (Thermo Fisher Scientific, Waltham, MA, USA). The following primary antibodies were used: IkBa (9242 s), NIK (4994 s), p100/p52 (4882 s), *p*-p65 (3039 s) (Cell Signaling Technology, Danvers, MA, USA). To assess if birinapant affects microtubule stability, OVCAR8 and OVCAR3 cells were plated in 6-well plates at a density of 5 × 10^5^ cells/well. Two wells were then treated with vehicle, docetaxel 4, birinapant 400, or docetaxel 4 nM plus birinapant 400 nM for 30 min prior to total protein extraction using RIPA buffer. Concentrations were estimated with the BCA Protein Assay Kit (Thermo Fisher Scientific, Waltham, MA, USA). The following primary antibodies from Abcam (Cambridge, MA, USA) were used: GAPDH (ab9484) and detyrosinated alpha tubulin (ab48389). Protein signal quantitation was done using Image Studio v 5.2.5 (Li-Cor Biotechnology, Lincoln, NE, USA). CaOV3 cells were seeded in T25 flasks at a density of 1 × 10^6^ cells and incubated overnight. Individual T25 flasks were then treated with DMSO, birinapant 200, docetaxel 2.5 nM, or both in accordance with the following schedule: Birinapant alone for 1 h, birinapant alone for 24 h, birinapant for 1 h followed by docetaxel for 1 h, birinapant for 24 h followed by docetaxel for 1 h, birinapant for 24 h followed by docetaxel for 24 h, docetaxel alone or 1 h, docetaxel alone for 24 h, docetaxel for 1 h followed by birinapant for 1 h, docetaxel for 24 h followed by birinapant for 1 h, docetaxel for 24 h followed by birinapant for 24 h. Cells were then harvested and western blot performed. The following antibodies were used: cleaved caspase-3 (Cell Signaling #9664), IAP1 (R&D #AF8181), IAP2 (R&D #AF8171), cleaved PARP (Cell Signaling #9541), Caspase 8 (Thermo Scientific #MA1-41280), RIP1K (Cell Signaling #3493), XIAP (Cell Signaling #2042), BID (Cell Signaling #2002), Bak (Cell Signaling #3814), Bcl-2 (Cell Signaling #2872), SMAC (Cell Signaling #2954), Bcl-xL (Cell Signaling #2762), Bim (Cell Signaling #2819), Phospho-Bcl-2 (Thr56) (Cell Signaling #2875), Bax (Cell Signaling #2774), and Caspase 9 (Cell Signaling #9502).

### 4.8. Cytokine Assay

Secreted cytokine levels of IL-1ß, IL-6, IL-8, and TNF-α were measured in culture supernatants using the Mesoscale multiplex assay after treating OVCAR3 and OVCAR8 cells overnight with docetaxel 4 nM alone and in combination with birinapant 400 nM per the manufacturer’s protocol (MesoScale Discovery, Rockville, MD, USA). To determine the impact of treatment order and/or duration of treatment on cytokine secretion, CaOV3 cells were treated with birinapant 200 and docetaxel 2.5 nM using the following time courses: Birinapant alone for 1 h, birinapant alone for 24 h, birinapant for 1 h followed by docetaxel for 1 h, birinapant for 24 h followed by docetaxel for 1 h, birinapant for 24 h followed by docetaxel for 24 h, docetaxel alone or 1 h, docetaxel alone for 24 h, docetaxel for 1 h followed by birinapant for 1 h, docetaxel for 24 h followed by birinapant for 1 h, docetaxel for 24 h followed by birinapant for 24 h. IL-6, IL-8, and TNF-α secretion was then measured in culture supernatants using the Mesoscale multiplex assay per the manufacturer’s protocol (MesoScale Discovery, Rockville, MD, USA).

### 4.9. In Vivo Survival Studies

Next, 1–2 × 10^6^ cells of SKOV3 and OVCAR8 cancer lines were counted and prepared as suspensions in 0.5 mL PBS for subcutaneous (flank) injections into 6–8-week-old athymic nu/nu female mice. Tumors were grown for two weeks before the mice were randomized into treatment groups. Mice then received intraperitoneal (IP) treatment with placebo, docetaxel 6, birinapant 15 mg/kg, or combination docetaxel plus birinapant. Body weights and tumor measurements were taken twice weekly for 8–10 weeks or as required by humane endpoints. Subcutaneous tumor volumes were calculated according to the formula:

$$V\;=\;1/2\left(\mathrm{length}\;\times {\;\mathrm{width}}^{2}\right).$$

For survival experiments, 1–2 × 10^6^ OVCAR8 cells were injected IP into 6–8-week-old athymic nude mice and allowed to grow for 2 weeks, before IP treatments with drugs as described. Mice were followed until euthanasia endpoints and scored for overall survival. Animal care was provided in accordance to procedures in the Guide for the Care and Use of Laboratory Animals. Experiments were carried out according to protocol MOB-008 approved by the National Cancer Institute (NCI) Animal Care and Use Committee in accordance with NIH Manual Chapter 3040-2, Animal Care and Use in the Intramural Program, and the Animal Welfare Act of the United States.

### 4.10. Simple Western

Simple western is an automated capillary based isoelectric focusing (IEF) immunoassay system (ProteinSimple, San Jose, CA, USA). SKOV3 xenografts were established and tissue was obtained from mice after 1 week of single drug or combined drug treatment. Lysates were produced with M-Per buffer containing phosphatase and protease inhibitors in accordance with the manufacturer’s protocol. Lysates were mixed with ampholyte premix and fluorescently labeled pI standards before loading into the NanoPro1000 system for analysis. Iso-electric focusing was performed in capillaries filled with a mixture of cell lysate (~40 ng protein), 1× fluorescent pI standard ladder, and 1× premix ampholyte. Data were averaged from 4 xenograft measurements per group ± standard deviation.

### 4.11. RNAi Experiments

Cells were transfected with siRNAs using DharmaFECT 1 Transfection Reagent per standard manufacture procedure (Qiagen, Germantown, MD, USA) and drugs were added 2 days later. siRNAs used were as follows: ON-TARGET plus Non-targeting Pool (D-001810-10; Horizon Discovery, Boyertown, PA, USA), ON-TARGET plus siRNA against human TNF-α, SP3 RELA, and NFkB2 (L-010546, L-023096, L-003533, and L-003918, respectively; Horizon Discovery) and FlexiTube siRNA against DR5 (Hs TNFRSF10B 2 FlexiTube siRNA Qiagen).

### 4.12. Quantitative Real-Time PCR

Cultured cells were exposed to birinapant (400 nM) and/or docetaxel (4 nM) for 18 h. cDNA was synthesized from total RNA using Superscript II RT (Invitrogen, Carlsbad, CA, USA). PCR was performed using the SYBR PCR Master Mix (Applied Biosystems, Foster City, CA, USA) on an ABI 7500 thermal cycler. B2 M expression was used as an internal control to normalize between samples. Primer sequences are commercially available from Applied Biosystems (Foster City, CA, USA) as follows: TNF-α (catalog# Hs00174128_m1), TRAIL (catalog# Hs00921974_m1), TNFR1 (catalog# Hs01042313_m1), and DR5 (catalog# Hs00366278_m1)

### 4.13. Statistical Analysis

All in vitro experiments were conducted in duplicate or triplicate of each experimental condition as noted above. Results were analyzed for statistically significant differences using two-tailed *t*-tests (2 groups) or ANOVA multiple comparison tests (3 or more groups) in GraphPad Prism version 8.0 for Windows (GraphPad Software, San Diego, CA, USA, www.graphpad.com). Quantification of western blots was performed by densitometry using LI-COR Image Studio Lite version 5.2 (LI-COR Biotechnology, Lincoln, NE, USA). Overall survival was estimated using the Kaplan–Meier method, with differences between treatment groups evaluated using a long-rank test in GraphPad Prism version 8.0 for Windows; *p*-values < 0.05 were considered statistically significant.

## 5. Conclusions

Therapeutic options for women with recurrent ovarian cancer are limited. We used a high-throughput approach to identify synergistic drug combinations that could be used in the clinic. While the drugs may have weak anti-cancer effects when used as single agents, we identified powerful activity with the combination of birinapant and docetaxel in treating ovarian cancer in vitro and in a mouse model of recurrent disease. Ongoing studies will further optimize this combination mechanistically, and a clinical trial is under development.

## Supplementary Materials

The following are available online at https://www.mdpi.com/2072-6694/12/12/3784/s1, Figure S1: (A) Changes in apoptosis proteins measured in SKOV3 after treatment with either single-agent or combination birinapant 200 nM and docetaxel 2.5 nM. (B) Changes in apoptosis proteins measured in OVCAR 4 after treatment with either single-agent or combination birinapant 200 nM and docetaxel 2.5 Nm, Figure S2: Original western blot images of Figure 3B, Figure S3: Original western blot images of Figure 5E, Figure S4: Original western blot images of Figure S1A,B, Table S1: Combination data for birinapant vs. all 1912 screened compounds as completed in 6 × 6 combination experiments in the HGSOC (High grade serous ovarian cancer) cell line PEO-1, Table S2: Combination data for birinapant-vs.-all 72 agents that were advanced into confirmatory 10 × 10 combination experiments in the HGSOC cell line PEO-1.
