# Supplementary material for: Matrix Drug Screen Identifies Synergistic Drug Combinations to Augment SMAC Mimetic Activity in Ovarian Cancer
Source: Cancers (Basel). 2020 Dec 15;12(12):3784. doi: 10.3390/cancers12123784 (PMC7765376; doi:10.3390/cancers12123784)
Supplement: Supplementary file 1 [file cancers-12-03784-s001.zip › cancers-986572-Supplementary materials/cancers-986572-suppl-final.docx]

Supplementary Materials: Matrix Drug Screen Identifies Synergistic Drug Combinations to Augment SMAC Mimetic Activity in Ovarian Cancer

Anne M. Noonan, Amanda Cousins, David Anderson, Kristen P. Zeligs, Kristen Bunch,
Lidia Hernandez, Yusuke Shibuya, Ian S. Goldlust, Rajarshi Guha, Marc Ferrer,
Craig J. Thomas, Christina M. Annunziata


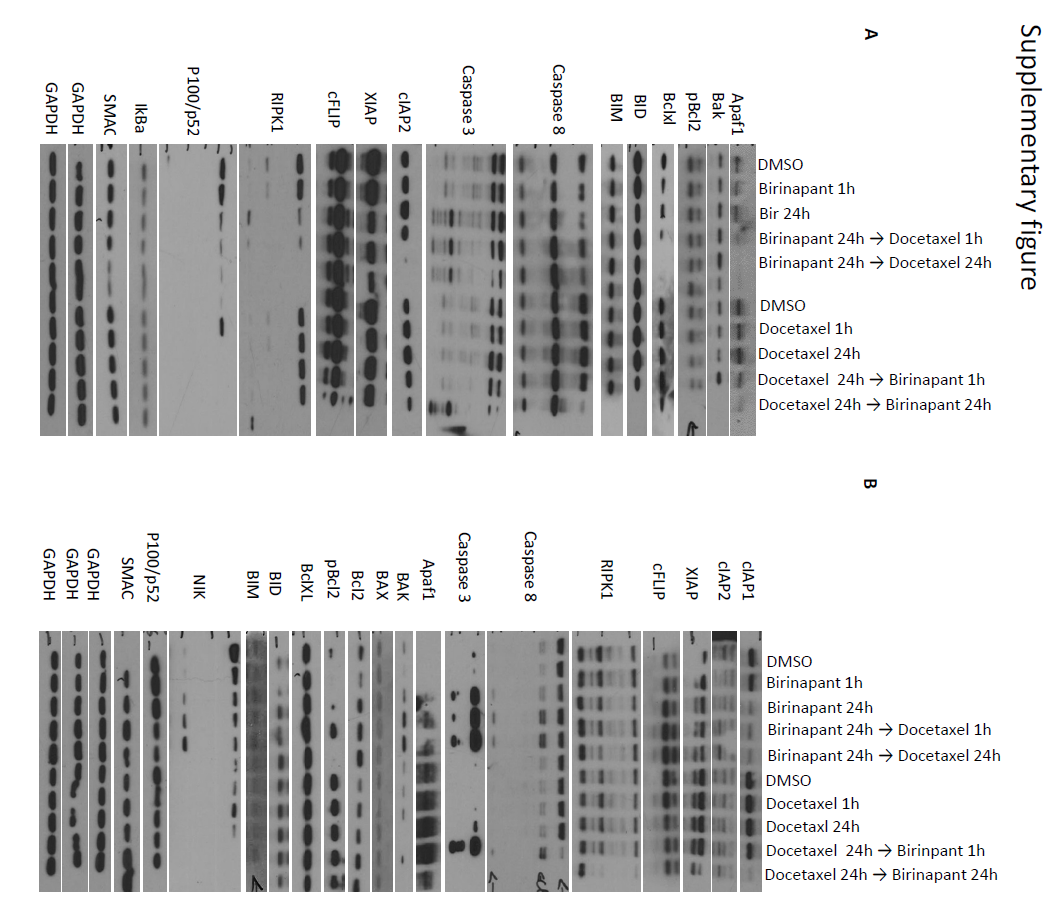


**Figure S1.** (**A**) Changes in apoptosis proteins measured in SKOV3 after treatment with either single-agent or combination birinapant 200 nM and docetaxel 2.5 nM. (**B**) Changes in apoptosis proteins measured in OVCAR 4 after treatment with either single-agent or combination birinapant 200 nM and docetaxel 2.5 nM.


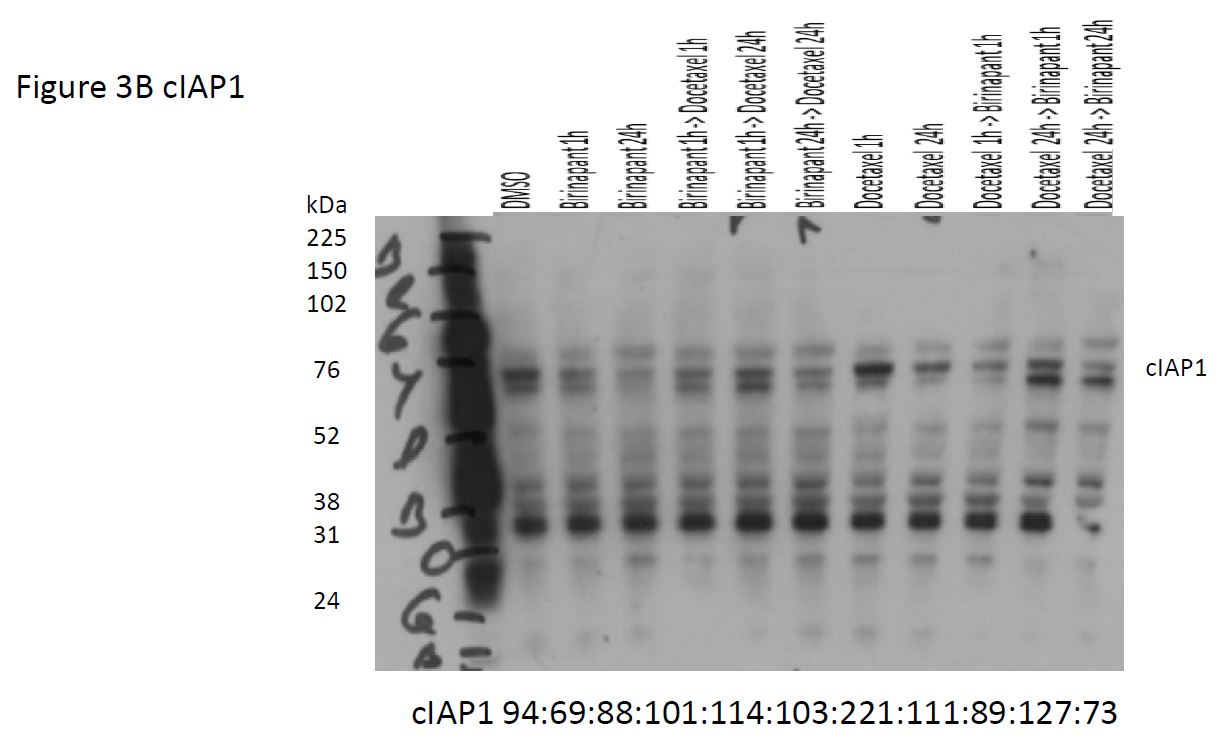


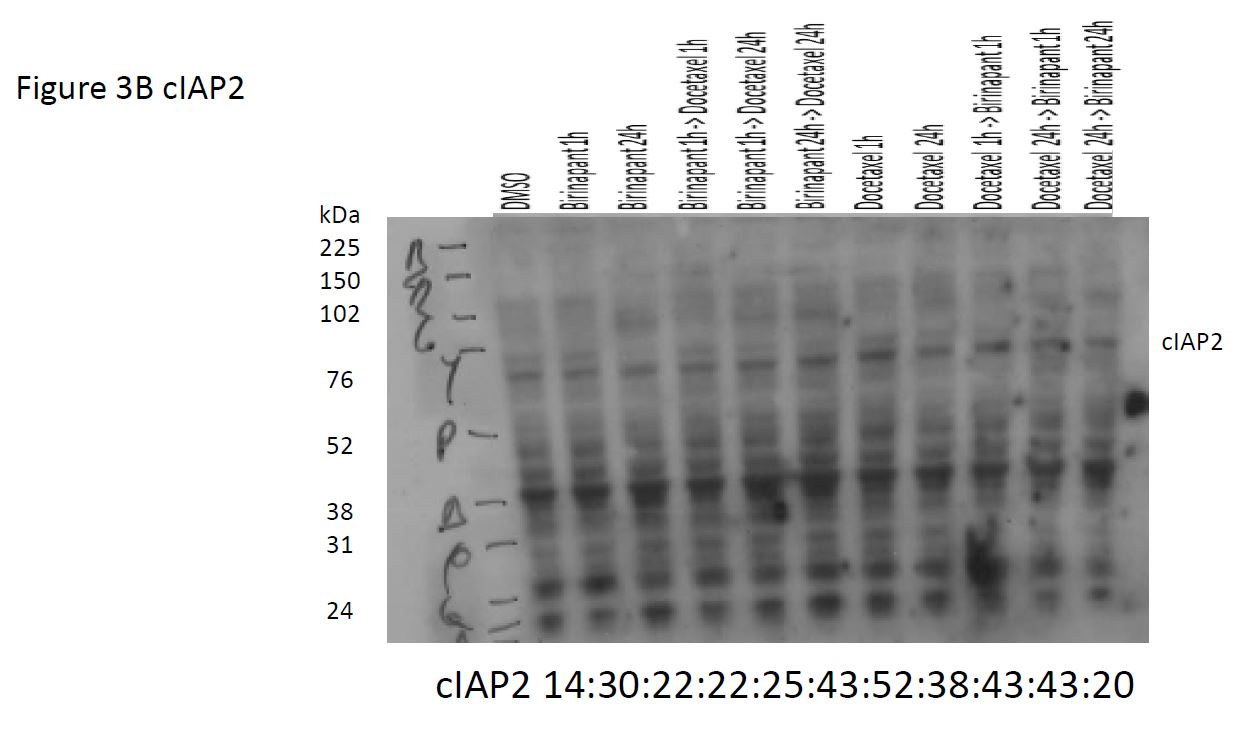


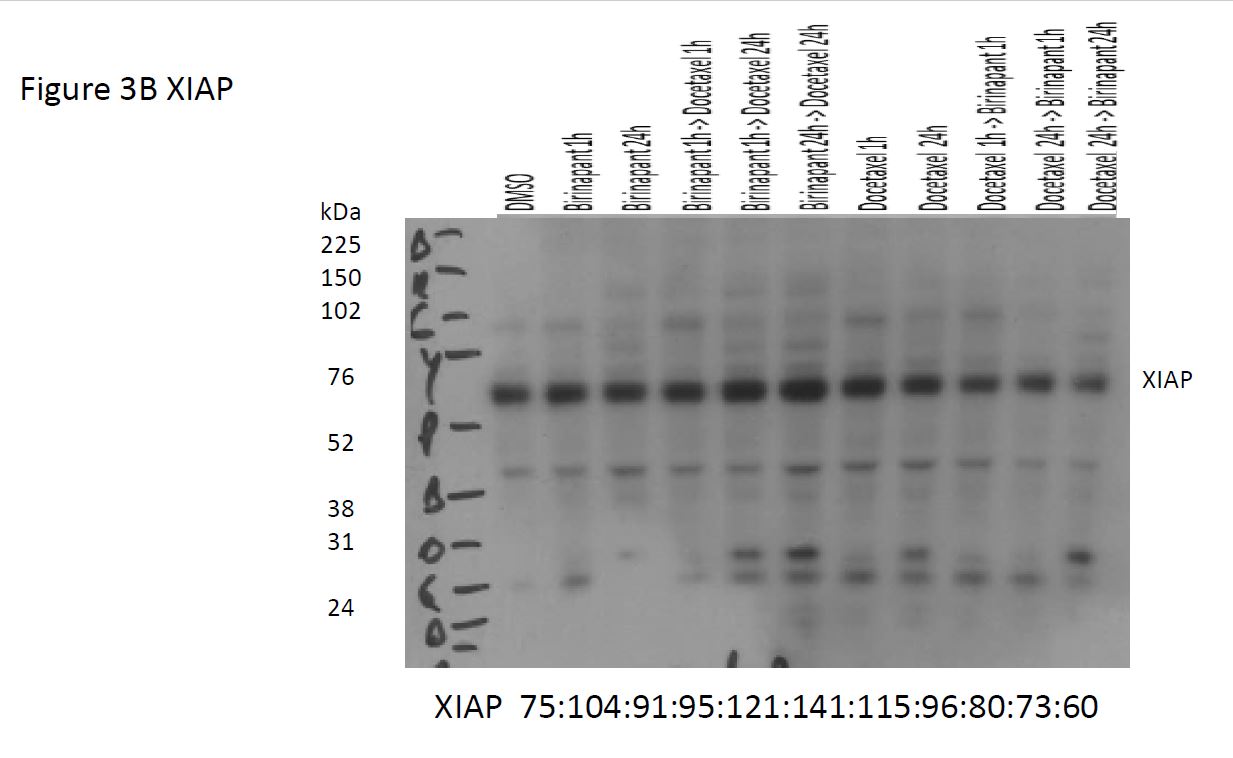


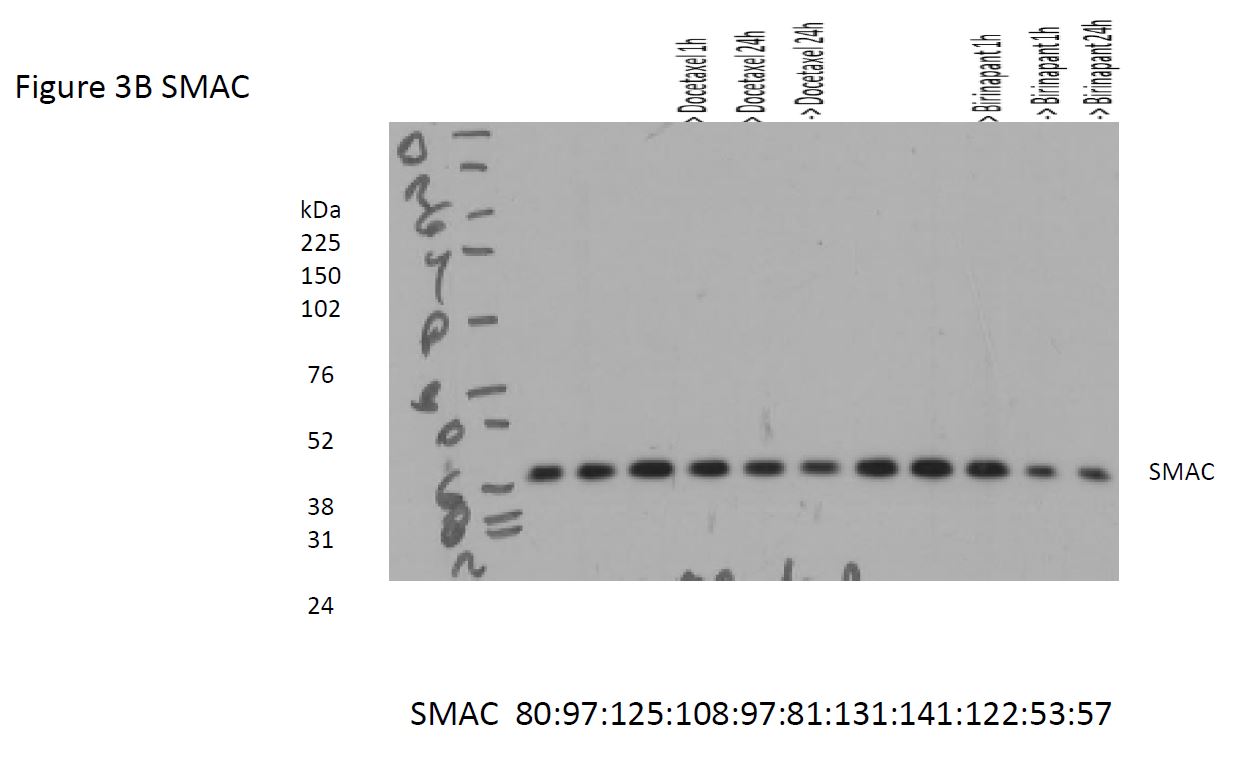


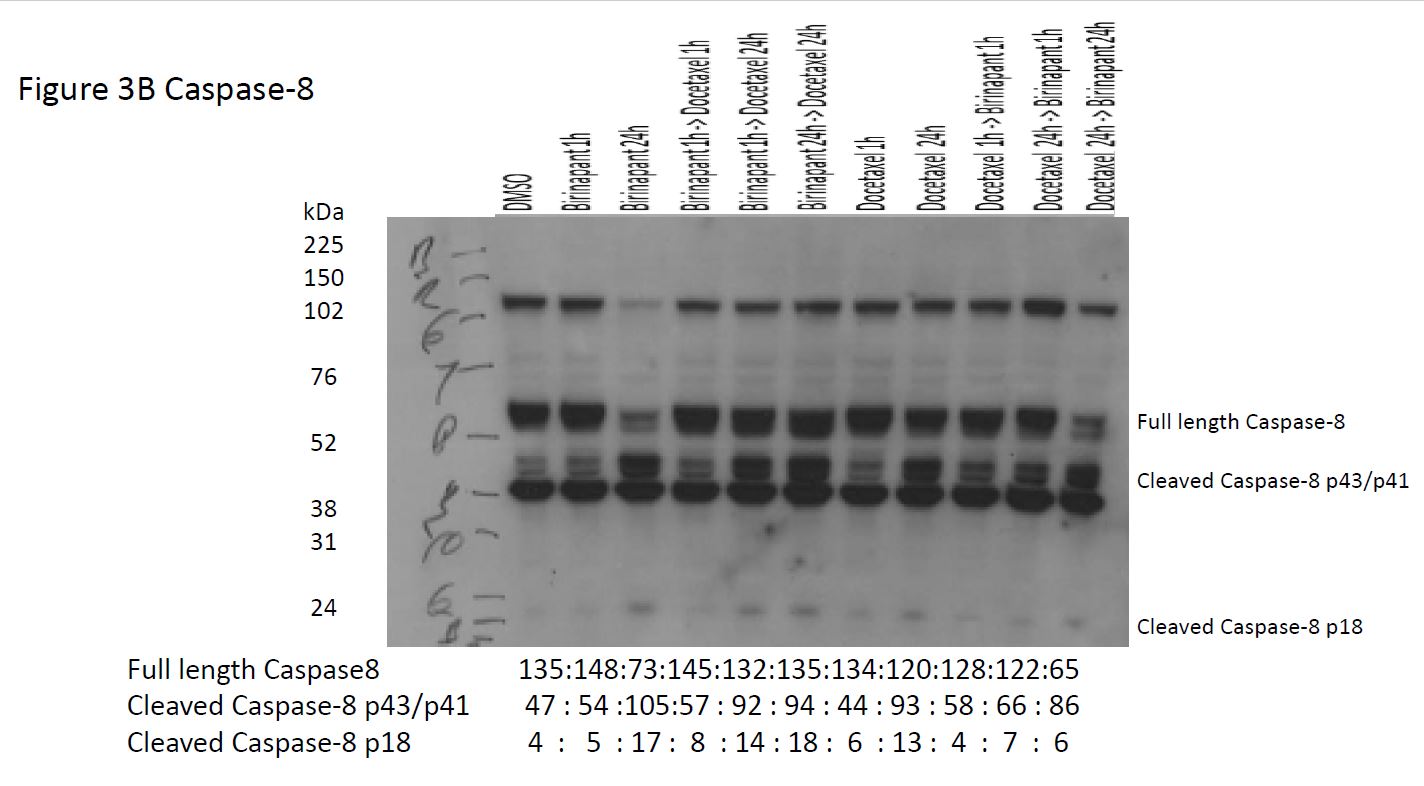


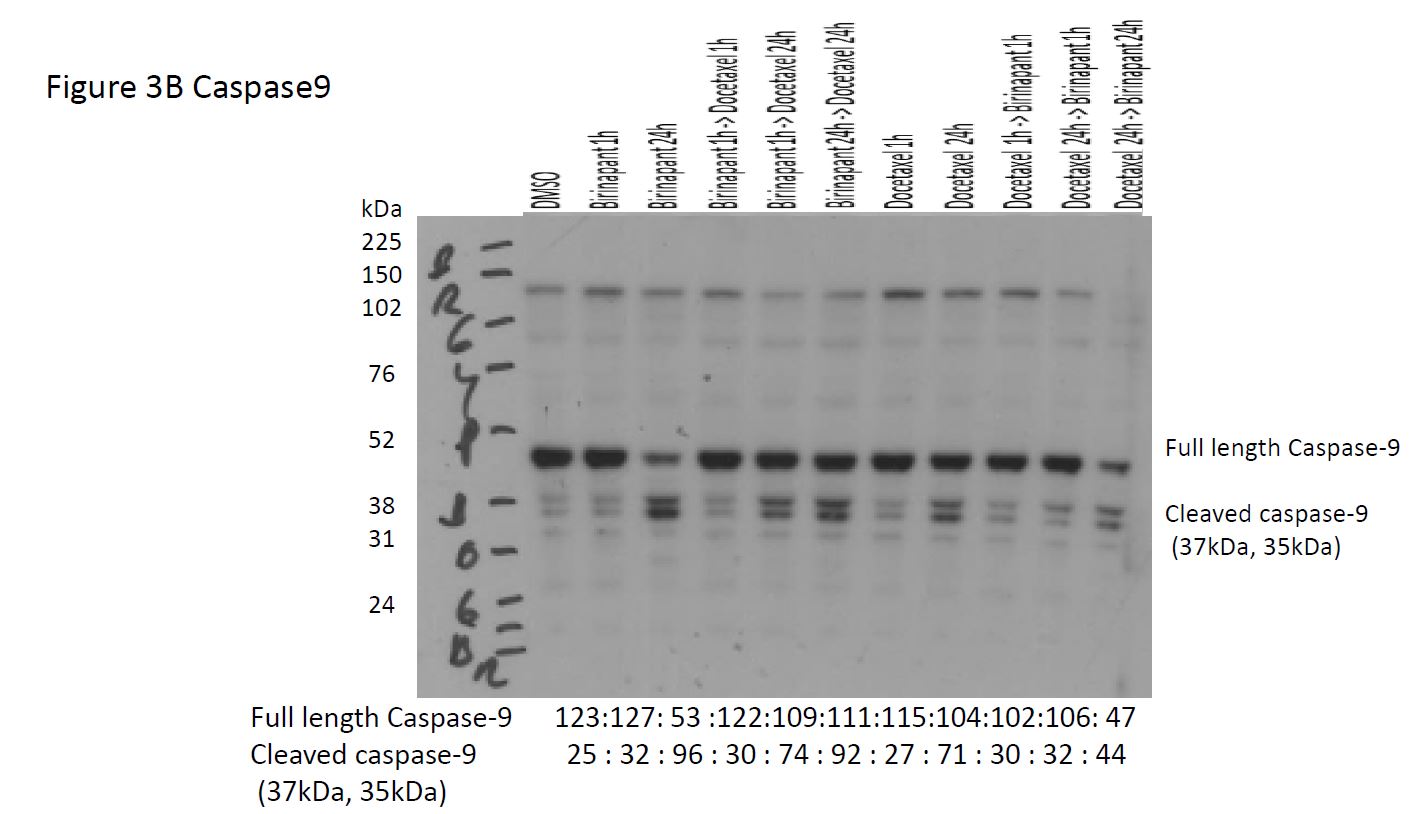


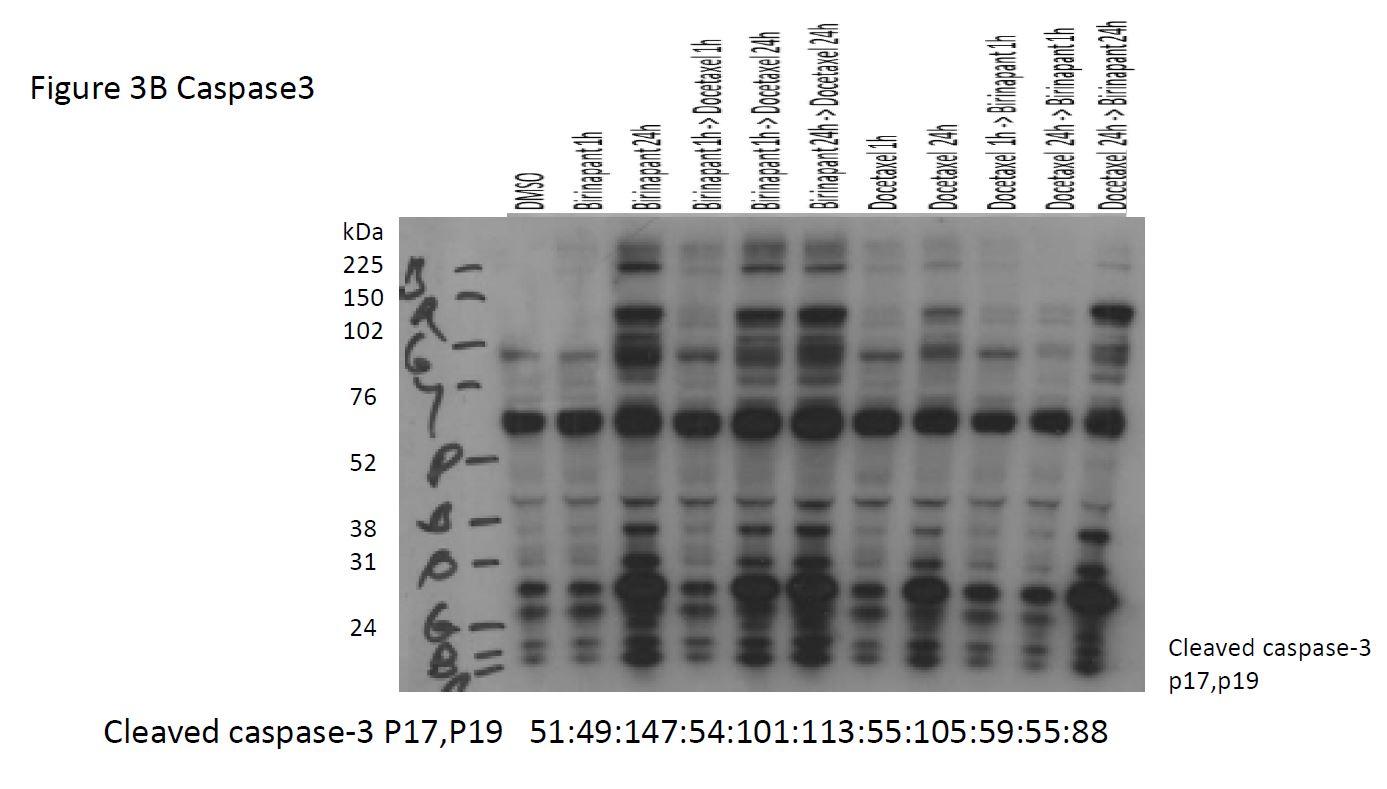


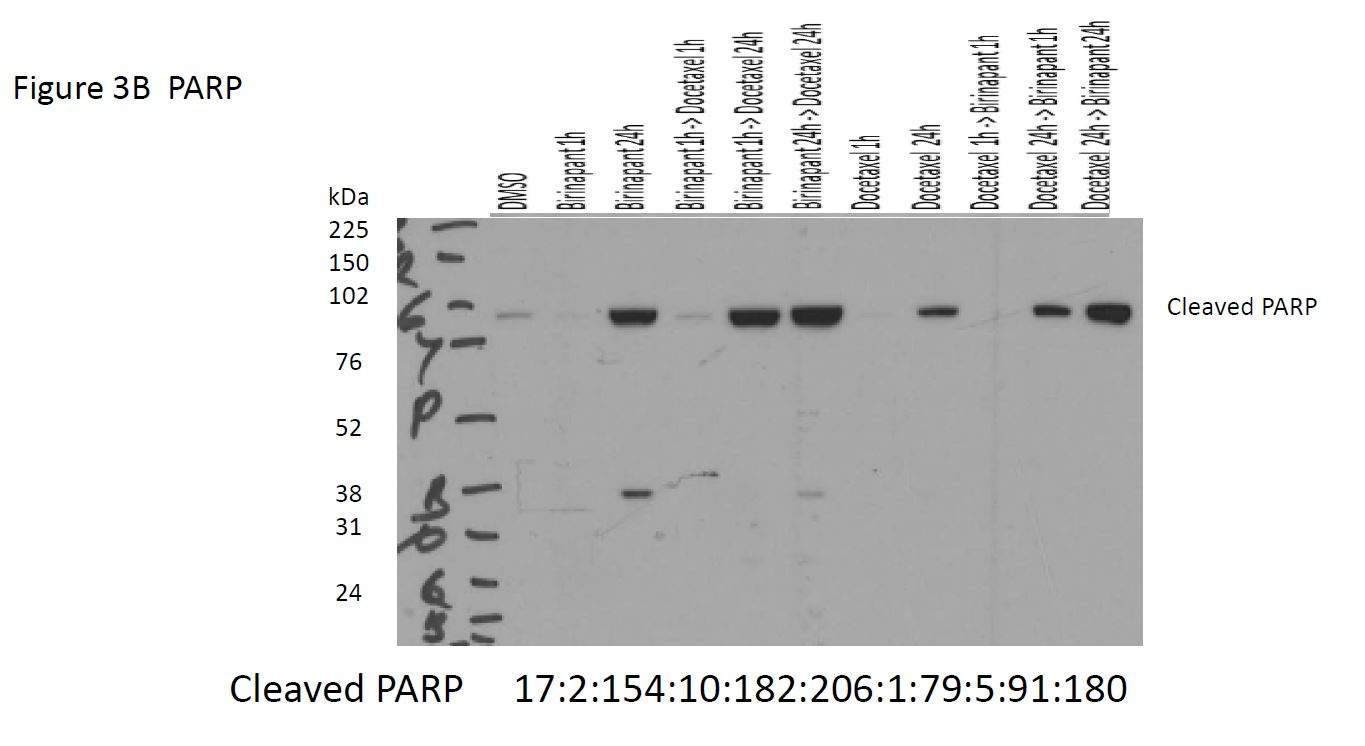


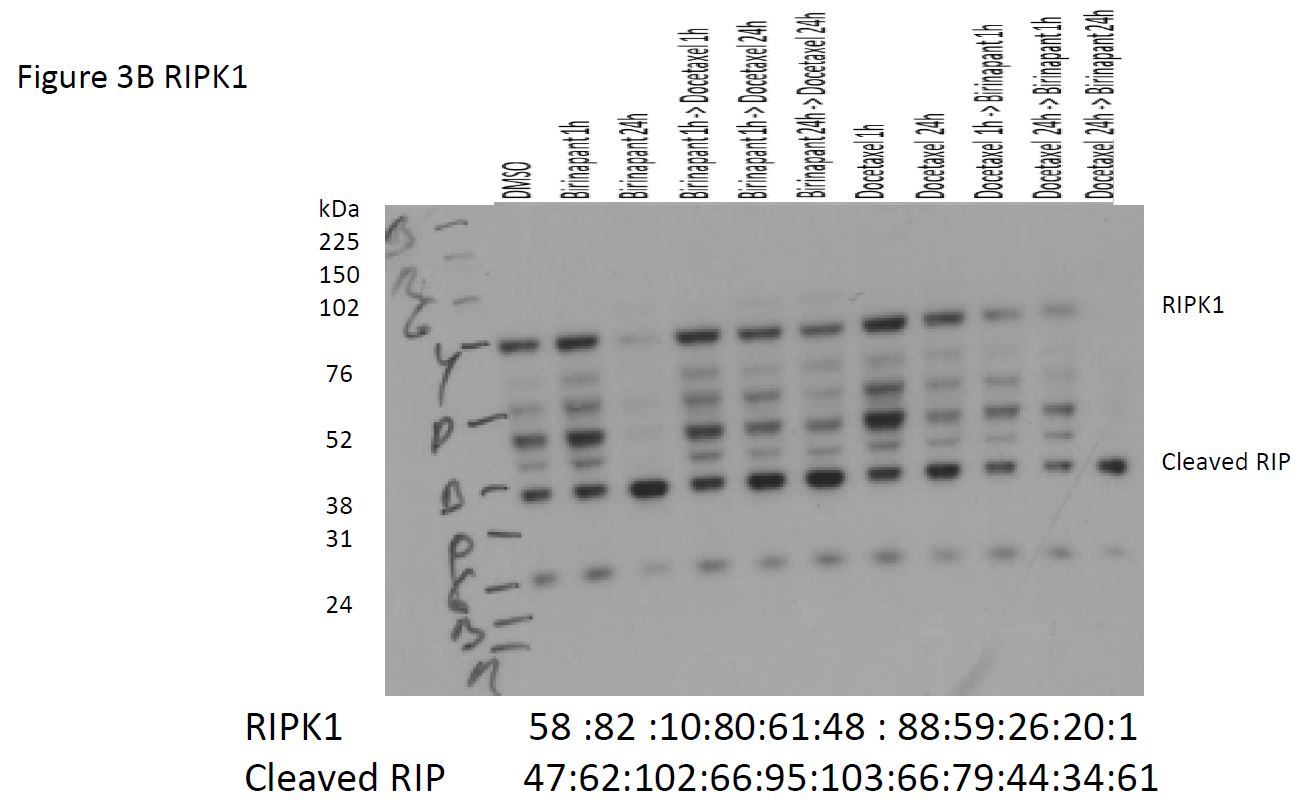


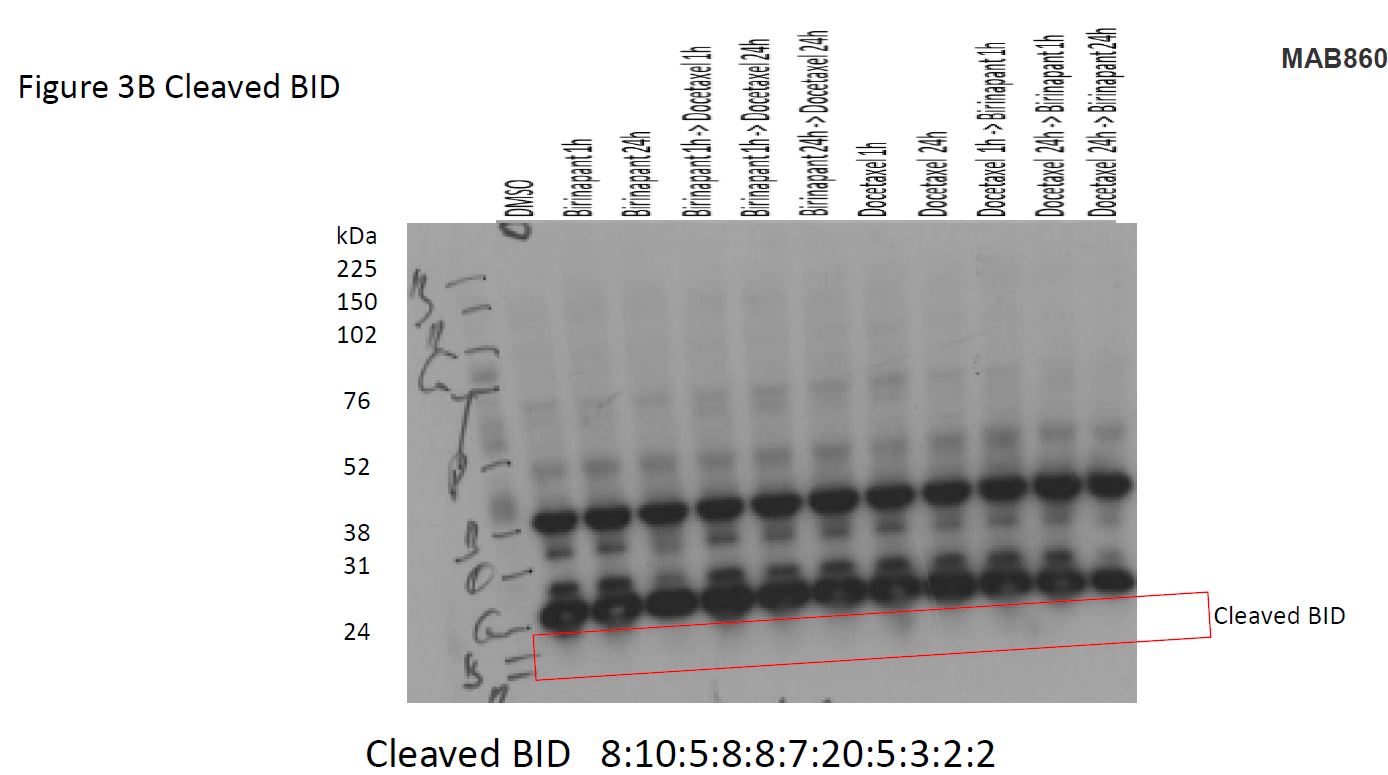


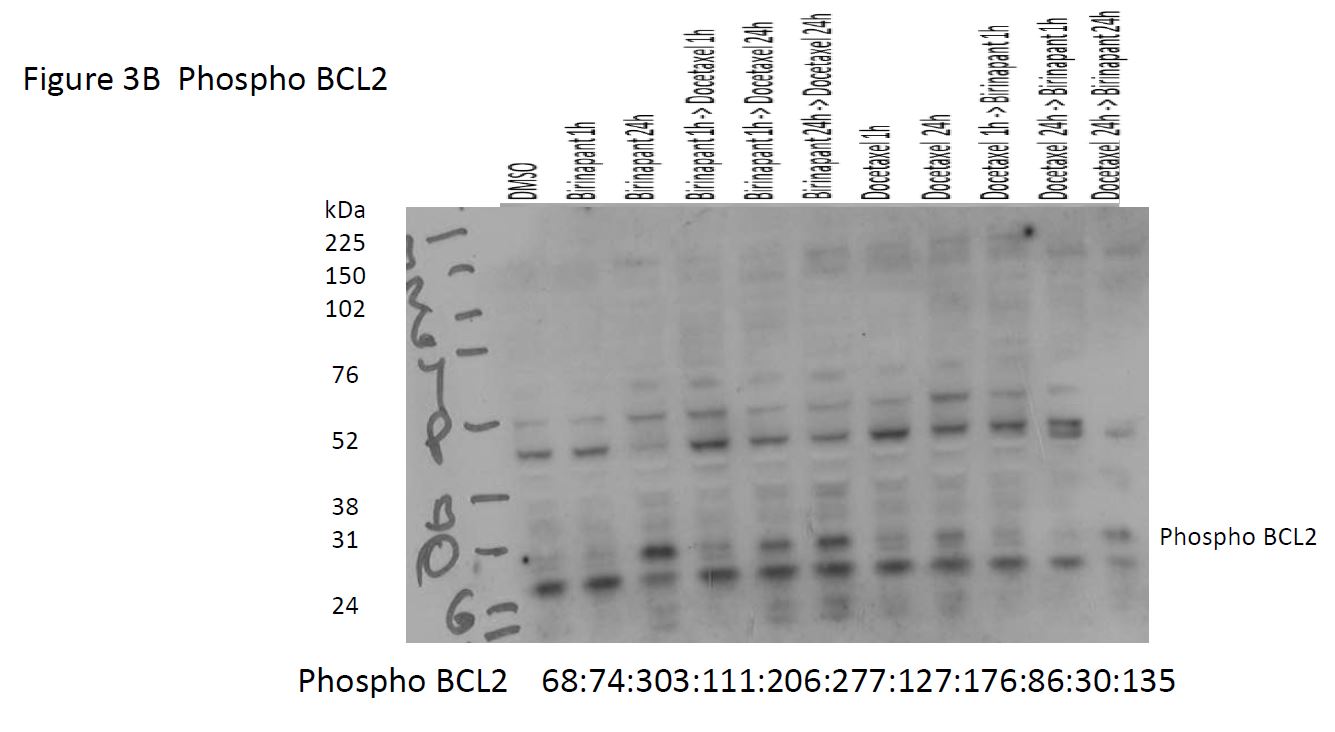


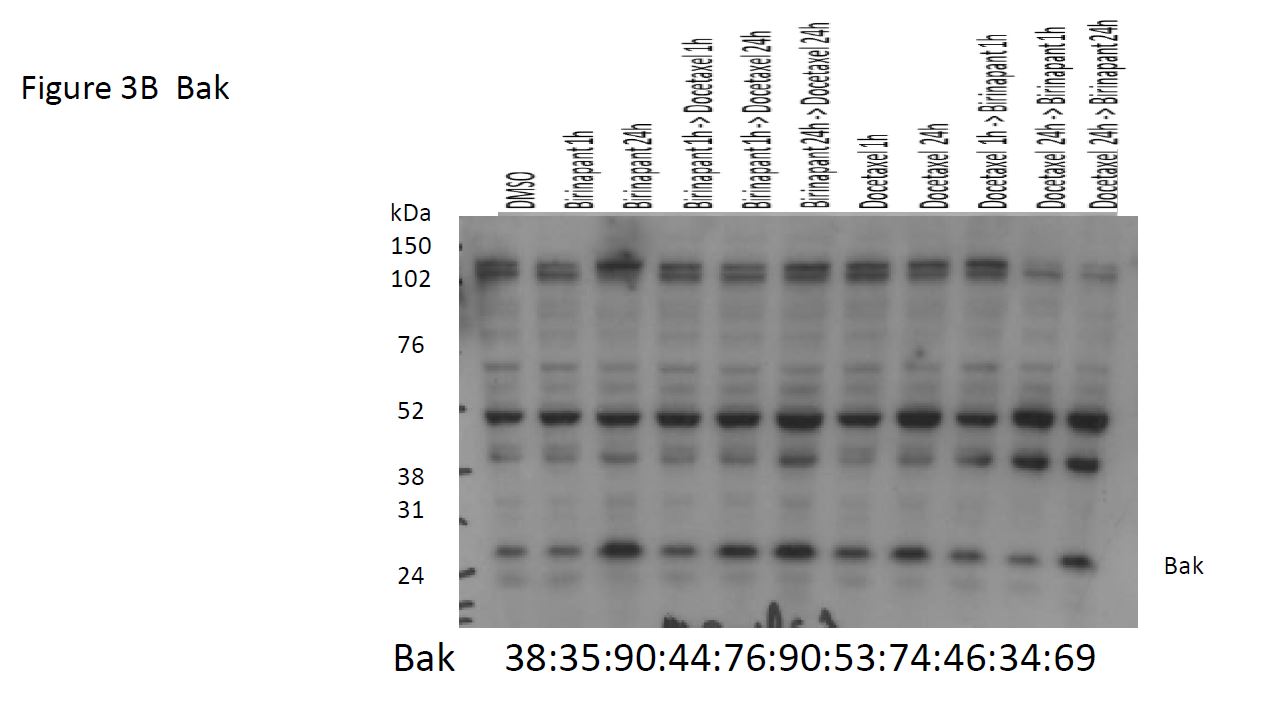


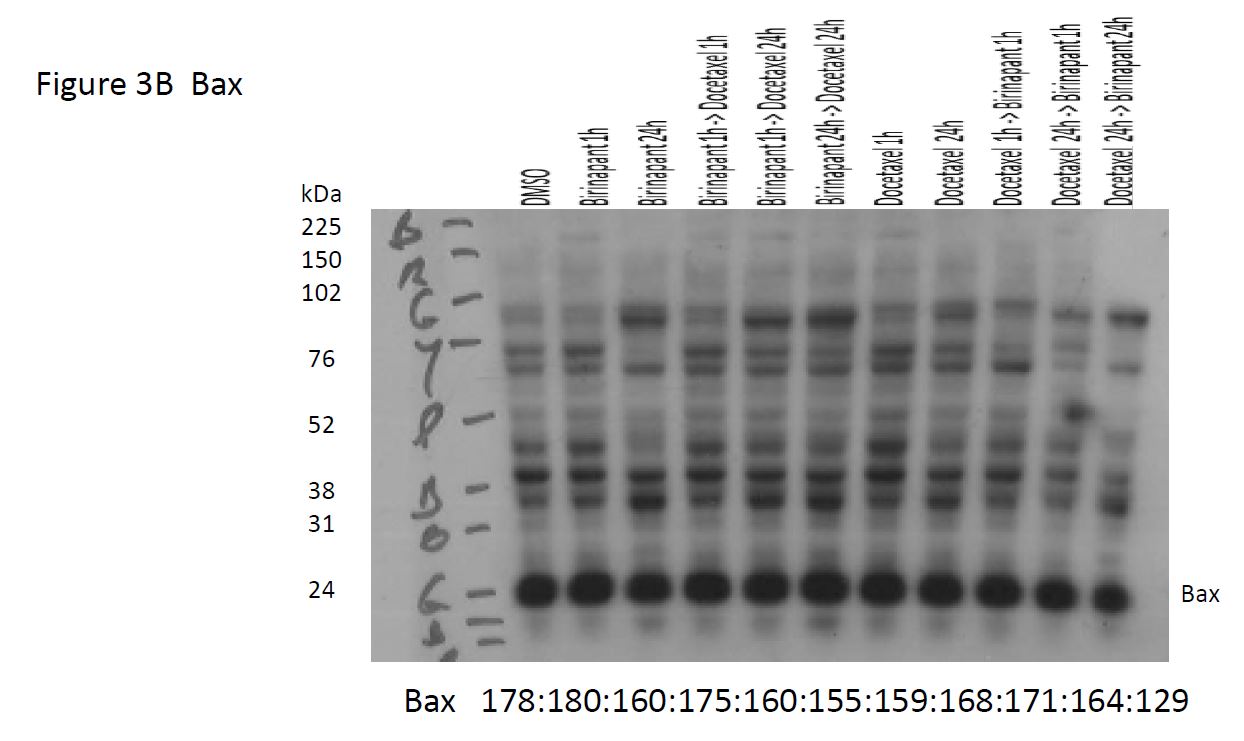


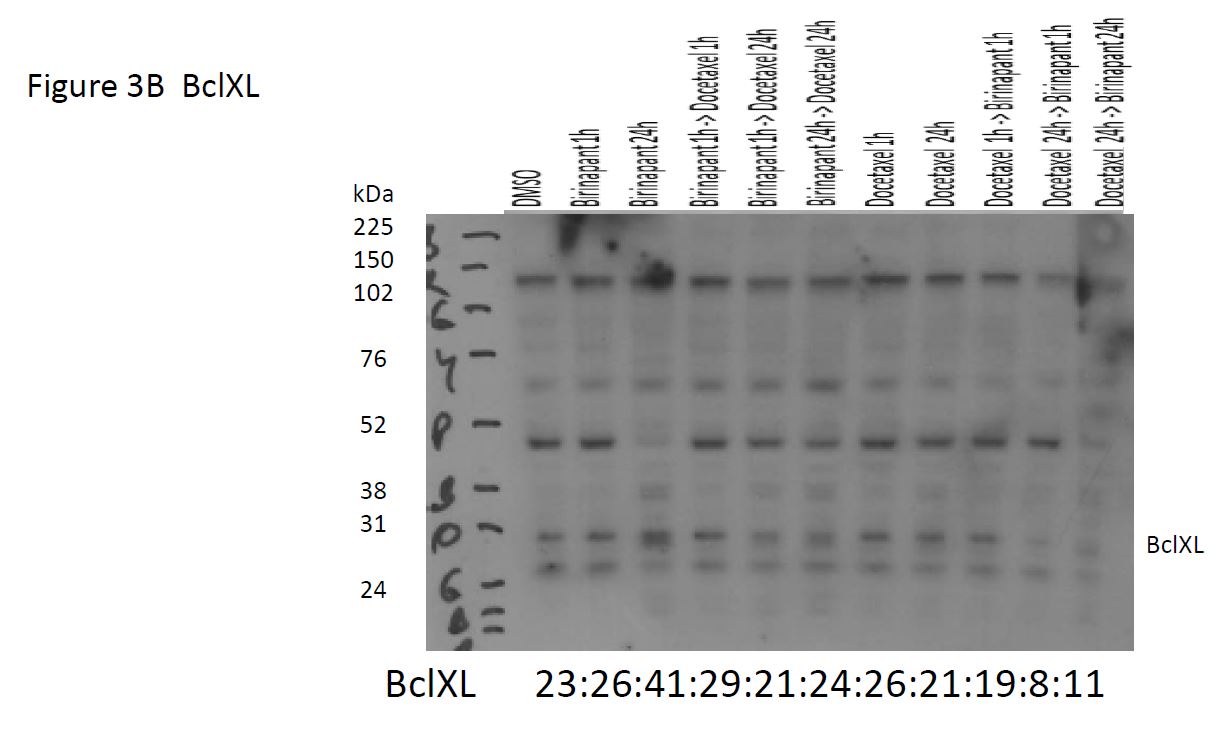


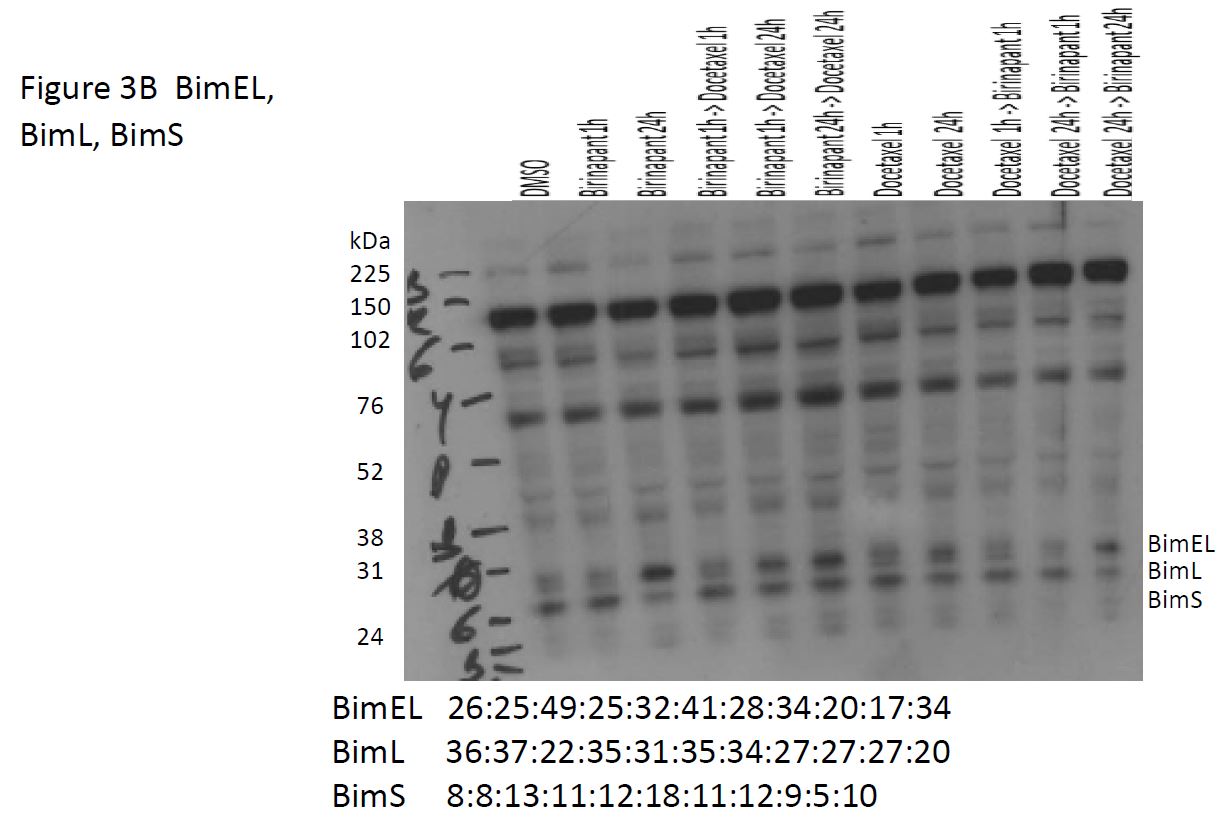


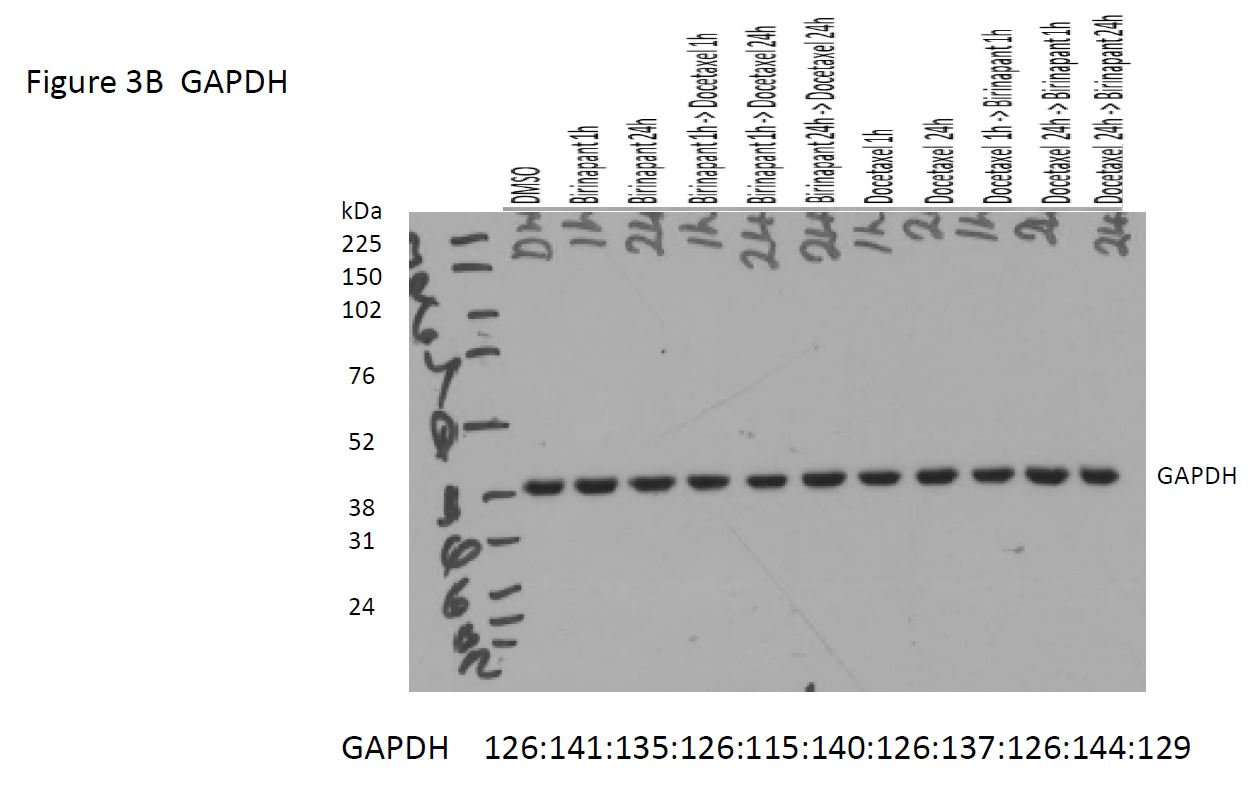


**Figure S2.** original western blot images of Figure 3B.


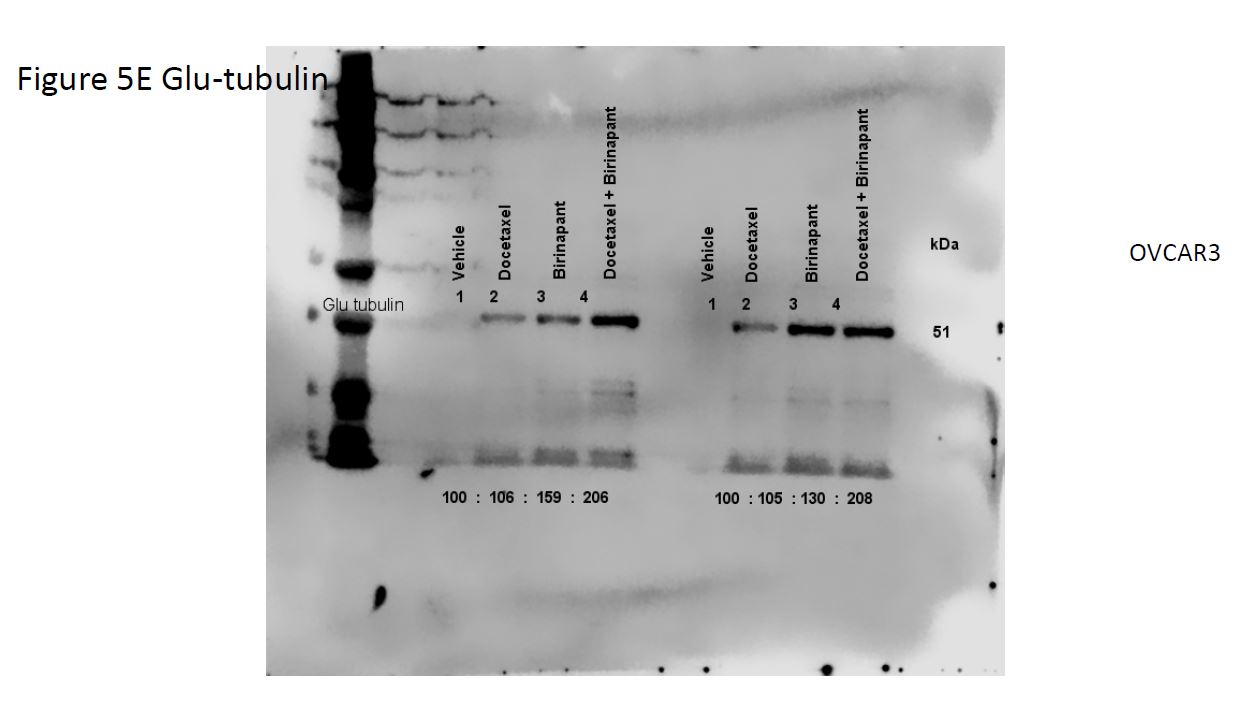


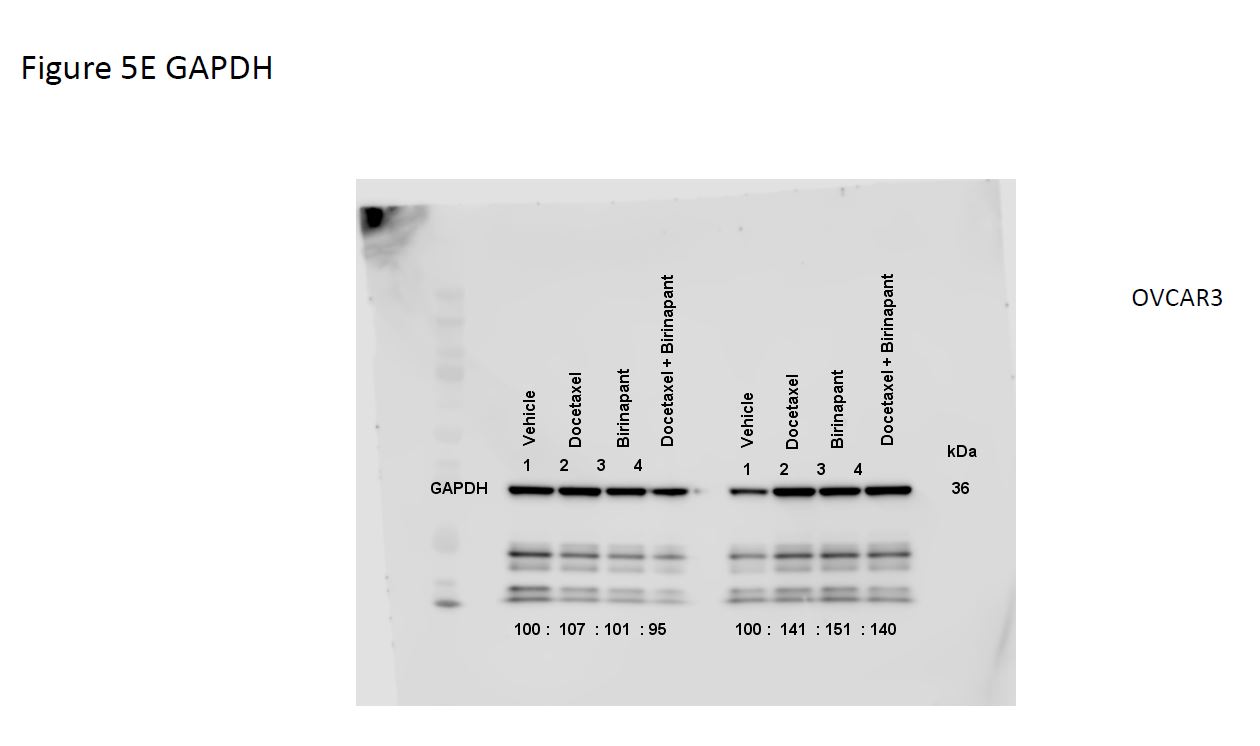


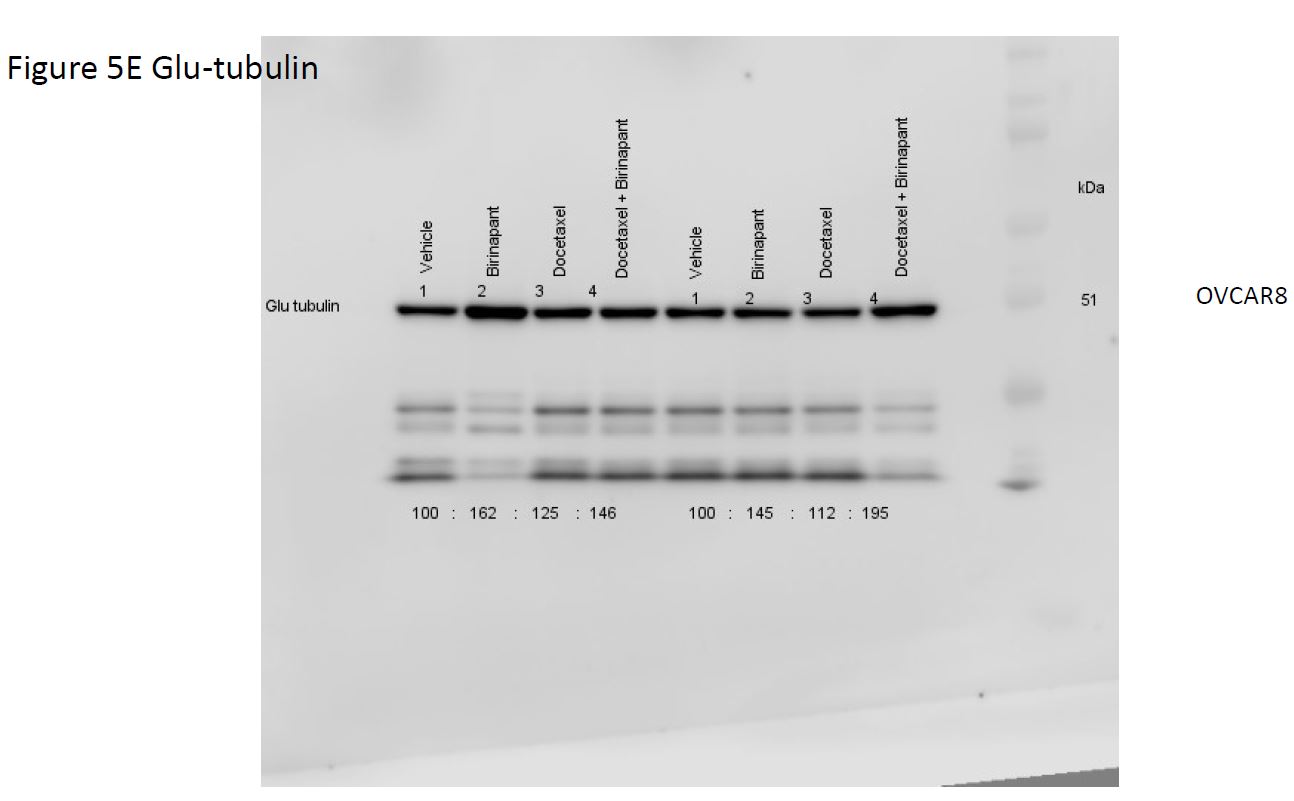


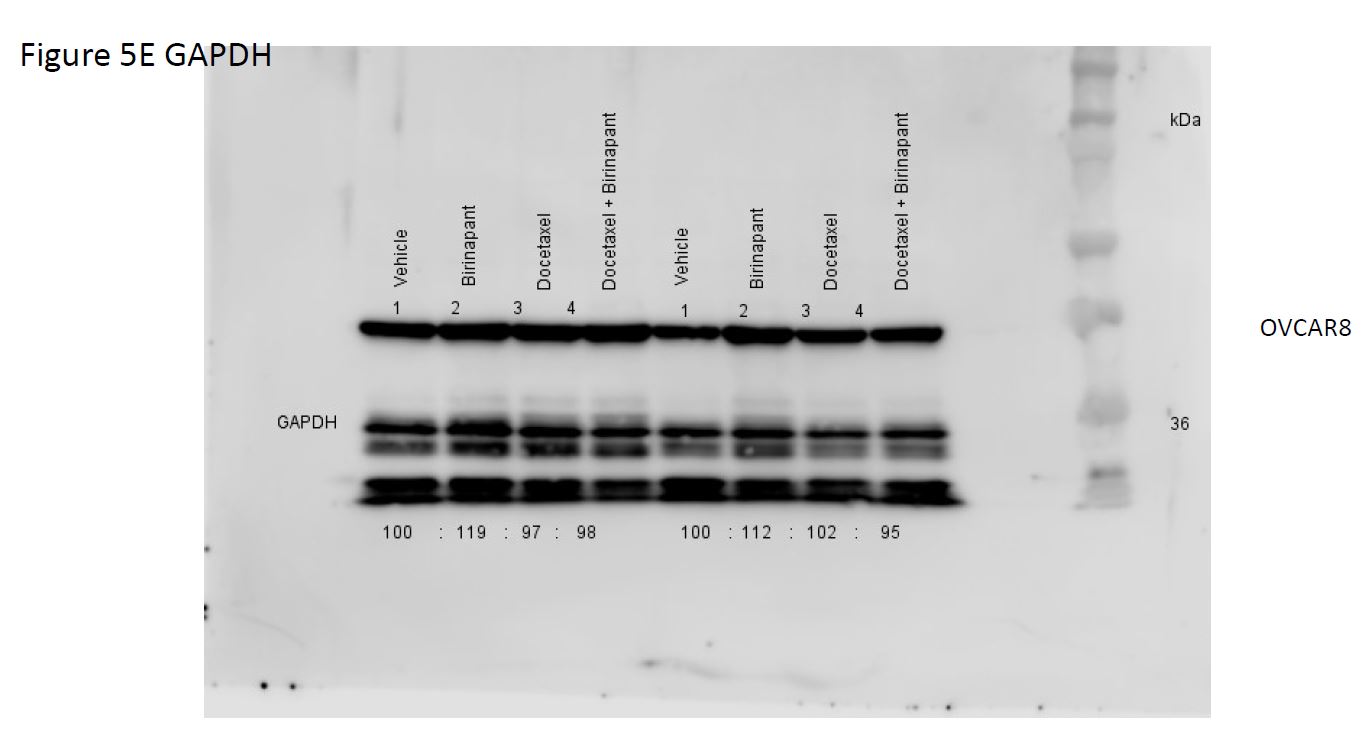


**Figure S3.** original western blot figures of Figure 5E.


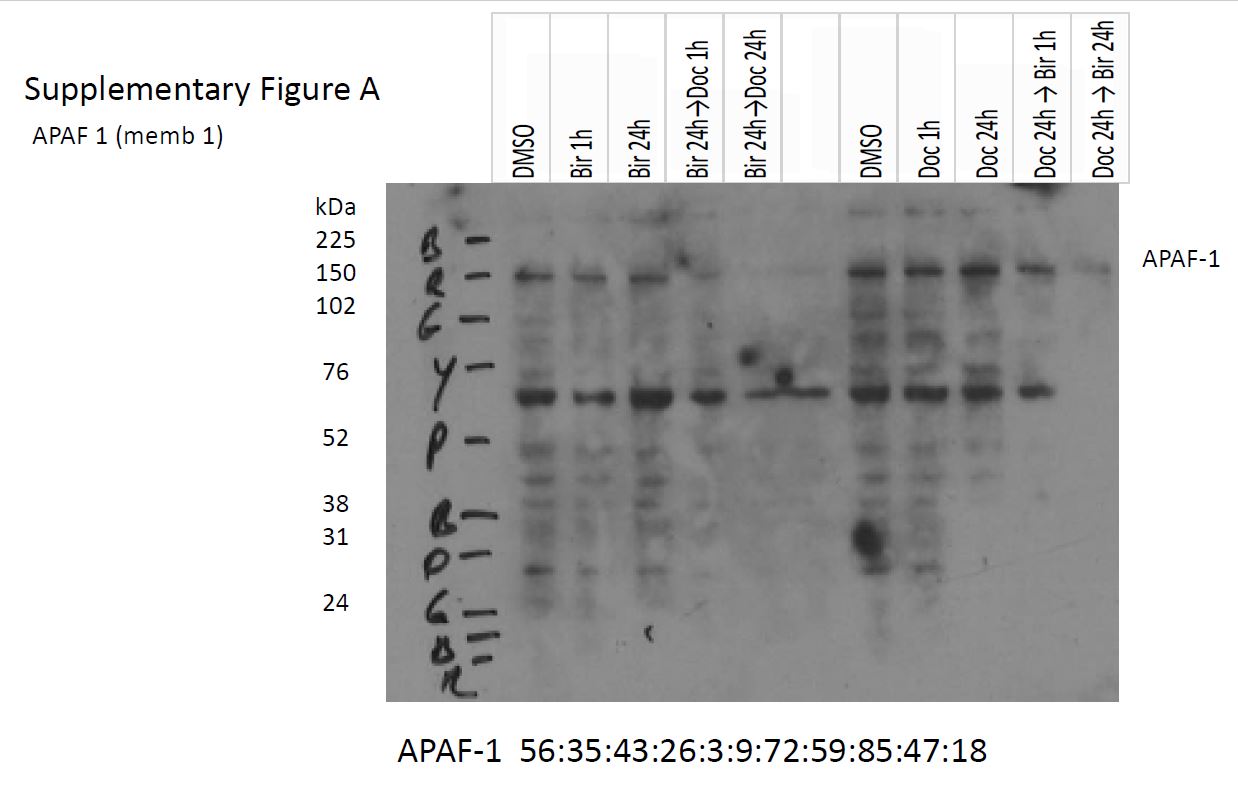


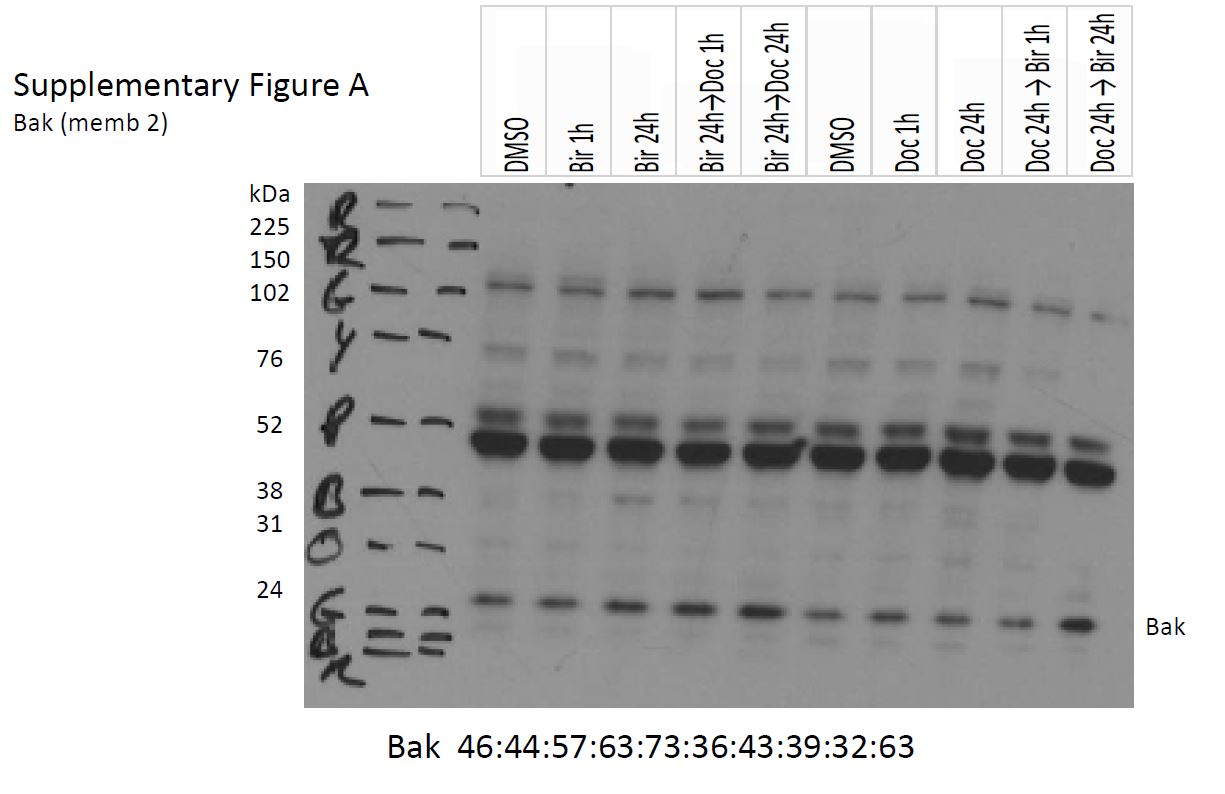


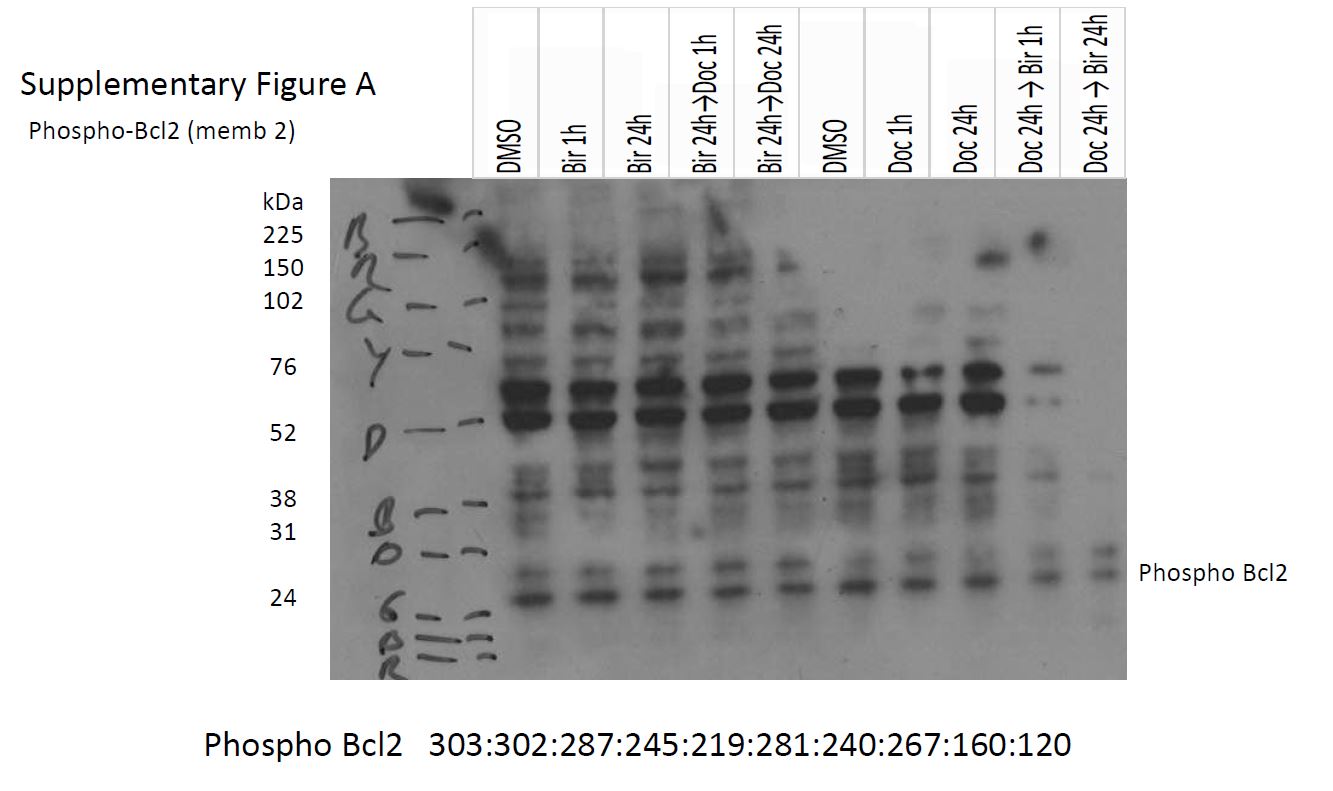


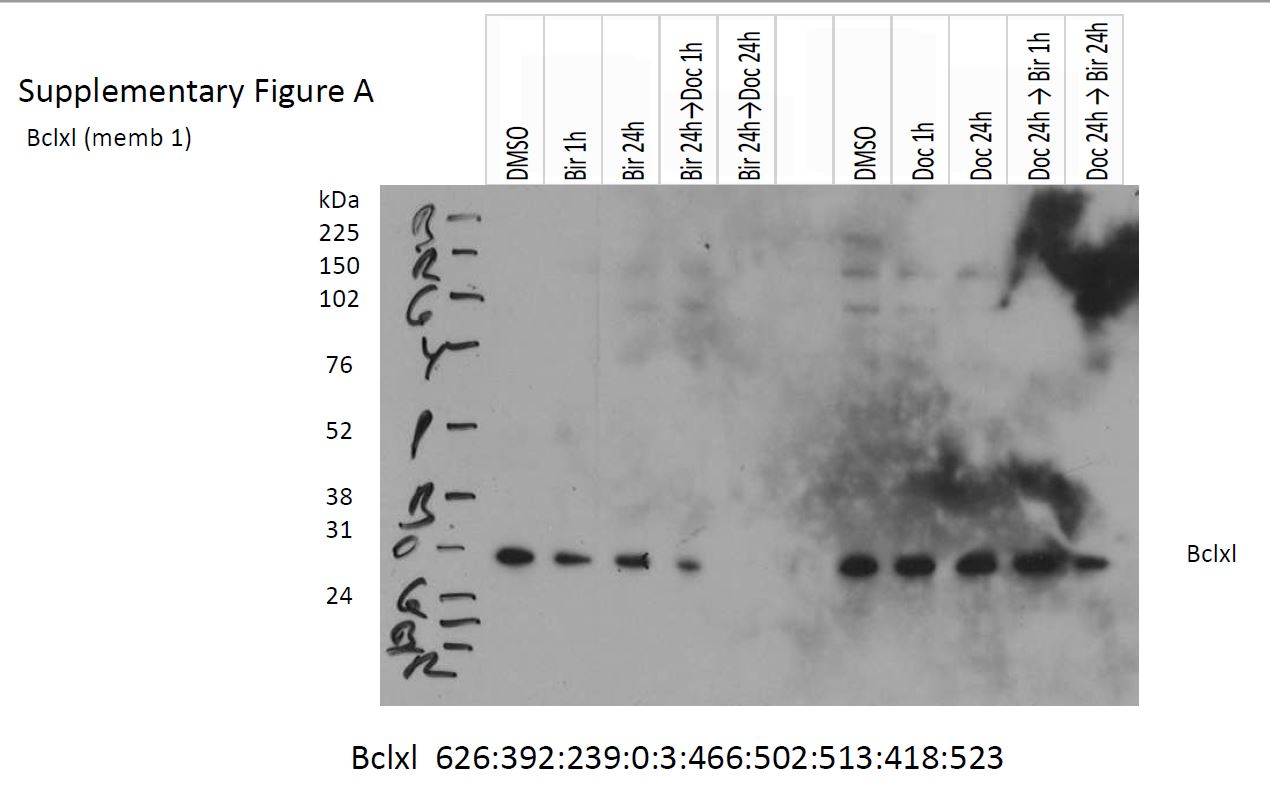


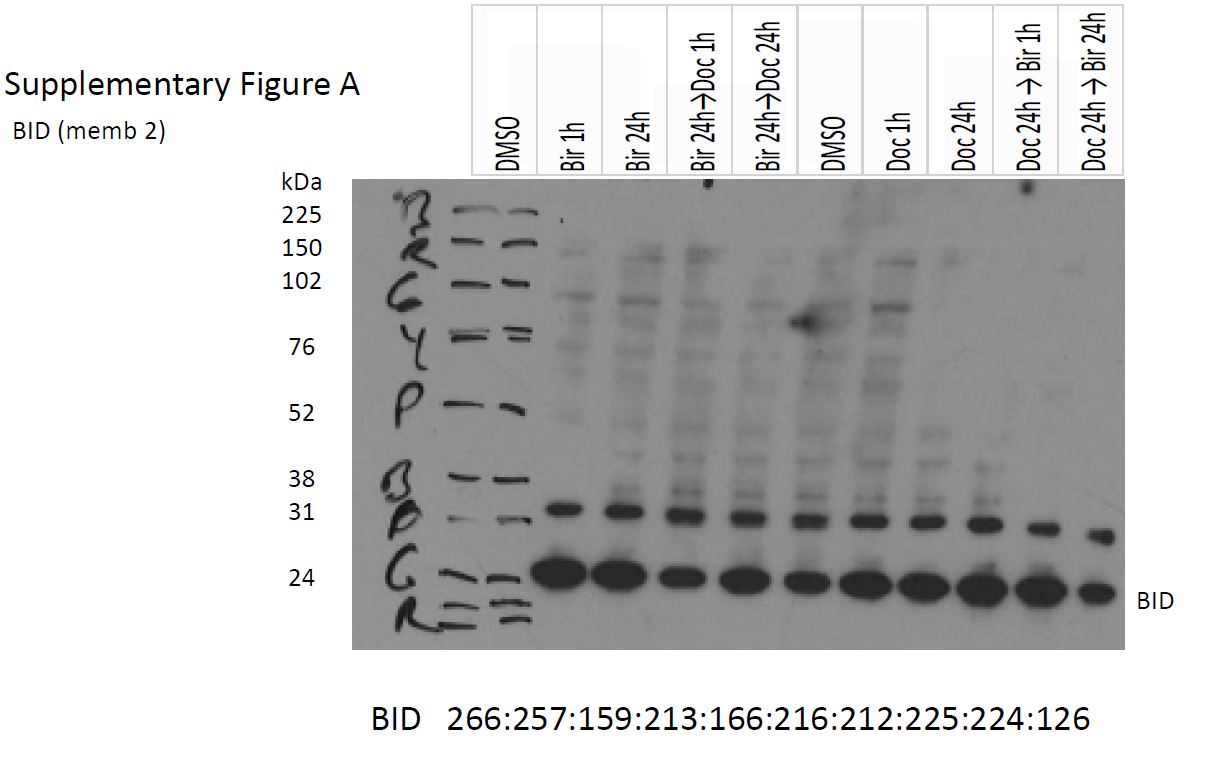


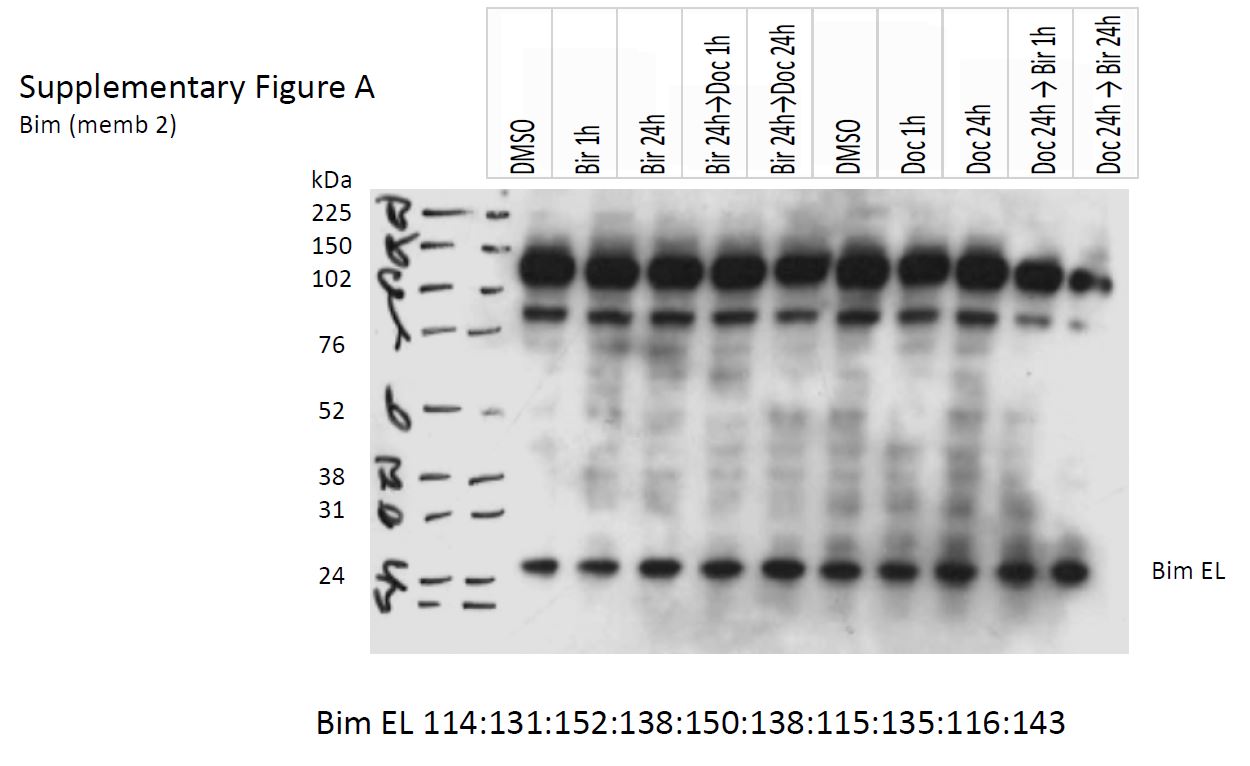


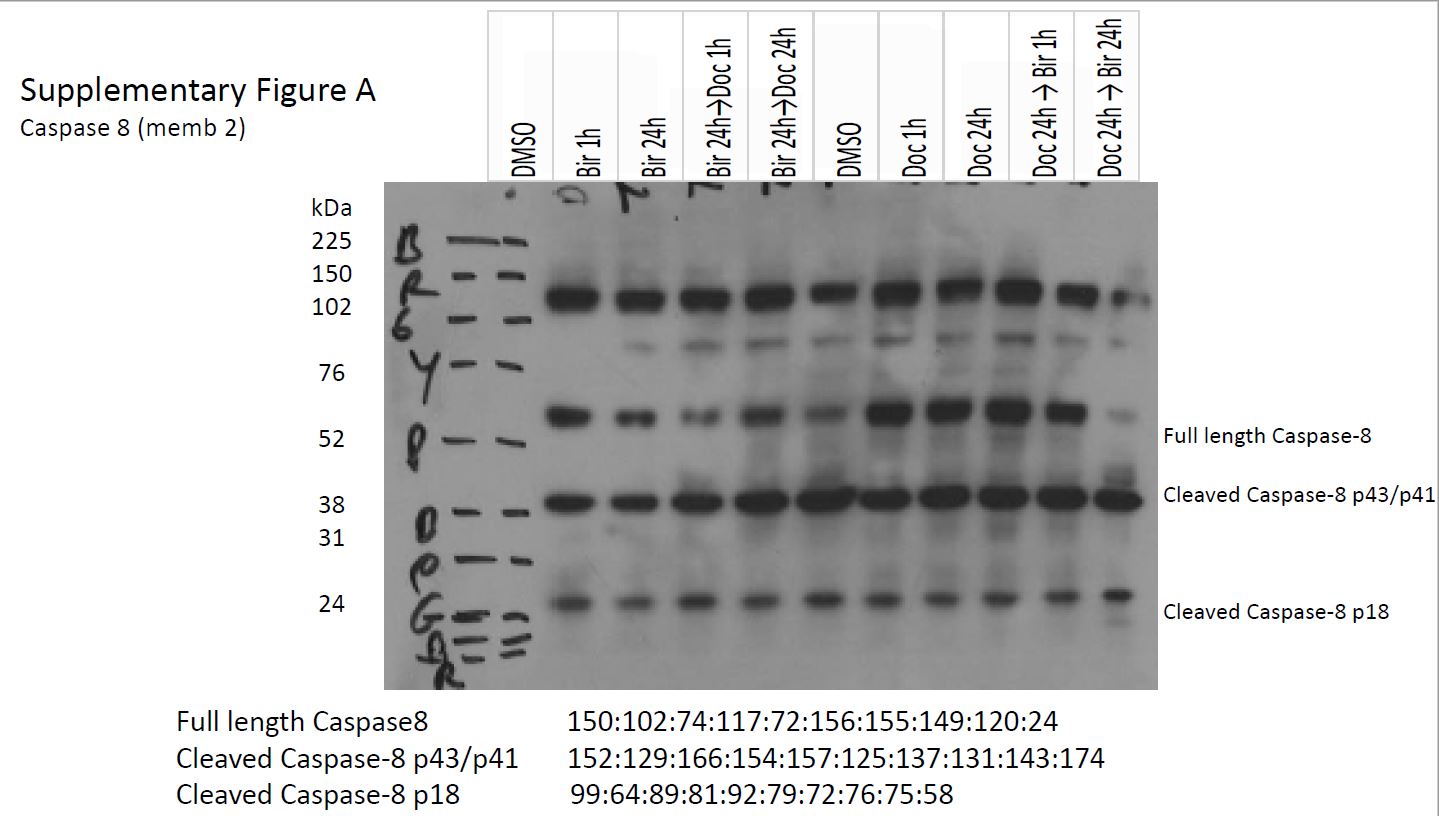


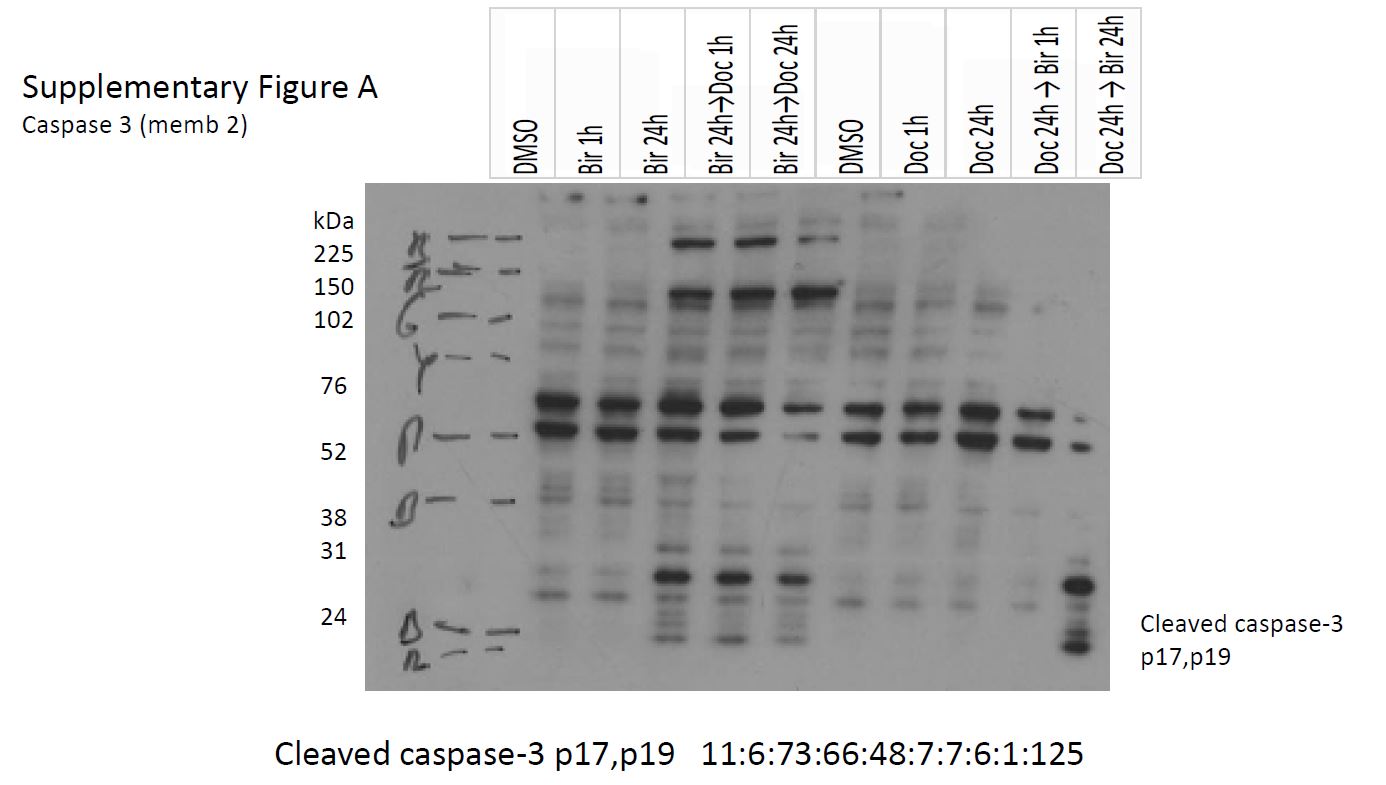


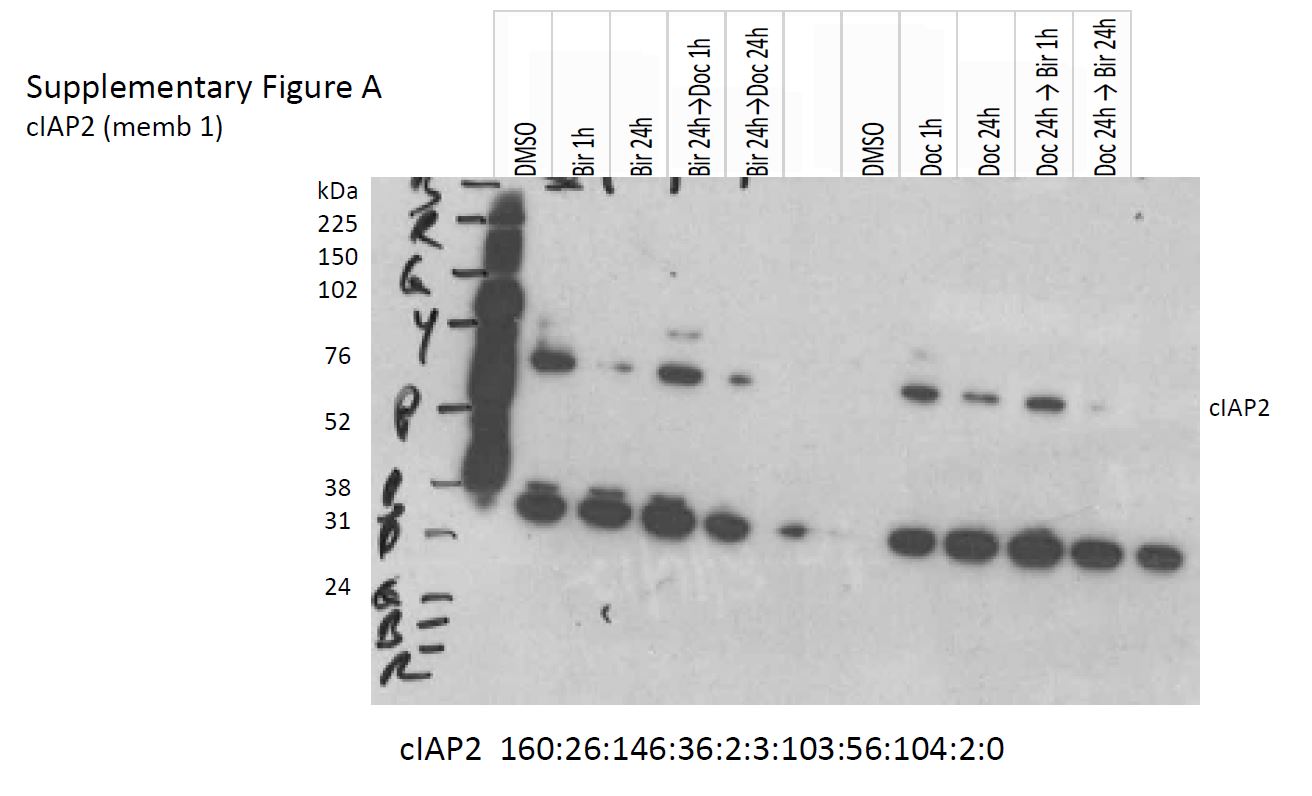


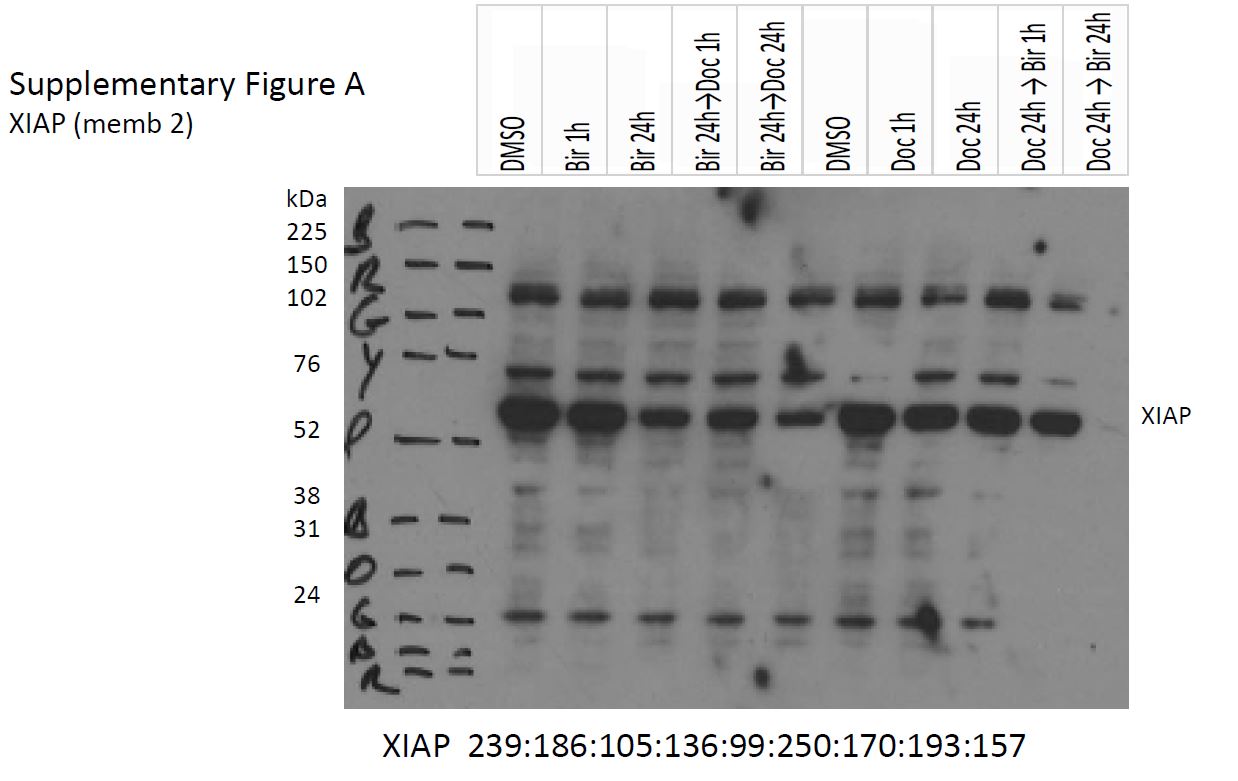


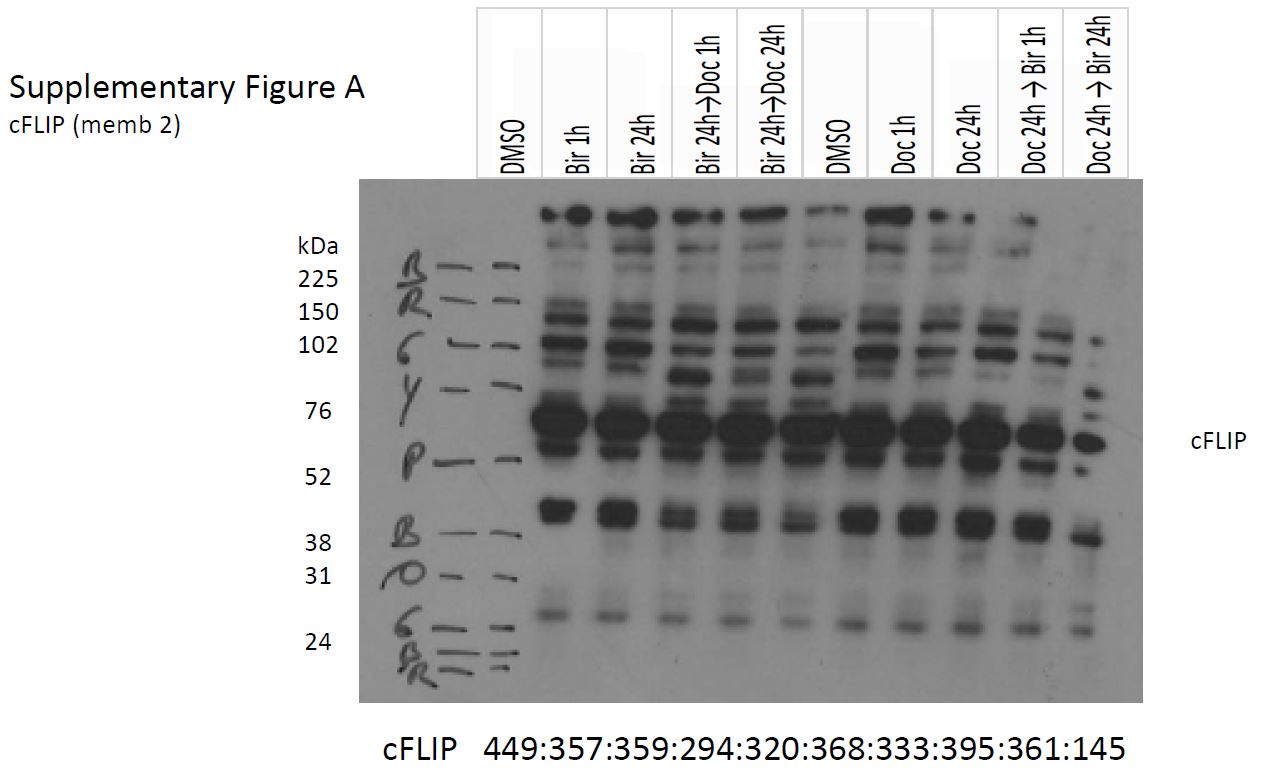


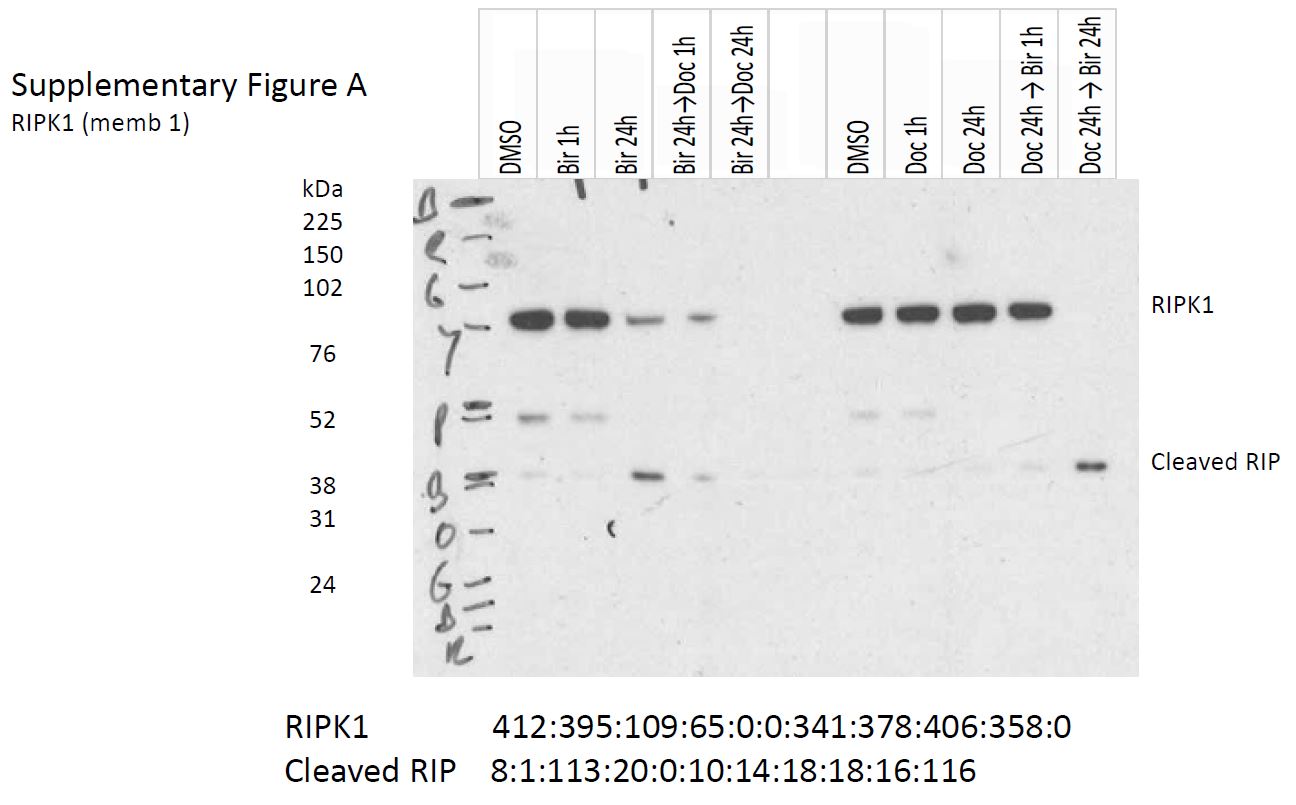


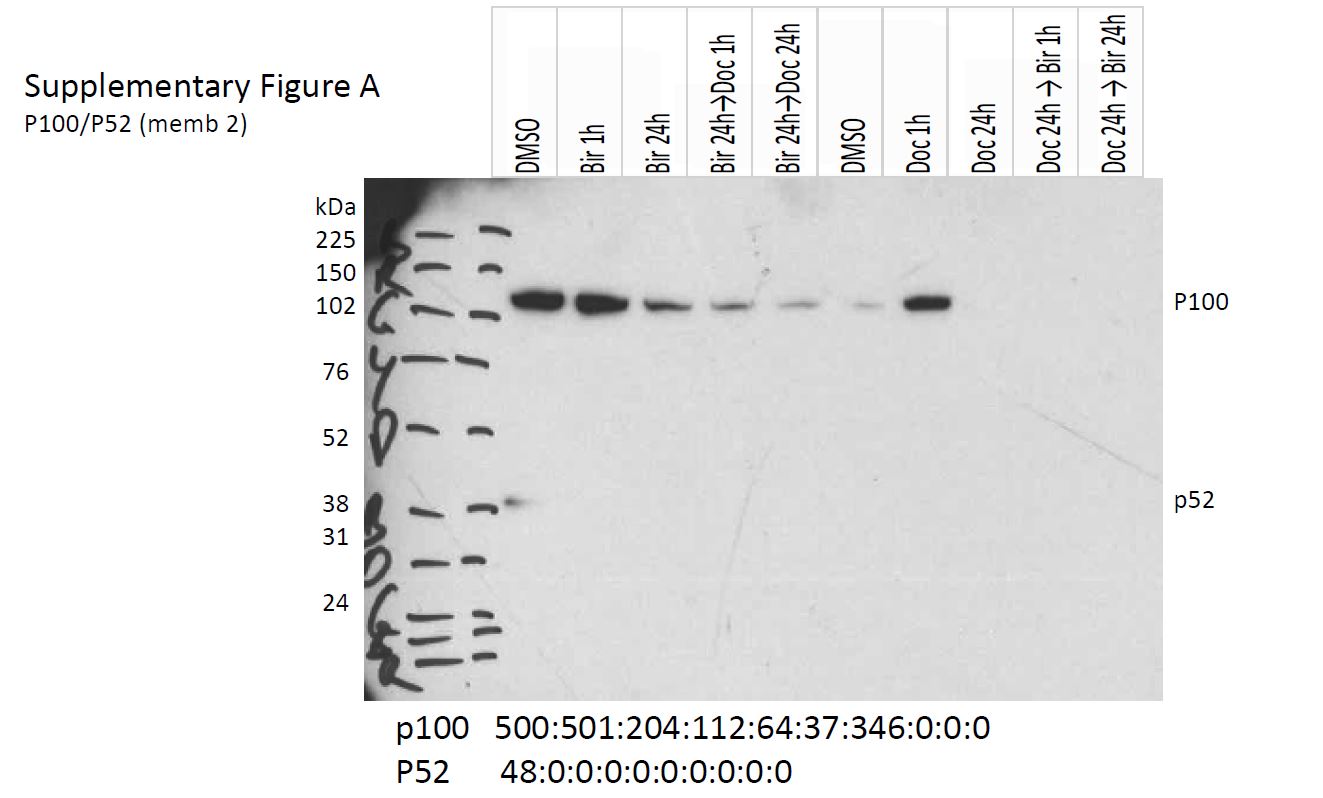


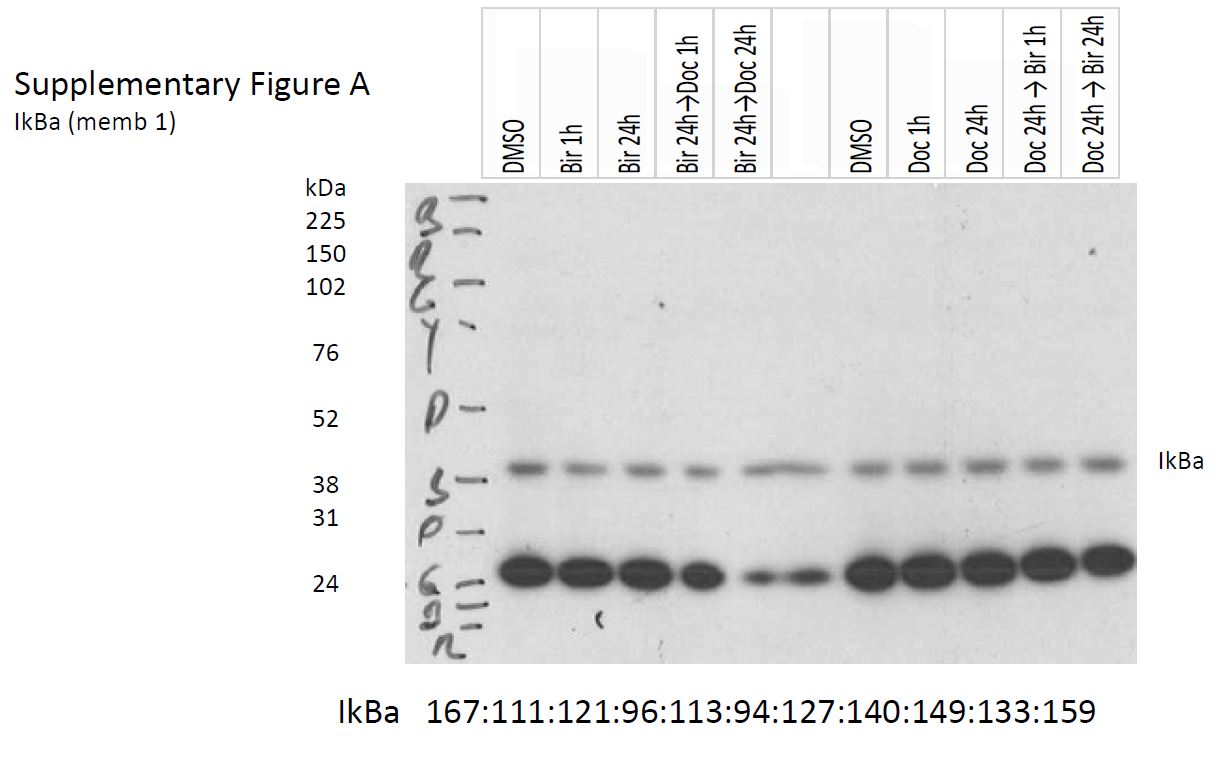


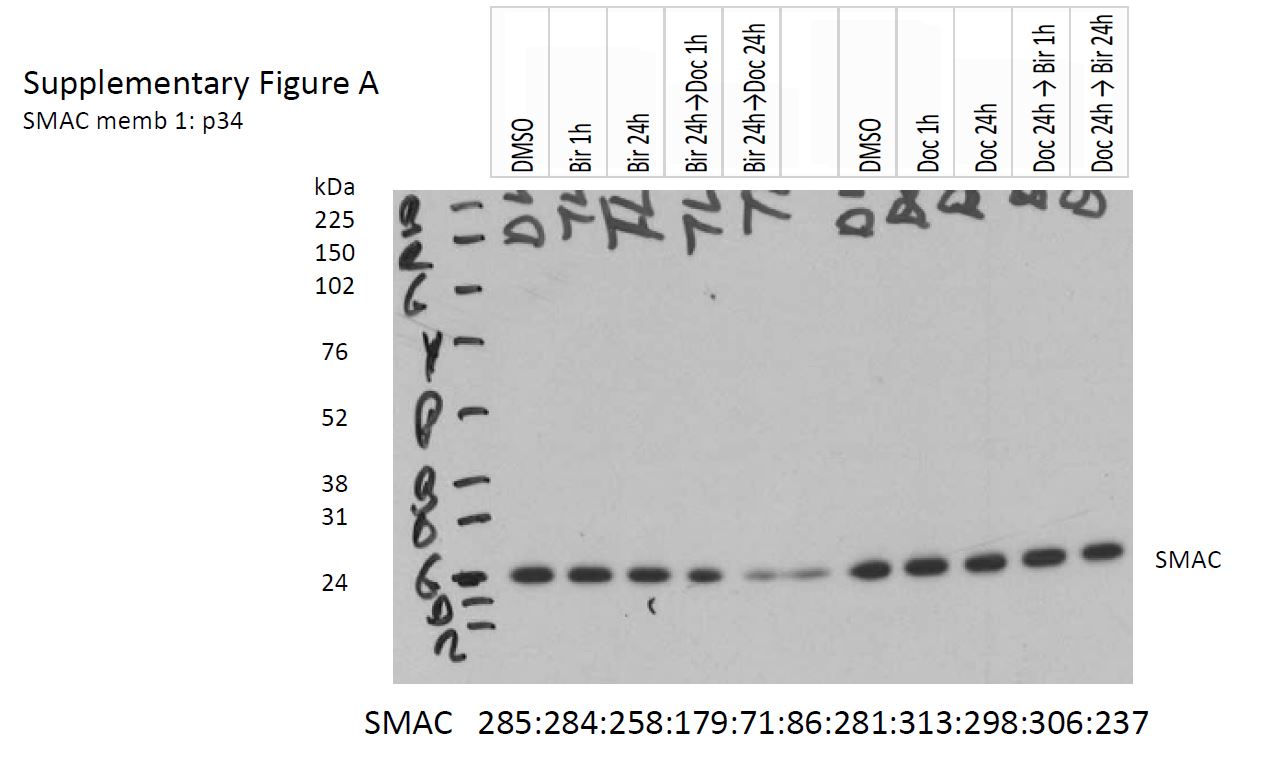


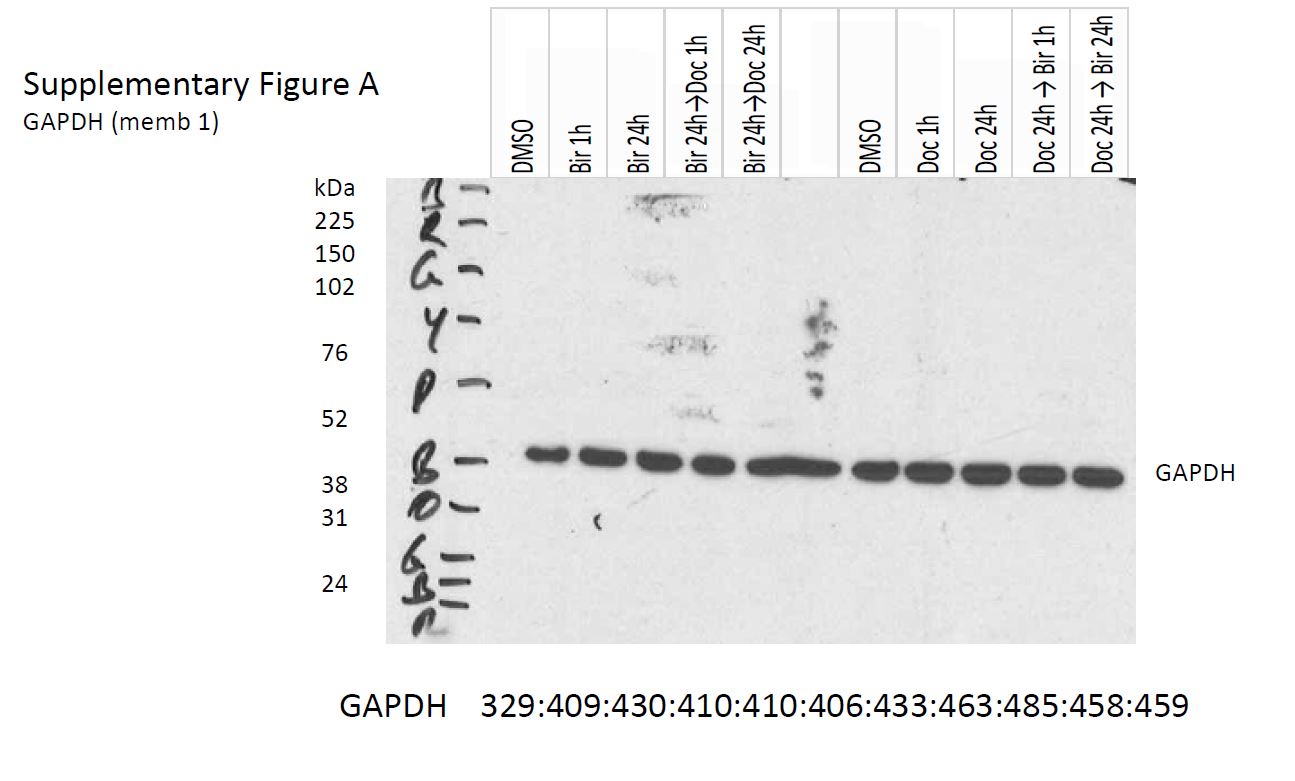


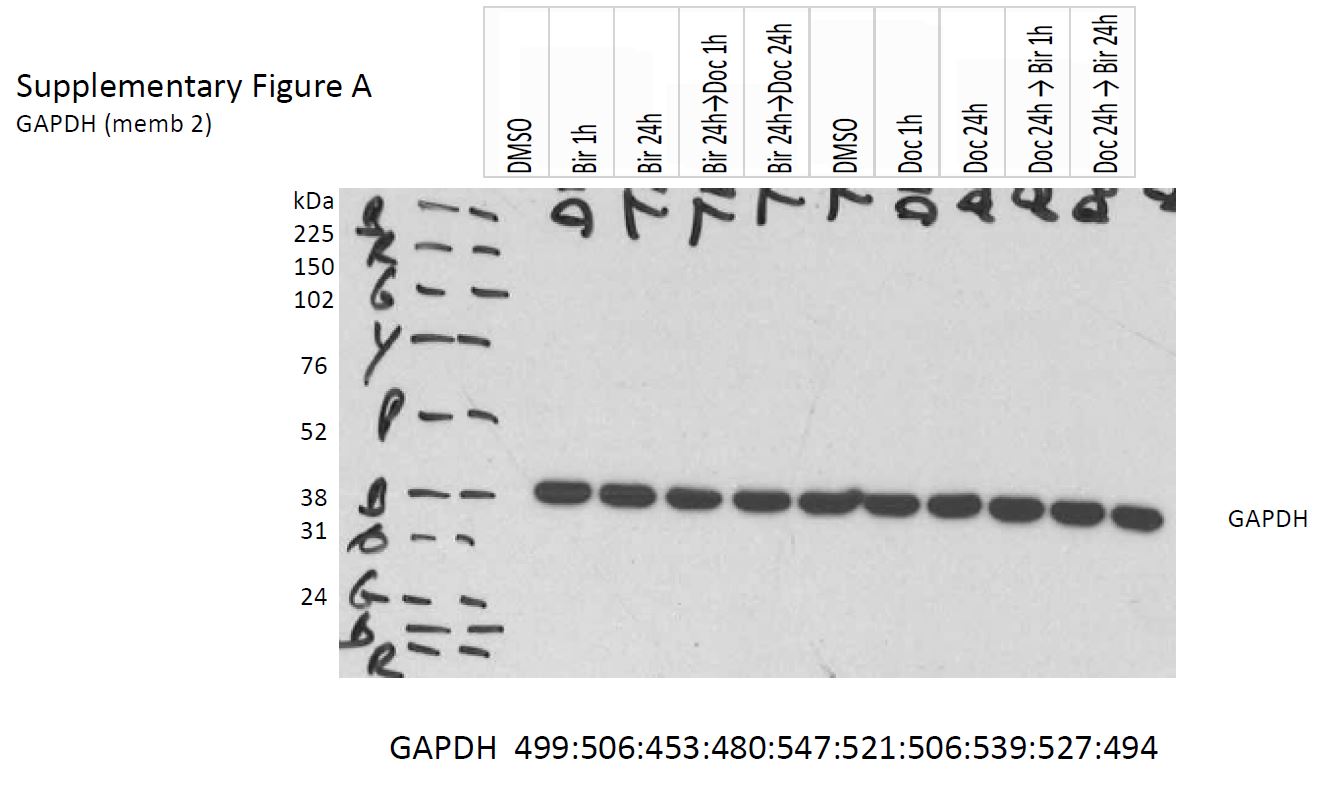


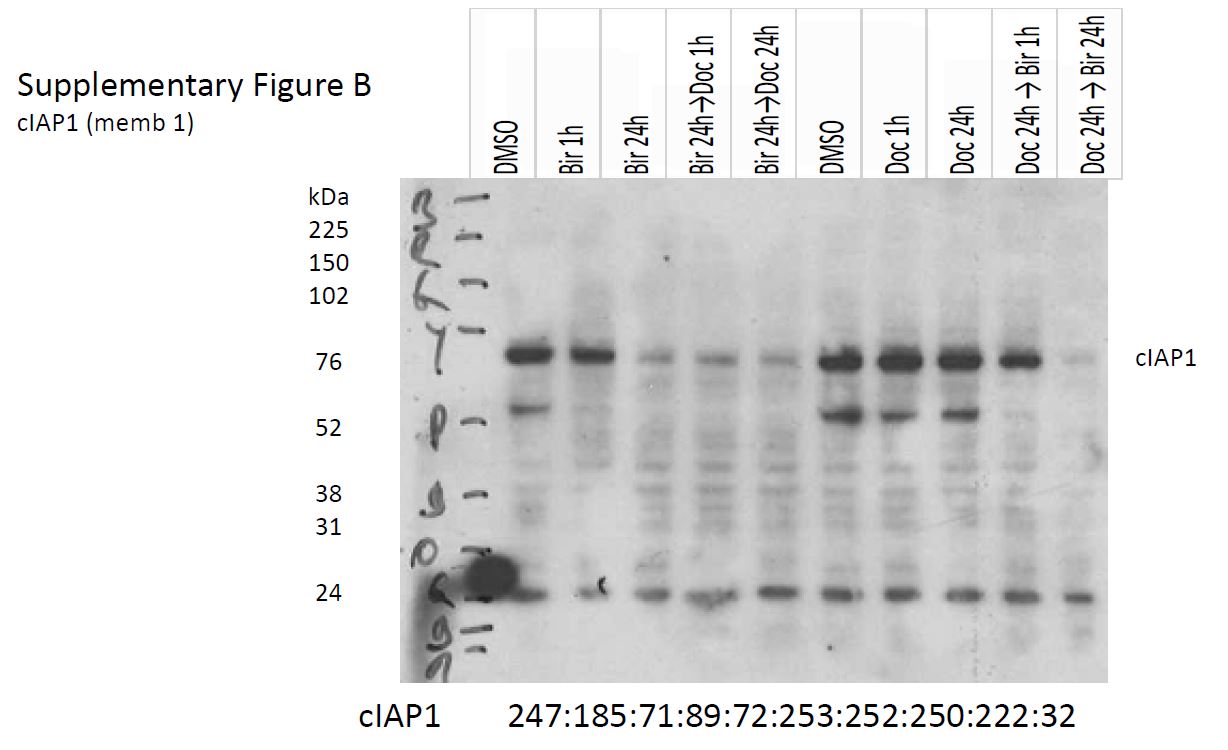


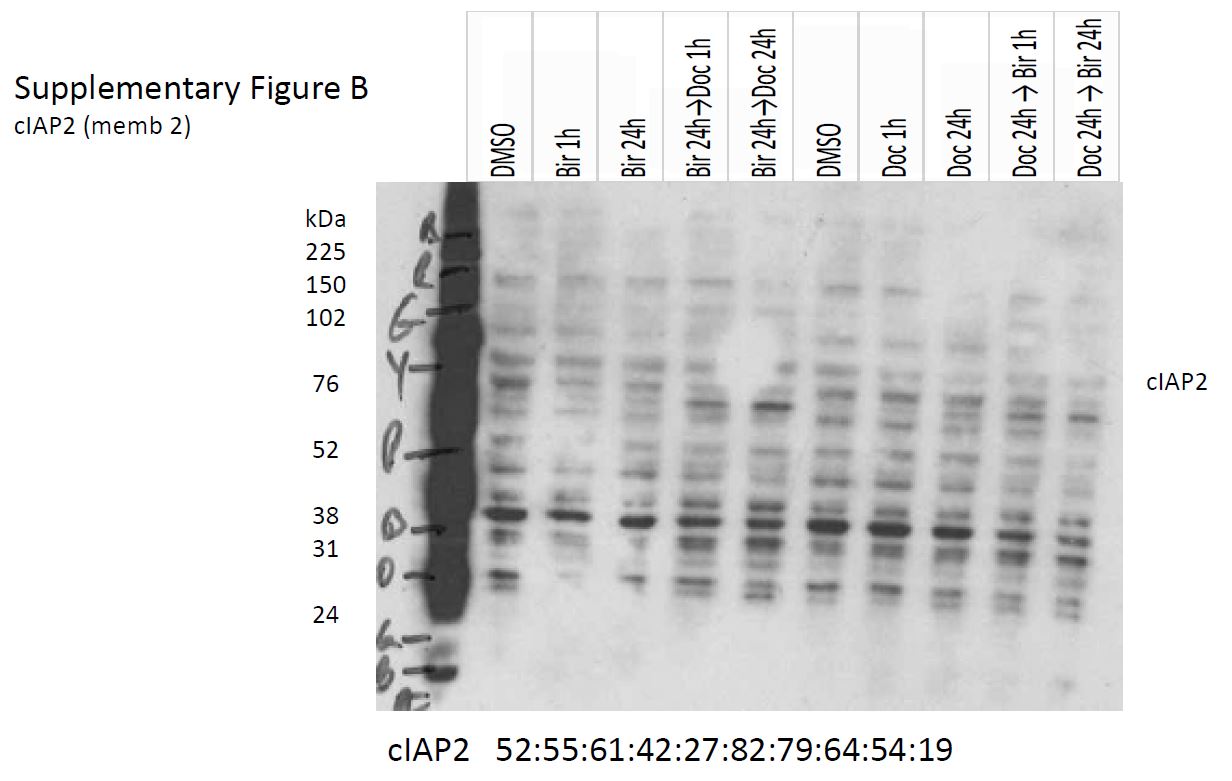


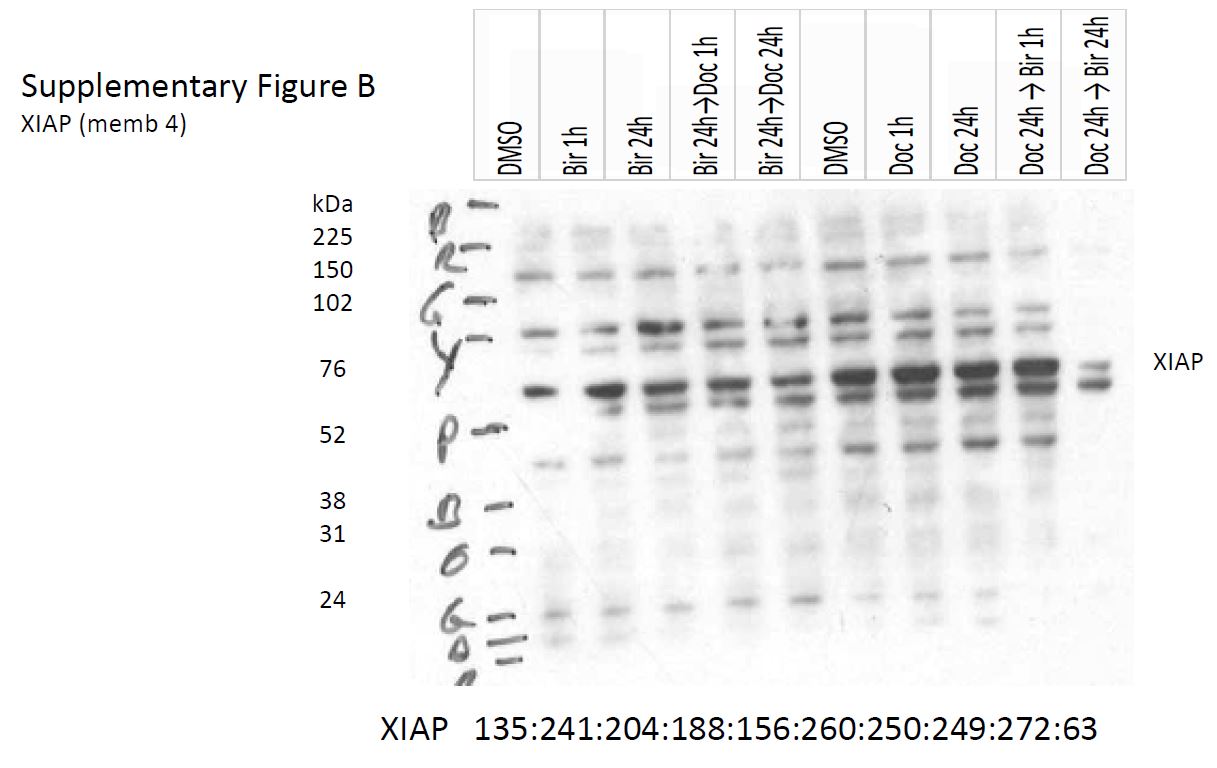


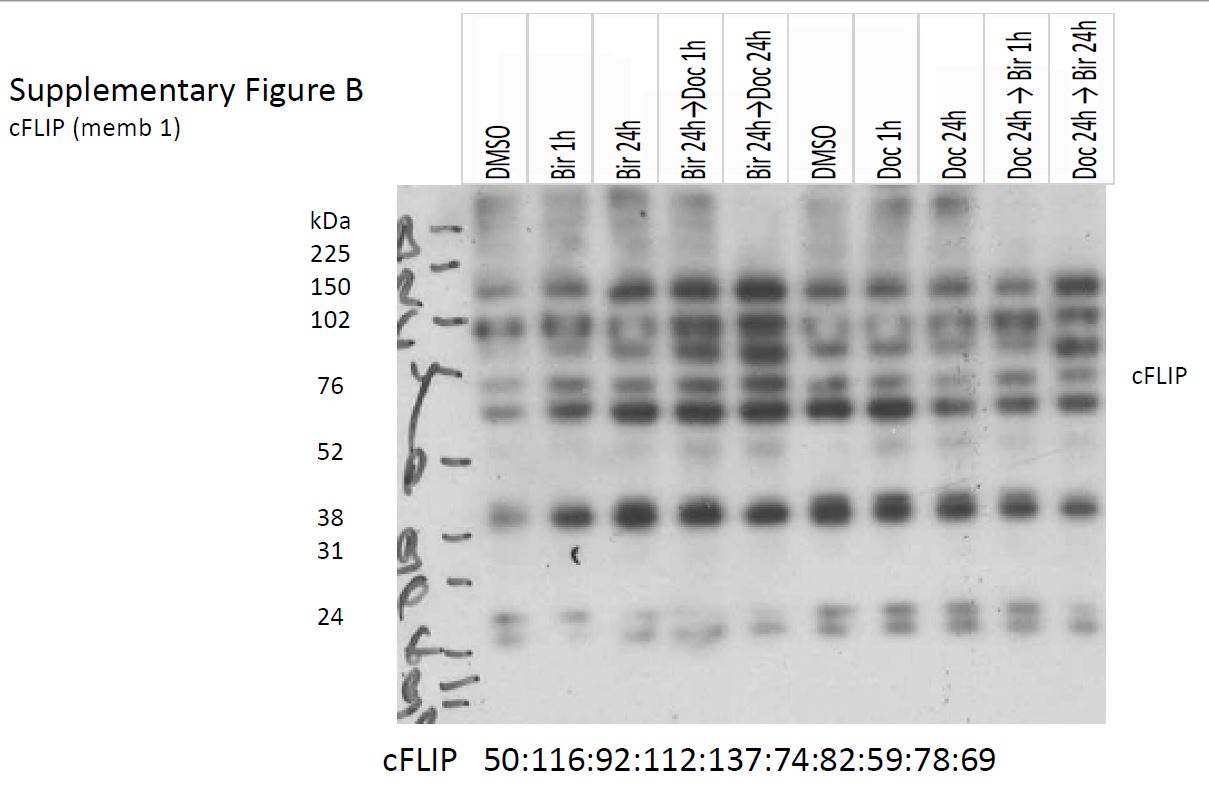


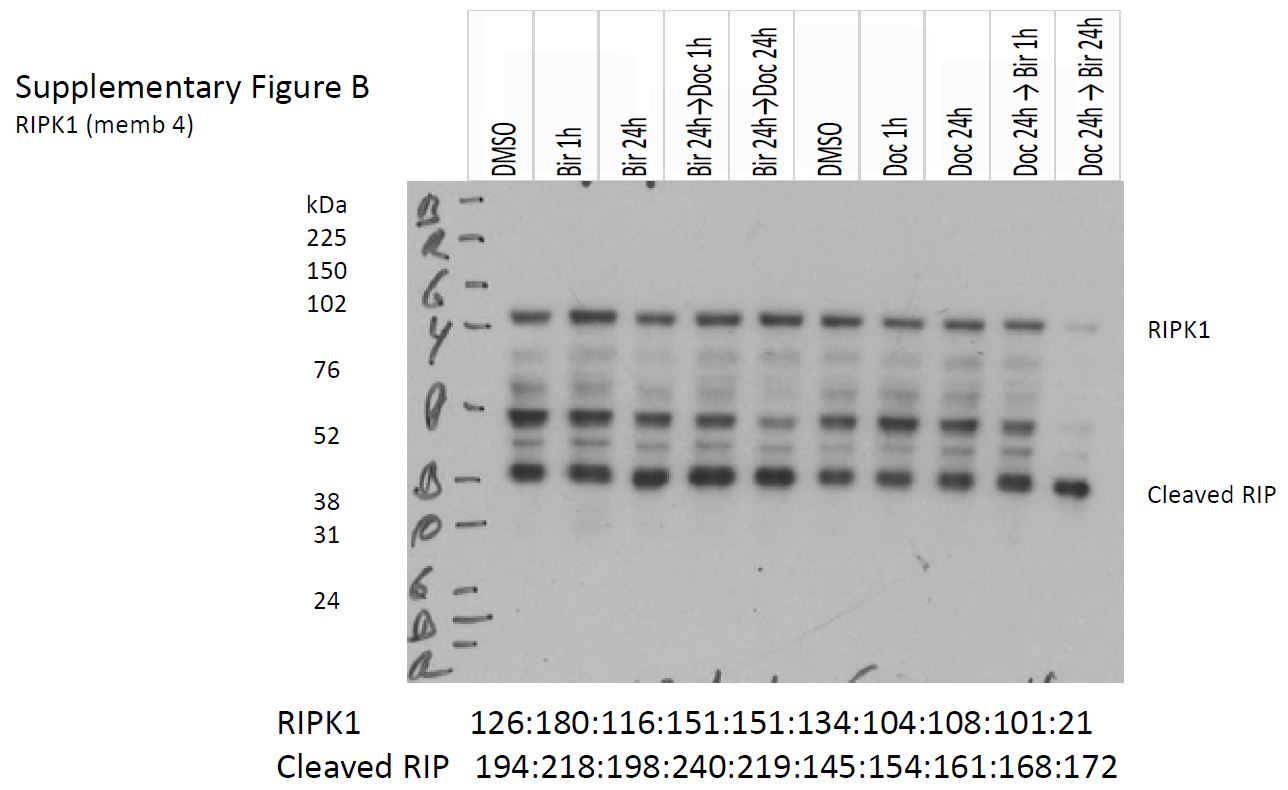


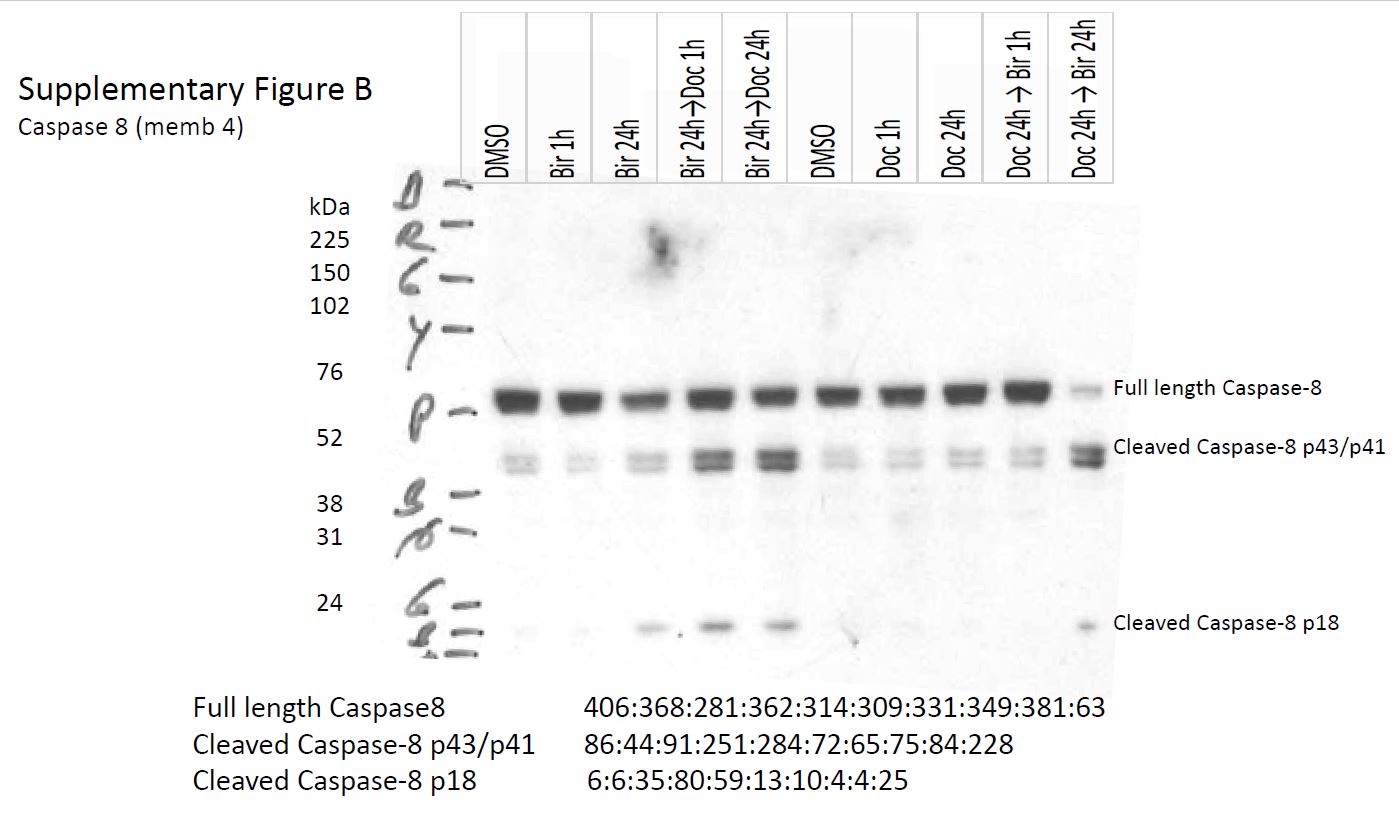


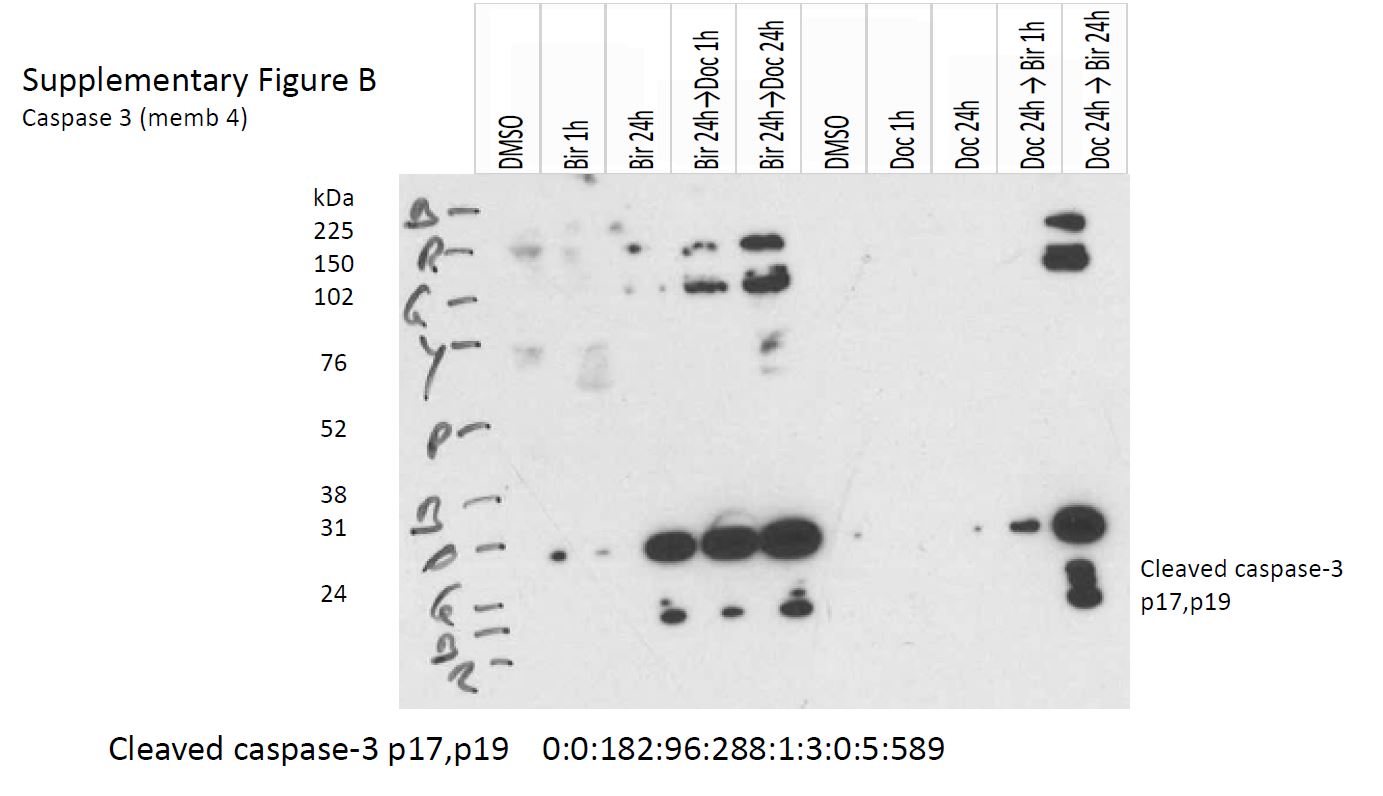


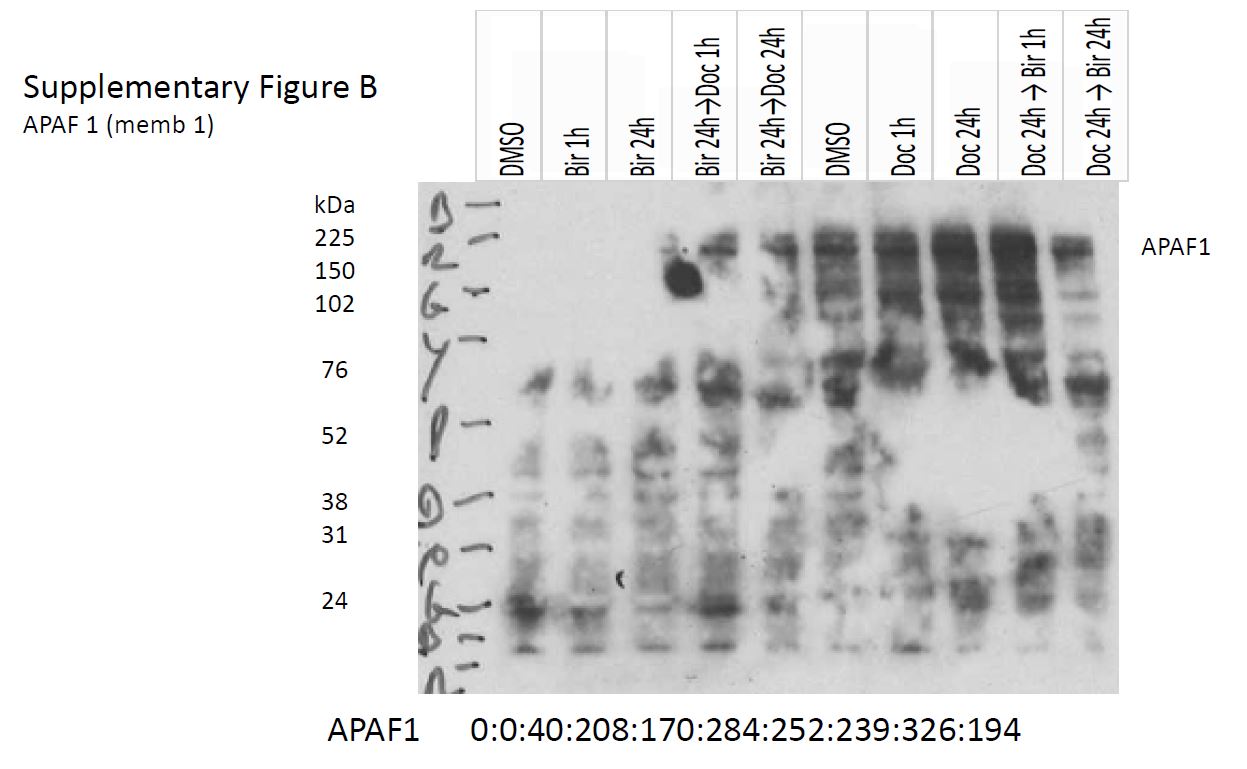


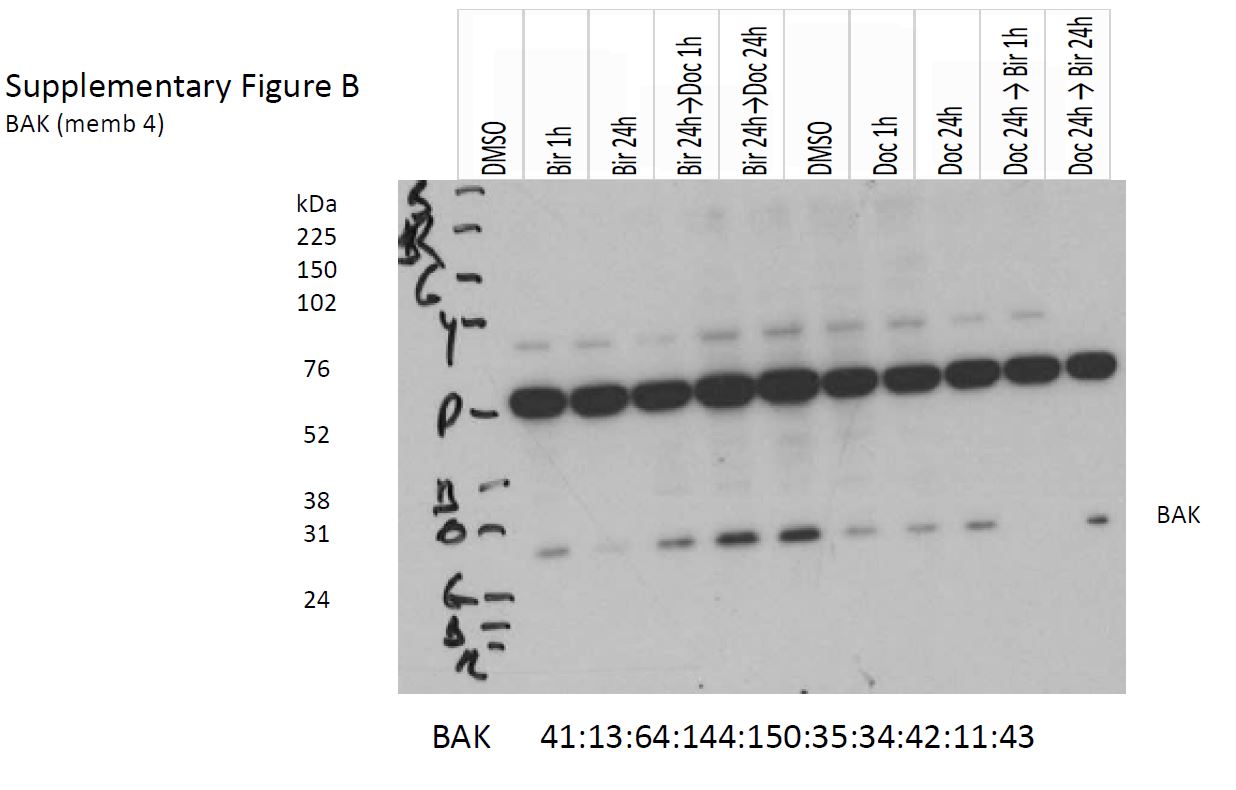


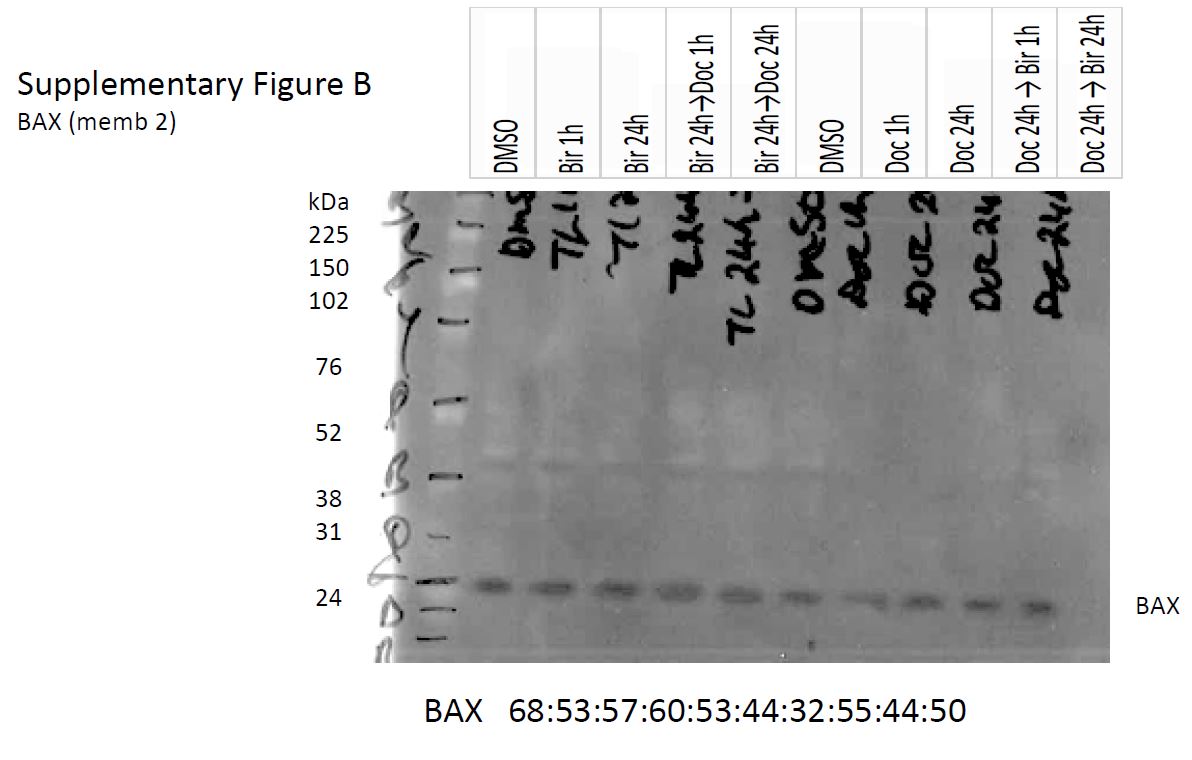


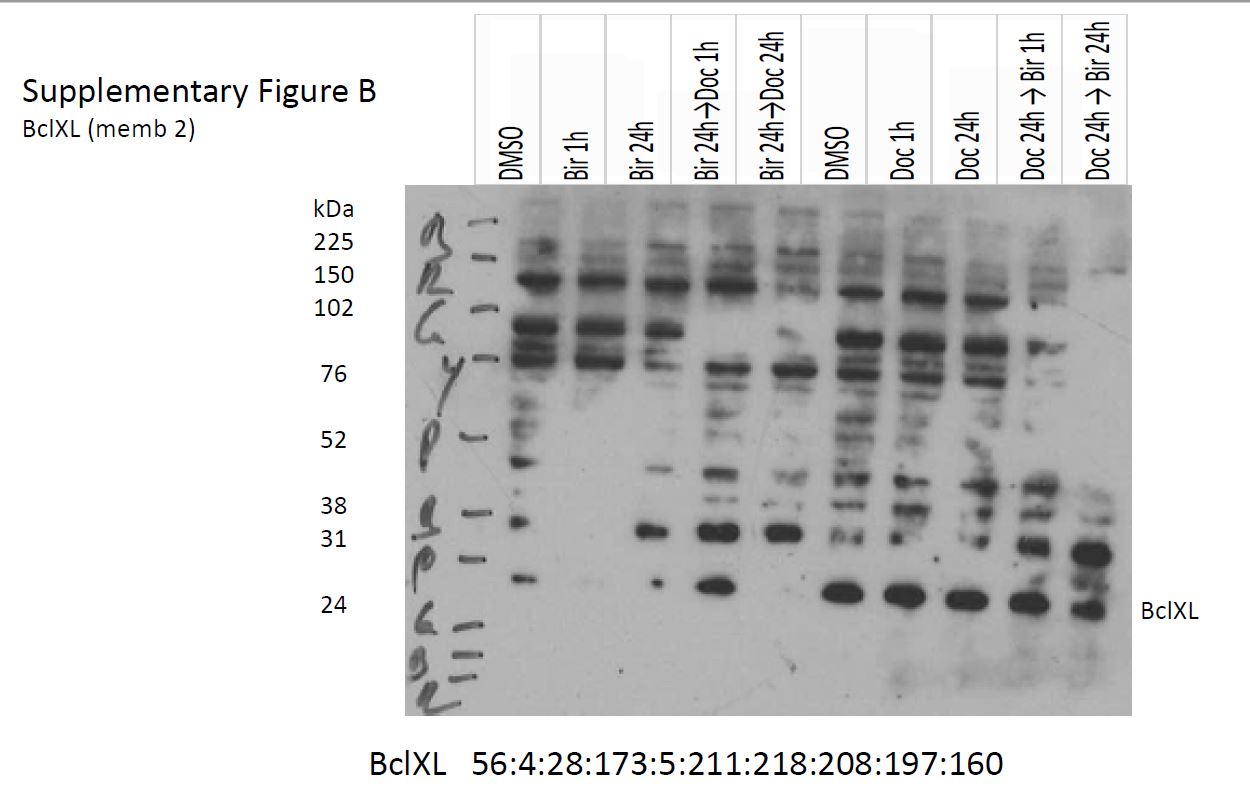


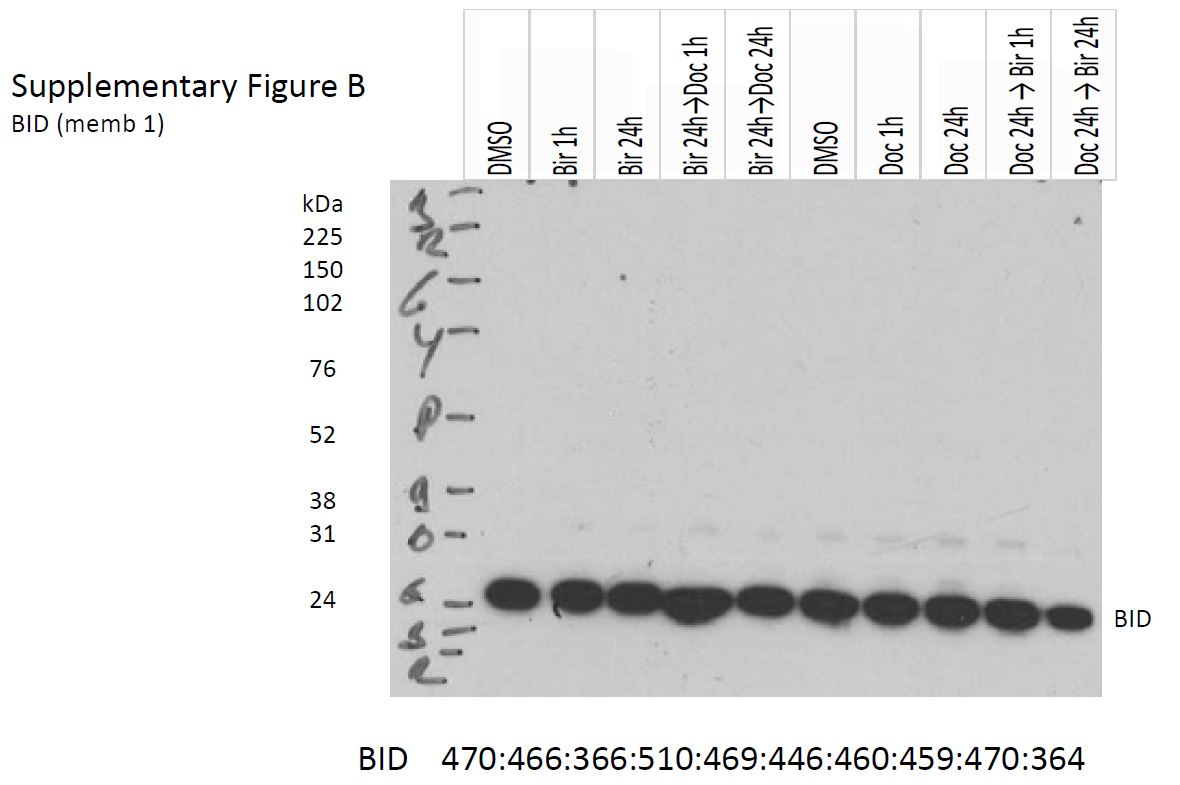


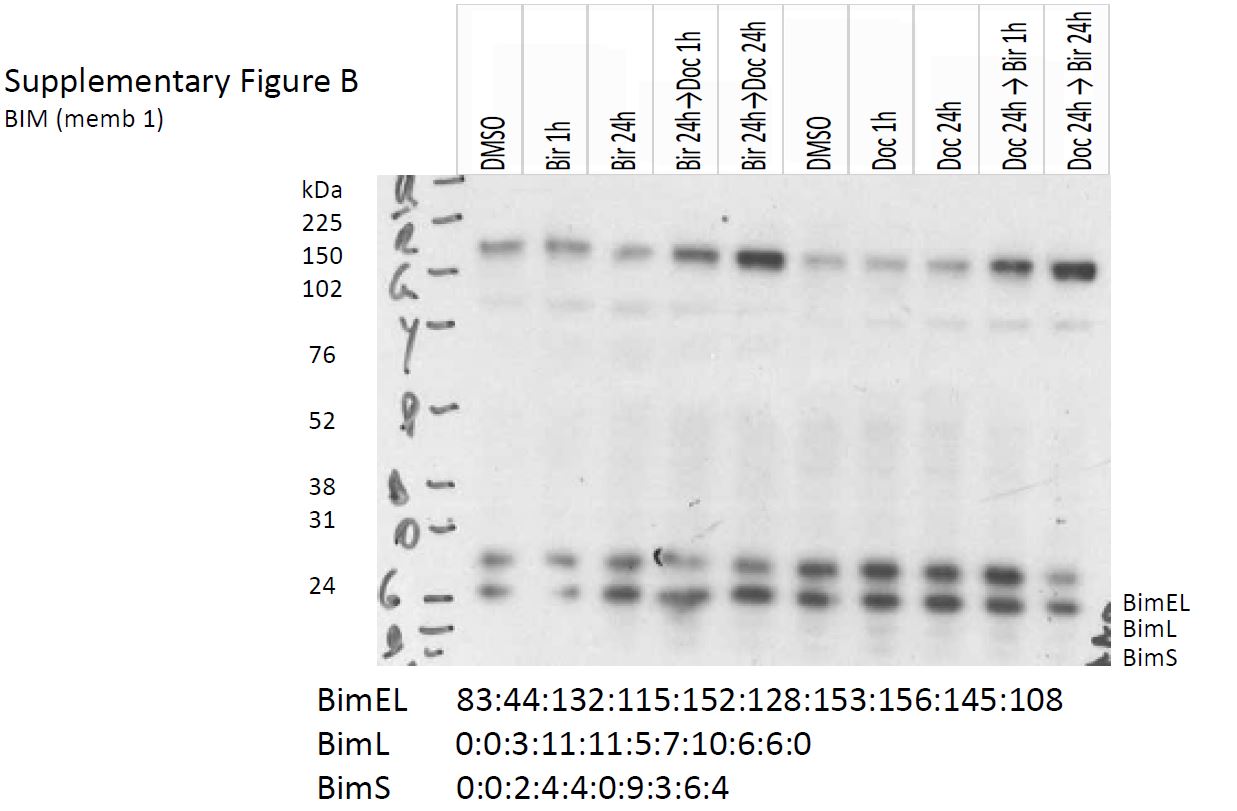


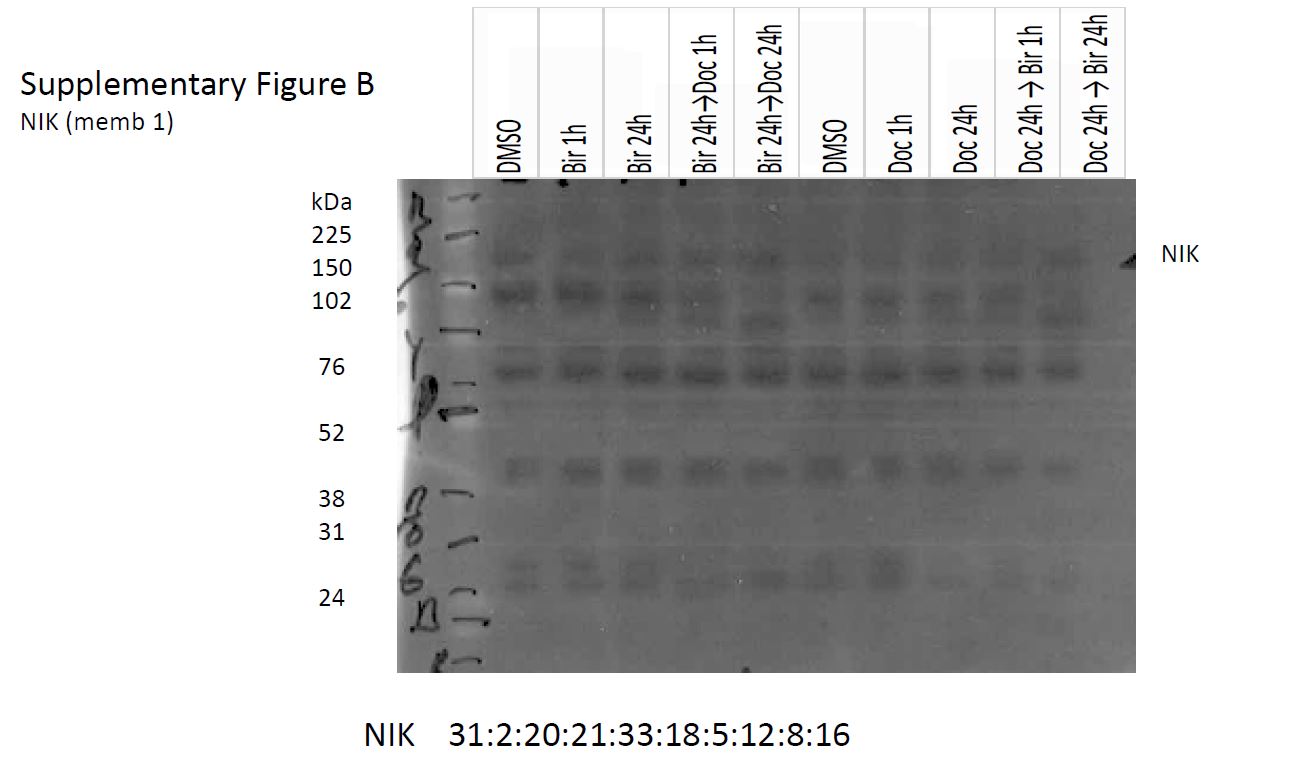


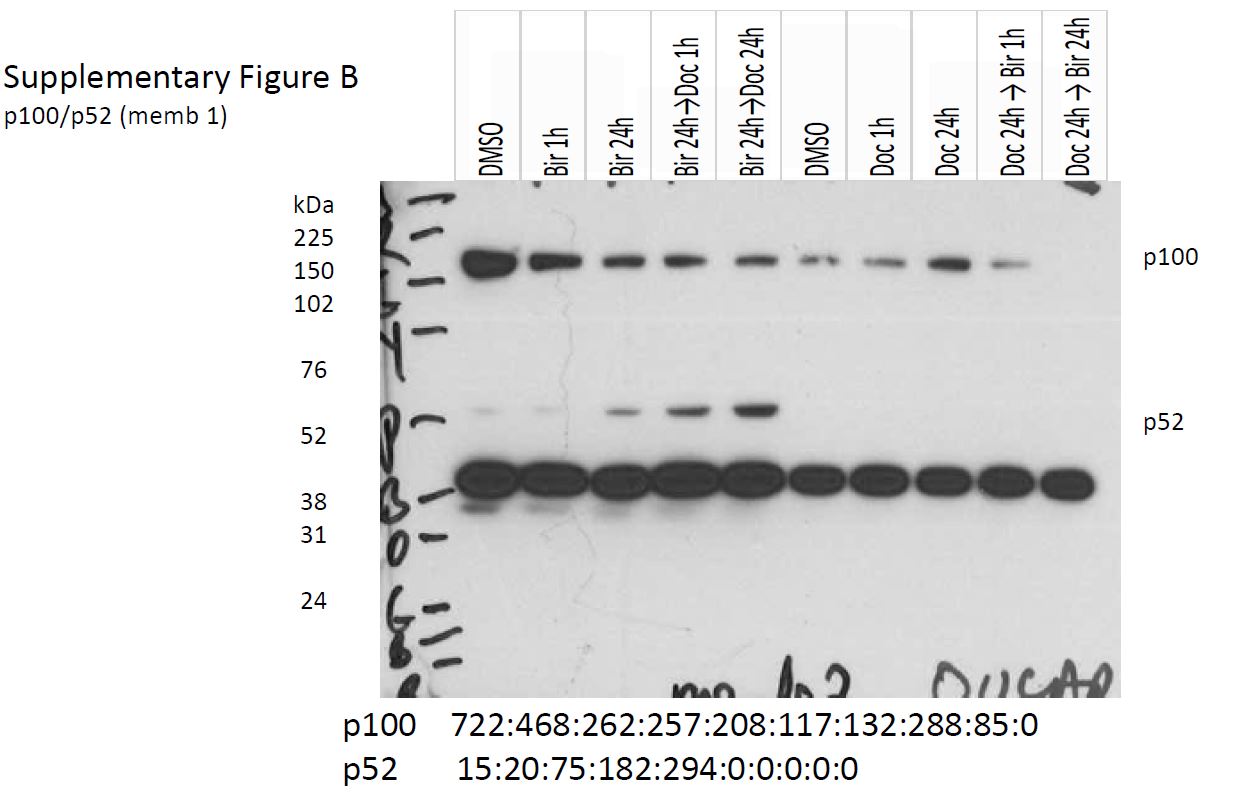


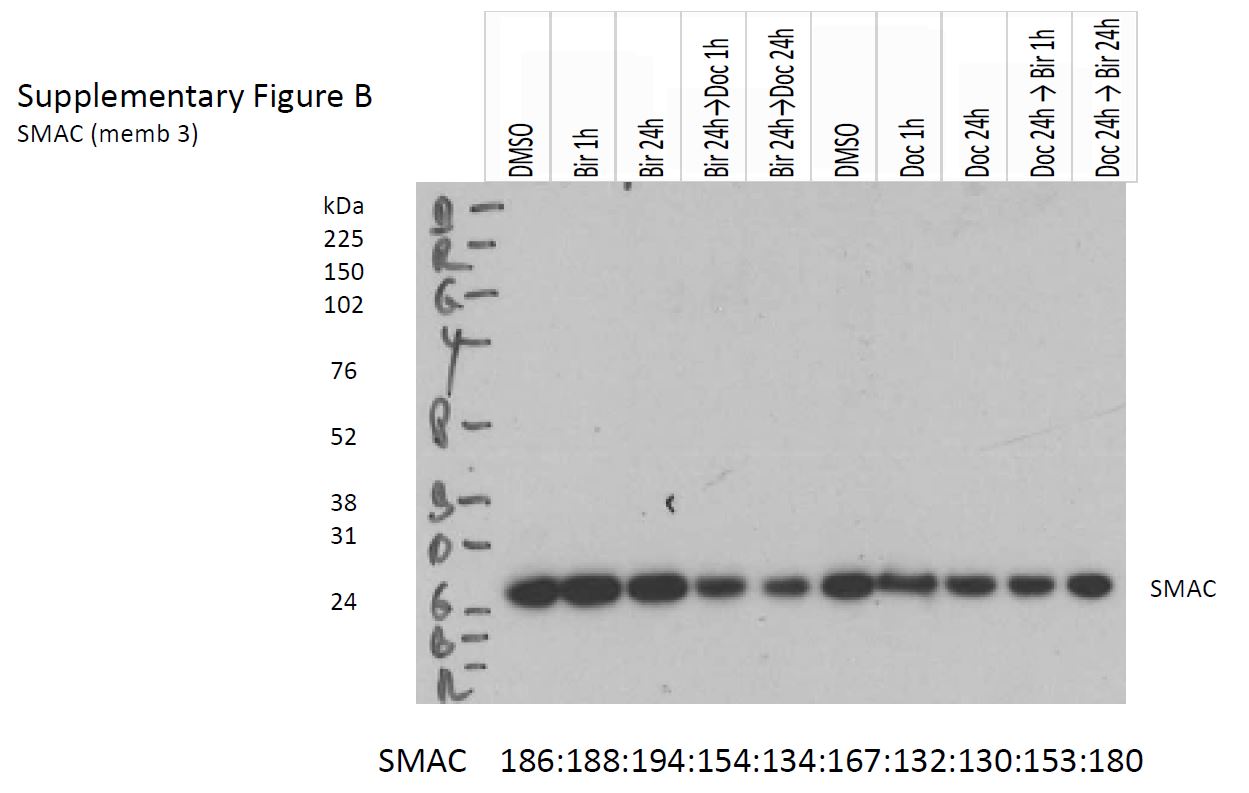


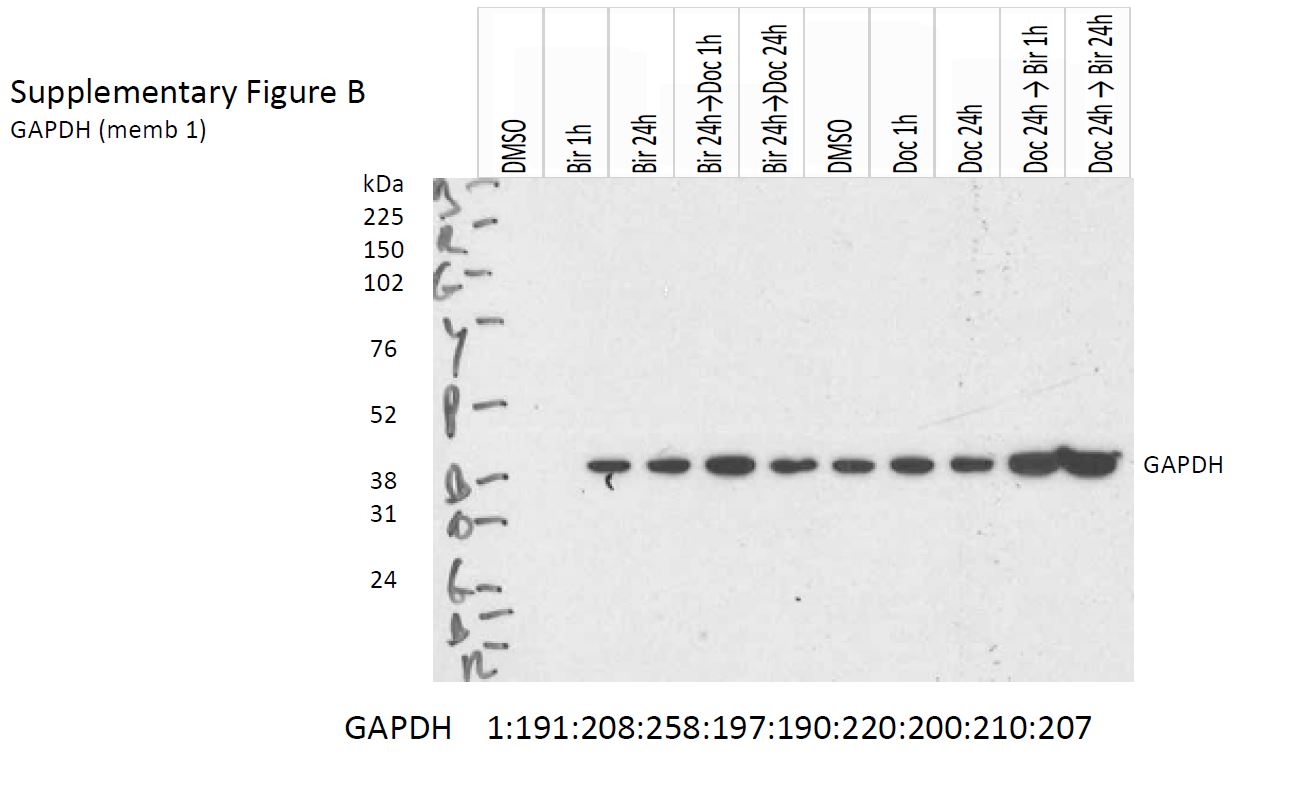


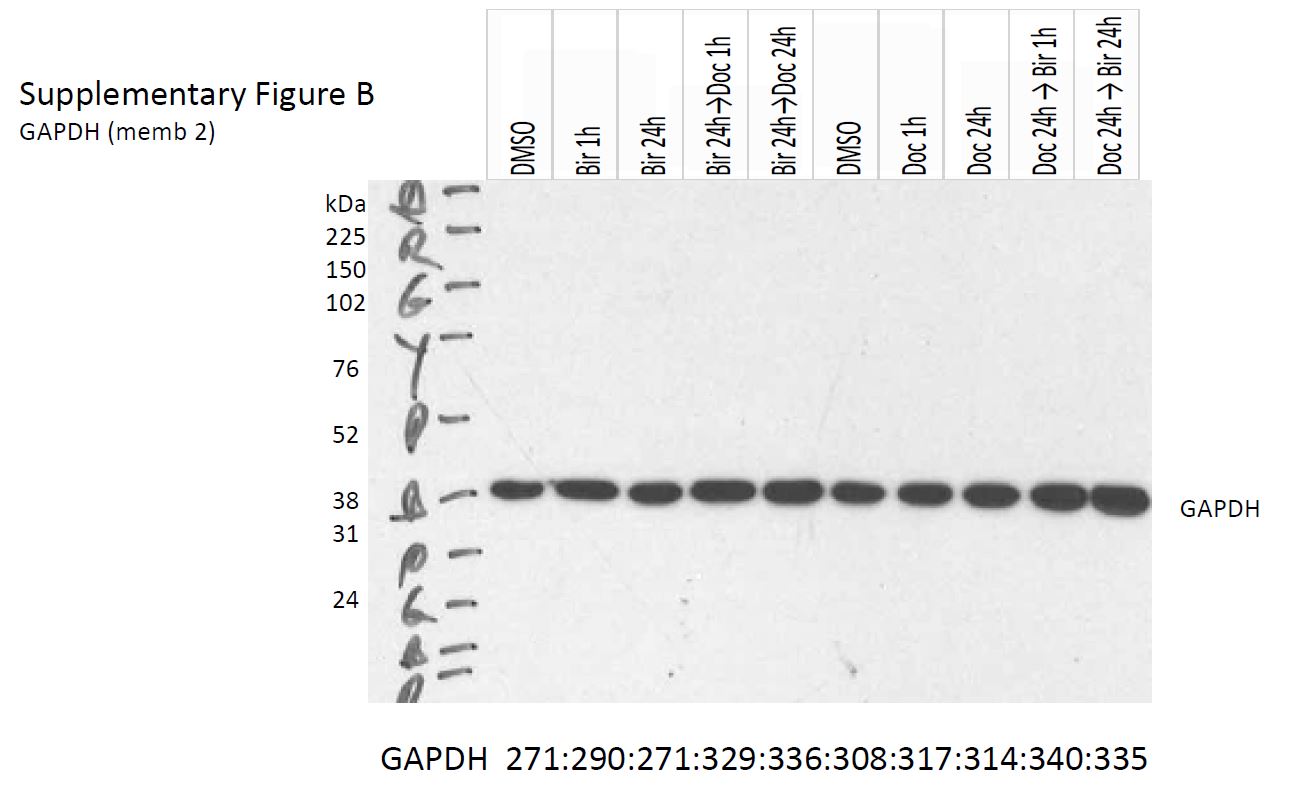


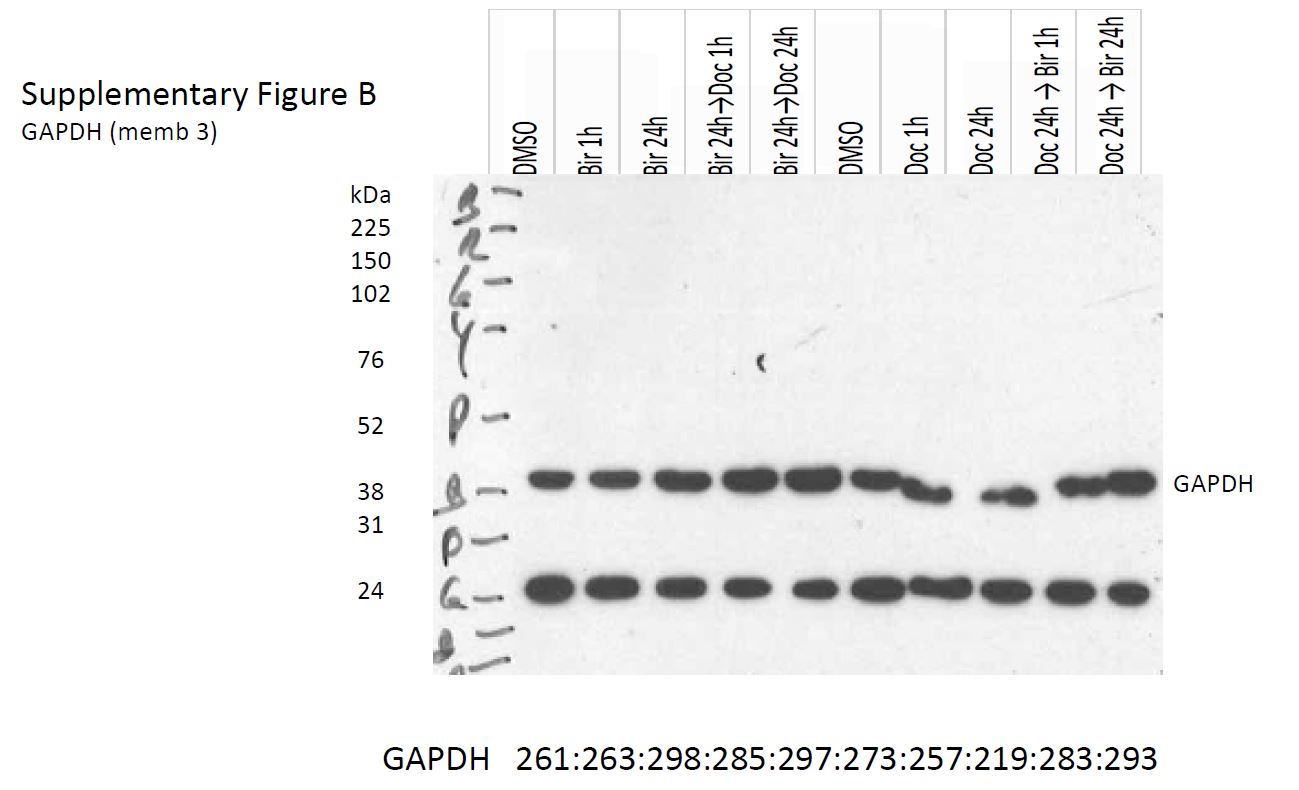


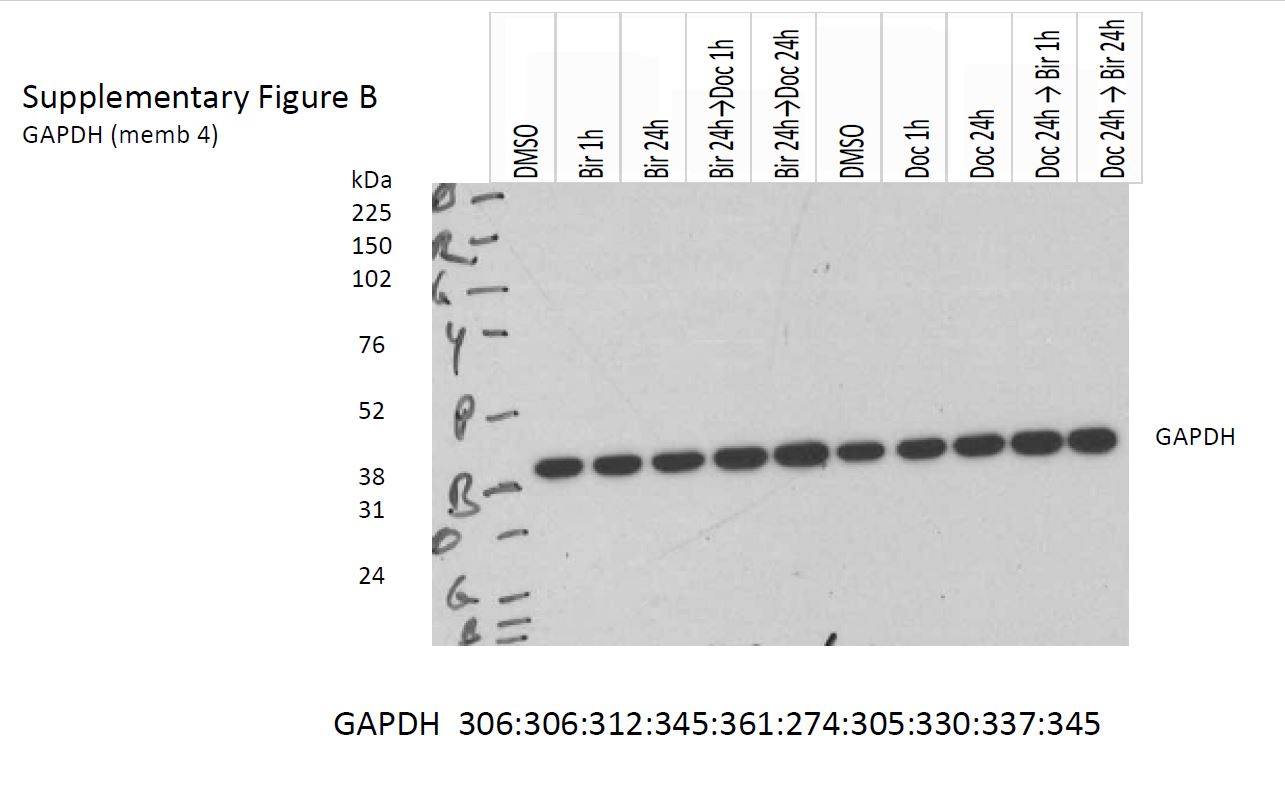


**Figure S4.** original western blot images of Figure S1A and S1B.

**Publisher’s Note:** MDPI stays neutral with regard to jurisdictional claims in published maps and institutional affiliations.

| 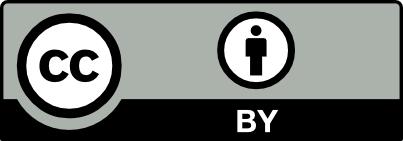 | © 2020 by the authors. Submitted for possible open access publication under the terms and conditions of the Creative Commons Attribution (CC BY) license (http://creativecommons.org/licenses/by/4.0/). |
| --- | --- |
